# A Knowledge-Based Weighting Framework to Boost the Power of Genome-Wide Association Studies

**DOI:** 10.1371/journal.pone.0014480

**Published:** 2010-12-31

**Authors:** Miao-Xin Li, Pak C. Sham, Stacey S. Cherny, You-Qiang Song

**Affiliations:** 1 Department of Biochemistry, The University of Hong Kong, Hong Kong, China; 2 Department of Psychiatry, The University of Hong Kong, Hong Kong, China; 3 The Centre for Reproduction, Development and Growth, The University of Hong Kong, Hong Kong, China; 4 The State Key Laboratory of Brain and Cognitive Sciences, The University of Hong Kong, Hong Kong, China; Aarhus University, Denmark

## Abstract

**Background:**

We are moving to second-wave analysis of genome-wide association studies (GWAS), characterized by comprehensive bioinformatical and statistical evaluation of genetic associations. Existing biological knowledge is very valuable for GWAS, which may help improve their detection power particularly for disease susceptibility loci of moderate effect size. However, a challenging question is how to utilize available resources that are very heterogeneous to quantitatively evaluate the statistic significances.

**Methodology/Principal Findings:**

We present a novel knowledge-based weighting framework to boost power of the GWAS and insightfully strengthen their explorative performance for follow-up replication and deep sequencing. Built upon diverse integrated biological knowledge, this framework directly models both the prior functional information and the association significances emerging from GWAS to optimally highlight single nucleotide polymorphisms (SNPs) for subsequent replication. In the theoretical calculation and computer simulation, it shows great potential to achieve extra over 15% power to identify an association signal of moderate strength or to use hundreds of whole-genome subjects fewer to approach similar power. In a case study on late-onset Alzheimer disease (LOAD) for a proof of principle, it highlighted some genes, which showed positive association with LOAD in previous independent studies, and two important LOAD related pathways. These genes and pathways could be originally ignored due to involved SNPs only having moderate association significance.

**Conclusions/Significance:**

With user-friendly implementation in an open-source Java package, this powerful framework will provide an important complementary solution to identify more true susceptibility loci with modest or even small effect size in current GWAS for complex diseases.

## Introduction

Genome-wide association studies (GWAS) have been widely used in the past few years in the community of human genetics [1] and have led to the identification of hundreds of loci affecting risk for complex diseases [2]. By comprehensively examining genetic association across the entire human genome, they could attractively work without any *priori* hypotheses of the disease genes. However, GWAS purely based on the statistical association have also been noted for its limited power to discover predisposing loci or genes with small or modest effect sizes [3], [4]. According to a large GWAS of seven common diseases [5], the associated single nucleotide polymorphisms (SNPs) typically showed odds ratios (ORs) of <1.5. A very large sample size was required to detect these SNPs. Many GWAS are actually underpowered to detect these small or modest effects because of limited sample size [3]. Consequently, current GWAS of most complex traits only have identified a small fraction of trait variance (5 to 10%), leaving much of the heritability of these traits unexplained [6]. If we presume that the unrevealed genetic variants have similar minor allele frequencies and ORs as those identified for type 2 diabetes, more than 800 genetic variants would be required to be able to account for the 40% heritability of a complex disease [6]. Moreover, the existence of genetic heterogeneity of complex diseases further challenges the performance of GWAS. Different individuals may possess different disease risk alleles at different loci in the same gene or in different genes. An individual predisposing variant may well exhibit only weak or modest disease risk in a sample even while it may show large risk in other samples.

Incorporation of the ever-increasing biological knowledge into conventional statistic genetic analysis is becoming a promising strategy to increase the detection power of genetic studies. It has been found that SNPs do have some interesting features relevant to disease risks. The most evident property is their gene features, if available [7]. For instance, SNPs in non-synonymous coding region are expected to have a higher chance to cause a disease than SNPs within the intron [7]. Besides, some non-gene features of SNPs may also provide clue of disease risk. According to recent studies, conservation [8], natural selection [9] and microRNA binding [10], [11] underlie human disease susceptibility. Intuitively, SNPs within regions of strong conservation or strong natural selection or microRNA binding sites are more likely to affect the disease predisposition. Several approaches have been successfully developed to select functionally important SNPs for experimental design of genetic studies for human diseases [12], [13], [14], although inevitably subject to potential knowledge bias.

Recent studies have also found that causative genes for the same (or even phenotypically similar) diseases tend to distribute within the same biological module [15]. The module can be a protein complex [16], a pathway [17], a sub-network of protein-protein interactions (PPIs) [18], or even other similar characteristics like expression patterns [19]. In these shared biological modules, novel underlying disease genes could be predicted from some known disease genes. Based on this rationale, a number of computational tools were made to infer disease genes such as ENDEAVOUR [20], GeneWanderer [21] and CIPHER[22]. Taking advantage of available knowledge as *prior* information, these methods can greatly facilitate genetic mapping of disease genes that have sufficient biological implications.

However, these knowledge-based prioritization methods did not sufficiently utilize characteristics of GWAS. First, they cannot take the GWAS *p-*values into account. Their prioritization is purely base on the biological knowledge. A *p*-value cutoff is often set by genetic investigators to select statistically interesting associations for the knowledge-based prioritization analyses. It is, however, difficult to determine an appropriate threshold for the selection. A too stringent cutoff may run the risk of missing out many true disease susceptibility loci (DSL) with only moderate *p*-values for association while one too loose may introduce too many noises. Second, the disease-gene prediction tools [20], [21], [23] were originally developed for linkage analysis and often neglected genomic features of SNPs. Currently, GWAS use much more genetic markers than genome-wide linkage studies. Some markers (SNPs) themselves have functional implication. For example, an association signal of a SNP at the splicing intron sites of a candidate gene should be given a higher priority than that of a SNP at other intron sites of the same gene. Knowledge-based prioritization analysis sufficiently considering all features of GWAS may lead to a more powerful genetic mapping.

There have been several methods proposed to weight *p-*values for association tests according to prior information. Holm [24] first developed an idea of *p-*value weights. Benjamini and Hochberg investigated the usage of weighting in a variety of settings [25]. Genovese et al. used *p-*value weighting as a frequentist method to add prior knowledge regarding test hypotheses [26]. Roeder et al. developed a weight optimization procedure to avoid the difficulty in selecting appropriate weights for a particular analysis [27]. However, Roeder et al. (2007) had two important limitations for GWAS. First, its statistical exploration of optimal weights ignored the original prior information essentially. Their optimization formula could only ensure the maximization of the average power but could not distinguish the strong-clue SNPs and the weak-clue ones. Therefore, the SNPs in the strong-clue set might be negatively weighted and were less likely to be associated with the disease in question. This violated the original motivation to highlight SNPs with strong functional implications and thus might raise difficulty in interpreting the results. Second, their proposed grouping strategy, although looked flexible, was very abstract. Typically, it is difficult for users to construct proper SNP sets for a given disease in practice because of the heterogeneousness of the diverse information about diseases and genes.

This paper presents a novel bioinformatics and statistical framework to systematically classify, weight, prioritize and interpret association *p-*values from GWAS. It models both the diverse biological knowledge and statistical association *p-*values simultaneously to produce optimal weights for the prioritization. This framework could boost power of current GWAS to identify DSL with small or modest effect size. To test the performance of the framework, we investigated its power by theoretical calculations and empirical simulations, and examined its effectiveness in connecting known associated genes between two databases: the Online Mendelian Inheritance in Man (OMIM) and the Genetic Association Databases (GAD). We then applied this framework to a real case study to highlight SNPs, genes and pathways about late-onset Alzheimer disease (LOAD).

## Materials and Methods

### Data sources

We currently considered eight classes of biological resources in our knowledge-based weighting framework. These diverse genomic resources were integrated into two different datasets, (1) SNP Genomic Features and (2) Gene Functions. These data are updated periodically by our data-server program. More resources will be added into the two datasets in the feature.

#### SNP Genomic Features dataset

The SNP information dataset is made from four different resources. The major SNP data were downloaded from the dbSNP database of NCBI (ftp://ftp.ncbi.nih.gov/snp/organisms/human_9606/ASN1_flat/). The software currently uses Build 130 (May 03, 2009), which includes 17,804,036 Homo sapiens SNPs (7,344,853 within genes). The second resource is the conservation score information from the UCSC Genome Browser website (http://hgdownload.cse.ucsc.edu/). The conservation scores were generated based on sequence alignments of 16 vertebrate genomes with the human genome. The third resource is the positive selection score information of Phase 1 and Phase 2 SNPs in the HapMap Project, downloaded from an analyzed dataset (http://haplotter.uchicago.edu/selection/) [28]. The last one is the human microRNA target gene binding site information from Sanger's miRBase (http://microrna.sanger.ac.uk/). We used version 5.0, containing more than 879 thousand target binding sites.

#### Gene Function dataset

The gene function dataset consist of four kinds of gene information: (1) OMIM disease information, (2) tissue specific-expression, (3) biological pathways, and (4) PPIs. The OMIM's [29] Morbid Map (MM) information (ftp://ftp.ncbi.nih.gov/repository/OMIM/morbidmap), a compiled dataset of human genetic disorders and responsible genes (containing 5,413 entries as of Feb. 22, 2010), was integrated to facilitate defining seed candidate genes of given diseases. The tissue-specific expression genes were download from an analyzed dataset of mRNA expression arrays by Greco et al., where 1601 genes were specifically express on 78 different human tissues [30]. Two biological pathway databases were considered, KEGG (http://www.genome.ad.jp/kegg/pathway.html) and BioCarta (http://www.biocarta.com/). We collected and compiled 13,680 and 5,390 pathway-gene entries from the KEGG and BioCarta databases, respectively (as of Dec. 11 2009). The PPI entries were integrated from five databases: Human Protein Reference Database (DPRD, http://www.hprd.org/) [31], Interologous Interaction Database (I2D, http://ophid.utoronto.ca/i2d) [32], Biomolecular Object Network Databank (BOND) [33], Molecular INTeraction database (MINT, http://mint.bio.uniroma2.it/mint/) [34] and General Repository Interaction Datasets (BioGRID, http://www.thebiogrid.org/). The protein IDs in these databases were mapped onto their genes symbols by our program. The total number of unique pair-wise interactions between genes was 100,268 (as of Dec. 11, 2009).

### Construction of a bioinformatics and statistical integration framework

**Figure 1:**
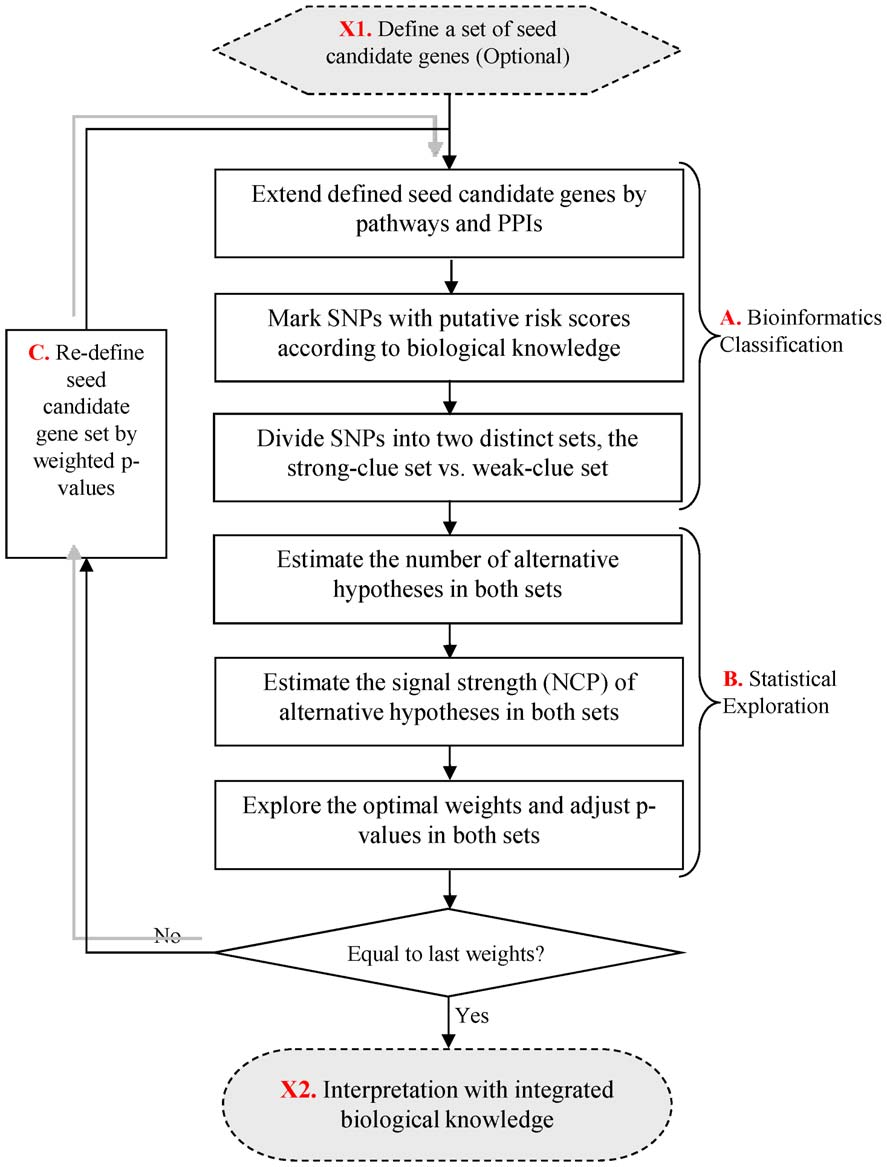
Flow diagram showing the weighting procedure of the bioinformatics and statistical integration framework. In the figure, Step A, B and C constitute the kernel part of the procedure. They run iteratively. In Step A, SNPs are classified into two distinct sets (a strong-clue set and a weak-clue set) based on biological knowledge integrated from various bioinformatics databases. In Step B, a statistical exploration is conducted to adjust *p-*values of SNPs by optimal weights that are in favor of the strong clues. In Step C, the top-*n* (say, Top-10) genes according to the weighted *p-*values are selected to form a new set of seed candidate genes. The iteration stops when the weights in the current iteration are equal to the ones in the last iteration. X1 and X2 are auxiliary steps. In Step X1, one can define a set of important seed candidate genes for the disease in question. However, this step is optional. If there is no pre-defined seed candidate genes, the top-*n* (say, top-20) genes according to the original association *p-*values are picked up to form a new set of seed candidate genes. In Step X2, biological knowledge of the highlighted SNPs can be specifically retrieved to interpret the association significances under the framework.

We constructed a framework to integrate these biological resources and weight SNPs' association *p*-values from GWAS. The kernel of integration framework is the weighting procedure as shown in Figure 1. This procedure includes two main parts, A) Bioinformatics Classification and B) Statistical Exploration. In the part of Bioinformatics Classification, all SNPs are classified into two distinct sets (the strong- and the weak-clue sets) based on biological knowledge such as SNPs, genes, microRNA binding, pathways and PPIs, integrated from various bioinformatics databases. SNPs in the strong-clue set are assumed to have higher disease risk than those in the weak-clue set. In the Statistical Exploration part, a statistical approach is developed to produce optimal weights for SNPs by modeling the risk set statuses (as prior information) and association *p-*values of SNPs simultaneously. The optimal weights here are defined as the weights which can 1) maximize the average power of all tests on the whole genome while controlling the family-wise error, and 2) highlight SNPs in the strong-clue set.

The two parts run iteratively via an intermediate step, “Re-defining seed candidate genes” (indicated as Step C in Figure 1). The top-*n* (say, top-20) genes according to the newly weighted *p-*values are chosen to form a new seed-gene set, which are used to re-group the SNPs into two risk sets and then re-generate the weights. The iteration does not stop until the weights converge eventually. In addition, there are two auxiliary steps, (1) pre-defining seed candidate genes, and (2) biological interpretation, denoted as X1 and X2 in Figure 1 respectively. In Step X1, one can define a set of initial seed-candidate genes, which are probably confirmed by many previous independent genetic studies and/or molecular functional studies for the disease in question. However, this step is optional. If there are no pre-defined seed candidate genes, the top-*n* genes according to the original association *p-*values are selected to form a set of seed genes. In Step X2, biological knowledge of the highlighted SNPs can be specifically retrieved to interpret the association significances. The framework has been implemented in an open-source Java package named “A systematic biological Knowledge-based mining system for Genome-wide Genetic studies” (KGG, http://bioinfo.hku.hk/kggweb/). In addition, KGG can find additional SNPs of the HapMap dataset in strong linkage disequilibrium (LD) (say, r2>0.9) with the SNPs in the local GWAS dataset. If the maximal risk score among the newly added HapMap SNPs is larger than that of the local SNPs, the former will be assigned to the local SNPs. This is a simple strategy to access some missing functional SNPs using the typed tag-SNPs. Preferably, one can perform weighting analysis for GWAS association results which have been expanded by genotype imputation.

#### Bioinformatics Classification

The seed candidate gene set is used to introduce more candidate genes via an extension protocol. The extended candidate gene set includes genes sharing the same biological pathways with the seed genes, according to the Kyoto Encyclopedia of Genes and Genomes (KEGG, http://www.genome.ad.jp/kegg/pathway.html) and BioCarta (http://www.biocarta.com/) pathway databases. In addition, the extended set includes genes whose proteins interact with the proteins coded by the seed genes. The underlying assumption is that genes responsible for the same (or even phenotypically similar) diseases are more likely to distribute within the same pathways or sub-networks of PPIs [17], [18].

The SNPs to be prioritized are then assigned putative disease risk scores based on whether they are in the extended candidate gene set and other genomic information of SNPs themselves via a three-step scoring protocol. The protocol is detailed in Table S1. First, SNPs are given preliminary risk scores according to their gene features since SNPs of different gene features may have different disease risk [7]. Second, these risk scores are further adjusted according to three non-gene features, (1) conservation scores, (2) positive selection scores, and (3) microRNA binding status. SNPs with high conservation scores, high positive selection scores, or within microRNAs' target binding sites are assumed to have higher disease risk [8], [9], [10], [11]. Finally, SNPs belonging to candidate genes are given 3 more points.

After the assignment of the risk scores, a risk score cutoff, four, is used to divide SNPs into two distinct sets, the strong- and the weak-clue sets. SNPs with risk scores equal to or over four belong to the strong-clue set and the remainders are put into the weak-clue set. According to the scoring protocol, once a SNP is within the 2 kilo-base pairs (Kb) 5′ or 500 base pairs (bp) 3′ of a candidate gene, it will have a score at least equal to four. That is, this cutoff can ensure all SNPs of interested candidate genes to be classified into the strong-clue set, which might be favorably weighted. Meanwhile, the classification procedure implies that the framework will never highlight SNPs far way from genes unless the SNPs have at least two promising non-gene properties: high conservation scores, high positive selection scores, or being within microRNAs' target binding sites.

#### Statistical Exploration

Assume there are *m_S_ and m_W_* SNPs in the strong- and weak-clue sets. Their test *p*-values in a genome-wide association study are 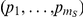 and 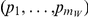 respectively. These *p*-values correspond to standardized test statistics 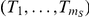 and 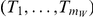. In the strong-clue set, there are *m_0,S_* and *m_1,S_* SNPs following the null and alternative hypotheses respectively. The proportion of null hypotheses is 
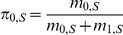
.The test statistics of null hypotheses are approximately under 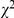 distribution with 1 degree of freedom (d.f.). The test statistics of alternative hypotheses are approximately 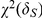 distributed with 1 d.f. and noncentrality parameter (NCP) *δ_S_.* Here we simply assume that all alternative hypotheses in the strong-clue set are independent and under the identical 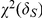 distribution. In the present study, the NCP is also called signal strength. Similarly, in the weak-clue set, there are *m_0,W_* and *m_1,W_* SNPs following null and alternative hypotheses respectively. The proportion of null hypotheses is 
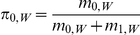
. The test statistics of alternative hypotheses are approximately 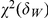 distributed with 1 d.f. and NCP *δ_W_*.

The method of Storey and Tibshirani [35] after slight modification was used to estimate the proportion of null hypotheses in the strong and weak clue sets, 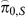 and 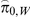. The number of alternative hypotheses in both sets are approximated as 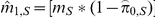 and 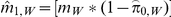, respectively. We then extended a method of Li and Yu (2009), the moment estimate for truncated non-central chi-squared distribution, to infer NCPs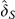 and 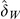 in the two different SNP sets [36] (Please read the Methods section of Supporting Information S1 for details).

Once the number and NCP of alternative hypotheses in both SNP sets are obtained, we can start to explore the optimal weights. Intuitively, the statistical exploration attempts to find proper weights which maximize the number of significant SNPs while controlling the overall false positive rate on the whole genome by up-weighting SNPs following alternative hypotheses in strong-clue set and down-weighting SNPs following alternative hypotheses in the weak -clue set. Denote the weights for the strong- and weak-clue sets by *w_S_* and *w_W_* respectively. Given a *p-*value rejection threshold *α*, the power of a single weighted test in the strong-clue set is 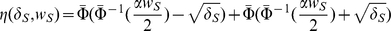 [27], where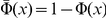 is the complement of the standard normal cumulative distribution function. Analogously, the power of a single weighted test in the weak-clue set is 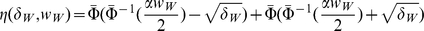. As we have *m*_1_ = *m*_1,*S*_ + *m*_1,*W*_ alternatively hypotheses in total, the average power in the whole genome is 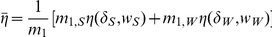. An algorithm was developed to explore the optimal *w_S_* and *w_W_* which can maximize the average power, 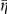, while 1) constraining 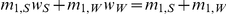 to control the family-wise error and 2) constraining *w_S_*≥*w_W_* to highlight SNPs in the strong-clue set by favorable weights. The weights are used to adjust association *p-*values of SNPs; a weighted *p-*value is equal to the original one divided by the weight. These weighted *p-*values are valid for multiple-comparison methods like the standard Bonferroni and false discovery rate (FDR) corrections [37]. Details of the Statistical Exploration are described in the Methods section of Supporting Information S1. From the frequentist viewpoint, a weighted p-value may be no longer a standard p-value at least for a single test. As it is an adjusted *p*-value given a *prior* weight, to some extent, it can be regarded as “the Bayesian posterior *p*-value”. If we borrow similar idea in Storey (2003), it may be more sensible to name a weighted *p*-value as “*q*-value” which was originally introduced to interpret the positive false discovery rate [38].

### Computer simulations

In genetic association studies, the association p-value of alternative hypotheses is usually affected by two important factors, effect size (i.e. genetic relative risk) of DSL and sample size. Thus, we used simple computer simulation (which assumes the DSL have been assigned into the strong-clue set) to basically look into how they affect the performance of our weighting approach. The LOAD was supposed as our target disease in the simulation. Three genes (*GAPDHS*, *PRNP* and *ACE*) were randomly selected from a LOAD gene set proposed by Bertram et al. as susceptibility genes of the simulated disease [4]. Each gene is assumed to have one LOAD predisposing SNP. The three SNPs (rs11882238 and rs12625444 and rs4351) have different minor allele frequencies (0.0750, 0.2167 and 0.4167). We simulated genotypes and phenotypes to investigate the power and false positives of our weighting approaches. Detailed methods of the simulation are described in the Methods section of Supporting Information S1.

### Candidate gene extension and testing

Although it is impossible to completely validate the candidate-gene extension protocol (the fist step of the weighting procedure as indicated in Figure 1), a conceptual verification by available datasets is still feasible. In the present study, we collected genes as seed candidates for each disease in the OMIM database. Then we expanded the seed candidate gene set by our protocol for each disease. In the expanded gene set, we counted genes which had positive association for the same disease reported by previous studies in the GAD. The coverage percentage for a disease was defined as the proportion of these genes positively reported in the GAD among the expanded candidate gene set. In the OMIM's MM file, out of 5,183 MM entries, 3,897 entries with the “(3)” tag (indicating that at least one mutation in the particular gene was causative to the disorder) were selected for the validation. In the GAD on March 10, 2009, there were 11,571 (out of 39,910) items with positive association annotation. Diseases having less than three seed candidate genes were excluded. Consequently, 108 unique diseases were left eventually. A *p-*value was calculated by the cumulative hyper-geometric distribution to evaluate the significance of the coverage:
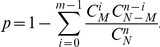
where *N* is the number of all known human genes and *M* is the number of genes positively reported to be associated with the given disease in the GAD. The *n* is the number of expanded genes based on the seed candidate genes for a disease and *m* is the number of genes in the expanded candidate gene set and positively reported in GAD as well.

### Application to a real LOAD dataset

#### The LOAD dataset

We downloaded a LOAD dataset from the TGEN database (http://www.tgen.org/research/index.cfm?pageid=1065, Translational Genomics Research Institute; TGEN). It contained 961 histopathologically verified Caucasian LOAD cases and 550 age-matched controls, which were collected from three cohorts, “neuropathological discovery cohort”, “neuropathological replication cohort” and “clinical replication cohort”. These subjects were at least 65 years old at the time of their death or last clinical assessment. The Affymetrix 500K GeneChip (Affymetrix, Santa Clara, CA) was used to survey 502,267 SNPs in each subject. Genotypes were called by SNiPer-HD [39] and BRLMM (Affymetrix) software. Additional description of the dataset can be found in Reiman et al. (2007).

#### Knowledge-based weighting analysis

After producing allelic association *p-*values using PLINK [40], we used KGG to conduct the knowledge-based weighting analysis to highlight SNPs and genes which might be promising for replication. We forced KGG to choose the top 20 genes according to SNPs p-values as seed genes. The seed gene set was expended by including genes having two-level PPI and sharing the same pathways with the seed genes. SNPs in the dataset were classified into the strong- and weak-clue sets according to their gene features, the conservation (default threshold 0.8) and selection scores (default threshold 2.0 according to the HapMap CEU population), miRNA binding site information, and the expanded candidate gene set. The weighting procedure was allowed to iterate until the optimal weights converged. In the iteration, the top-20 genes according to the weighted association *p-*values were picked up to form a new set of seed genes. Pathways containing over 300 or less than 2 genes were excluded. The FDR method of Benjamini and Hochberg (1995) was used for multiple-testing correction with an error rate 5%.

#### Significance of pathway enrichment

The significance of pathway enrichment is also measured by a *p-*value according to the cumulative hyper-geometric distribution.

## Results

### Theoretical power gain and power loss

Figure S6 shows the theoretically increased power (or power gain) and decreased power (or power loss) for detecting a true alternative hypothesis in the two sets (Detailed methods of the simulation are described in the Methods section of Supporting Information S1). The power gain is related to the signal strengths or NCPs of the alternative hypotheses in both sets. As shown in Figure S6(a), a large power gain (over 10%) can occur only when the signal strength is approximately within the region [4], [6]. This result implies that our weighting method is more effective for alternative hypothesis with midsize signal strength. Therefore, in the implementation of the weighting method, we excluded the p-values which have been already statistically significant. On the contrary, the power loss, nonetheless, is generally very small regardless of the signal strengths. As shown in Figure S6(b), the largest power loss for an alternative hypothesis in the weak-clue set is only 0.4%, which corresponds to the power gain 17% in the strong-clue set. The large difference between the amount of power gain and power loss implies the worthwhile trying of our weighting method.

### Computer simulation results

**Figure 2:**
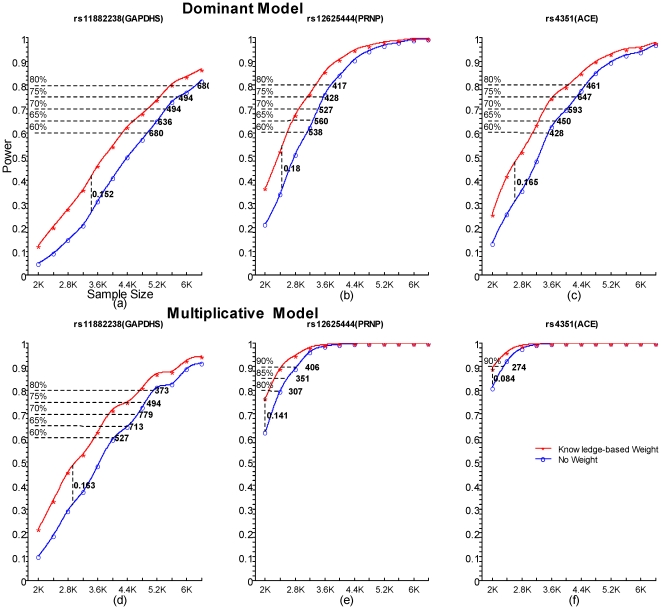
Comparison of power between the weighted and non-weighted basic allelic association tests in the simulated dataset when sample size varies. Plots a), b) and c) show the power identifying rs11882238, rs12625444 and rs4351 under the dominant model, respectively. Meanwhile, Plots d), e) and f) represent the power under multiplicative mode for the three SNPs. All of the three SNPs are assumed to be in the strong-clue set. The sample size increases from 2000 to 6400 by 400 with equal number of cases and controls. On the plots 1K denotes 1000 subjects. The curves are smoothed by the natural cubic spline method. The maximal power difference between these curves is labeled by a dashed vertical line on each plot. The dashed horizontal line indicates the saved whole-genome sample size by our knowledge-based weight approach for a given power compared with the original statistical test.

The Figure S3, Tables S3 and S4 show that the power gain of the weighting approach varies with the relative genetic risks. When the genetic risk leads to midsize signal strength, the power gain can be over 15% for SNPs in the strong-clue set. However, when the effect size is too small or too large, the power gain becomes small, compared with the original statistical test. When the susceptible SNP is in the weak-clue set, our weighting framework almost has the same power as the original test (only shown in Tables S3 and S4). Figure 2 shows the relationship between power gain and sample size. The pattern is quite similar to that in Figure S3. When the sample size corresponds to moderate signal strength, the power gain can be very large (again over 15%). If the sample size is very large, the original method has already had high power (say, over 90%) to identify the SNPs and so there is little room for any improvement. In addition, Figure 2 also indicates that the weighting approach may save hundreds of whole-genome subjects to achieve an acceptable power in practice, say 80%, compared with original statistical methods.

All these results observed in the simulation coincide with the theoretical calculation above except that the weighted method detects slightly more false positive discoveries (Shown in the Tables S3 and S4). This cannot be explained by our statistical model because the family-wise error has already been controlled theoretically. The most likely reason is the dependence of SNPs due to linkage disequilibrium between neighborhood SNPs, which is difficult to be modeled theoretically. At any rate, the inflated false positive rate is quite small and may merely result in minor loss of cost compared to the benefit from the power gain. For example, The Monte Calor mean of false positive number is 0.78( = 1.92–1.14) at genetic risk 1.55 (in the Tables S3) among all tested 28370 SNPs [2.75E-5( = 0.78/28370) per test] while the power to identify rs11882238 can be 17.1% ( = 59.9%−42.8%) higher than the original test. Therefore, it may be acceptable to tolerate slightly more false associations to gain one or several true associations, similar to the reason of the application of FDR approach [41].

### Effectiveness of candidate gene extension by OMIM and GAD

Figure S4 and Table S5 show how many genes in the GAD can be derived from the OMIM genes through our candidate gene extension protocol. In the histogram of the coverage for the 73 diseases (Figure S4), 68(93.2%) diseases have the coverage ≥ 50%. It is 80% or so for many common complex diseases such as Diabetes Mellitus, Obesity, Alzheimer's disease (AD) and Parkinson's disease. These results imply the effectiveness of our weighting framework for many human complex diseases. Once the OMIM genes are utilized as seed candidate genes, most promising genes in the GAD will be deduced and SNPs these genes might be highlighted by our weighting procedure.

### Knowledge-based analysis in a LOAD dataset: a case study

We applied this weighting framework to prioritize and interpret associations in a published real LOAD dataset for a genome-wide case-control study [42]. This dataset included 307448 SNPs passing quality control criteria. Each SNP had an allelic association *p-*value, which was generated by Plink [40]. The genomic inflation factor for the 307448 *p-*values (based on median chi-squared) was 1.07125, indicating a slight inflation of moderate association significance in the dataset. Figure S1 shows the Q-Q plot of the *p-*values. We defined these *p-*values as the “original *p-*values” and inputted them into KGG for the knowledge-based weighting analysis. According to the original *p-*values, there was only one significantly associated SNP, rs4420638 (p = 3.6E-36), Benjamini and Hochberg (1995) test with FDR 0.05.

SNPs were marked with risk scores and separated into the strong- and weak-clue sets based on the integrated knowledge in our dataset. Two optimal weights for the strong- and weak-clue sets, 7.77 and 0.001, were ultimately obtained by the default settings on KGG. In the strong-clue set there were 1194 (2.78%) SNPs were assigned the high weights and 8088 (3.03%) SNPs in the weak-clue set were given the low weight. There were 308 SNPs with the weighted p-values ≤ 5.0E-4 (listed in the Table S6).

**Figure 3:**
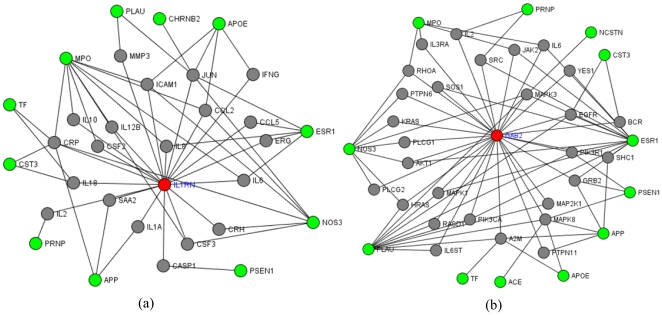
Three 2-level PPI sub-network enriched by *IL1RN, GAB2* and important candidate genes. This figure is plotted by our tool KGG. Each node denotes a gene labeled by “Gene Symbol”. The edge indicates a PPI between proteins encoded by two genes. The red and green nodes denote the tested genes with significant SNPs and important candidate genes respectively. Here the “2-level” means that the minimal length from a tested gene to an important candidate gene is 2 (edges). There are intermediate genes (in gray on the plot) between the tested gene and important candidate genes, which have PPI with the both.

We did literature survey for genes containing weighted p-values (or *q*-values) ≤5.0E-4 at their highlighted SNPs, listed in the Table S6. Because few SNPs overlapped across datasets of various studies, we limited survey to involved genes, which were positively reported to be associated with LOAD at least once in GAD, Alzforum database (http://www.alzforum.org/res/com/gen/alzgene/default.asp), and in the NCBI PubMed. Fourteen genes (except for the extremely significant gene APOC1, a gene 5 kb away from *APOE*) were suggested as susceptibility genes by at least one previous independent study (Detailed in Table S2) among 188 genes with up-weighted SNPs p-values ≤5.0E-4. Among the 14 genes, two genes, *IL1RN* and *GAB2*, have more than 3 independent studies suggesting their susceptibility to LOAD. *IL1RN* encodes proteins inhibiting the activities of interleukin 1, alpha (*IL1A*) and interleukin 1, beta (*IL1B*), which is related to immune and inflammatory responses. There are growing evidences supporting that inflammatory processes most certainly play an important role in the pathogenesis of AD [43]. In the annotation analysis on KGG, we found this gene had a 2-level indirect PPI with 11 important candidate genes (Figure 3 (a)). *GAB2* is a member of the growth factor receptor–bound protein 2(*GRB2*)-associated binding protein (GAB) gene family. *GRB2* has been reported to bind tau, amyloid-β precursor protein (*APP*), and *PSEN1* and *PSEN2*. These interactions have been advised to regulate signal transduction and to be involved in the pathogenesis of AD [44], [45]. In our PPI dataset, it has indirect PPIs with 12 important candidate genes including the PPIs 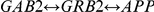 (Figure 3 (b)). We also tried similar literature survey for the original association *p-*values. There are 294 SNPs (belonging to 112 different genes) whose original association p-values are ≤ 5.0E-4. In the GAD, Alzforum database, and PubMed, there was only one gene, COL11A1, among the 112 genes supported by only one genetic association study of LOAD [46], except for the extremely significant gene *APOC1*. The weighting method largely enriched previously reported genes to be 4.47:1 ( = 15/188:2:112).

Apart from the association consistent with previously findings at the gene level, there are also two interesting pathways enriched by both the genes with highlighted SNP and some very important candidate genes of AD (Figure S5). The first one is the KEGG AD pathway. Seven genes (*COX7B2*, *SNCA*, *GRIN2A, SDHA*, *PPP3CA*, *CACNA1C* **and** *CACNA1D*) with the highlighted SNPs in the Table S6 and six important candidate genes clustered within this pathway (shown in the Figure S2). The other interesting pathway, although not so obvious as the AD pathway, is the Calcium signaling pathway. Twelve genes (*EGFR*, *GNA14*, *PDE1A*, *EDNRA*, *GNAL*, *TRPC1*, *ADCY2*, *GRIN2A*, *RYR2*, *CACNA1C, PPP3CA* **and** *CACNA1D*) with highlighted SNPs and one important candidate gene (NOS3) are enriched in this pathway. Given the very small *p-*values, there is every reason to suspect that certain unraveled functional implications of the LOAD underlay these significant enrichments. Actually, association between the Calcium signaling pathway and the AD has been proposed by many molecular genetic and genetic epidemiological studies [47], [48], which supports the intraneuronal calcium dysregulation hypothesis of AD [49].

While these genes with previous supporting and enriched pathways provide a “proof of principle”, other genes with highlighted SNPs, although their function for LOAD has not been well studied, are also of interests. For instance, suggestive association significance (according to the weighted *p-*value) occurs at the missense polymorphism (rs7817227) of *C8orf80*. These SNPs and genes should also be given higher priority in the following-up replications.

## Discussion

We have presented a novel bioinformatics and statistical framework to prioritize SNPs of GWAS. Unlike previous bioinformatics disease-loci prediction approaches, this weighting method in our framework directly modeled both biological knowledge and statistic association significances emerging from GWAS to produce optimal weights for the prioritization. It could properly up-regulate the moderate *p-*values for SNPs but with strong functional clues. This framework has a potential to largely improve the power of current GWAS to identify more DSL, particularly those with modest effect size. In addition, the integrated biological context also helps on interpreting the observed association and thus speculating the genetic and pathogenic mechanisms of a disease.

We conducted a series of investigations to examine the effectiveness of this framework. In the theoretical calculation and computer simulation, it had great potential to achieve extra over 15% power to identify an association signal of midsize strength. According to the empirical simulation results (Figure 2), the weighting approach might save hundreds of whole-genome samples to get the same acceptable power, say 80%, compared to the original association test. In a validation test, its candidate gene extension protocol had a very good performance to cover previously reported genes for the most common diseases in GAD. In the application to a LOAD dataset for the purpose of a proof of principle, it highlighted some genes that were suggested as susceptibility genes of LOAD by previous independent genetic studies and two important AD related pathways. Taken together, this framework provided a worthwhile alternative to strengthen the explorative performance of the GWAS. It may be particularly useful for the prioritization of SNPs for follow-up replications at the first stage of the multistage GWAS design [50] and for deep sequencing studies. The whole weighting procedure and other assistant functions like tracking and visualization of the biological knowledge have been built in a user-friendly open-source tool named KGG (http://bioinfo.hku.hk/kggweb/).

In the literature survey, we included all genes for which at least one association study (either family or case-control studies) concluded the positive association. Admittedly, given the fact that conflicting findings in genetic studies of complex diseases occur commonly regardless of study design, there are also negative reports for the genes we showed in the Table S2, which are not listed in the present paper. However, we assumed that both negative and positive association studies in various populations might be correct with the underlying reason of genetic heterogeneity in different populations, and possible gene-gene and gene-environment interactions [51], [52]. Also due to this reason, an individual GWAS in a specific population cannot present association signals at all possible susceptibility genes. That may be the reason why even the 12 genes proposed by meta-analysis [4] were not highlighted ultimately in our case study as well. Probably, this dataset do not contain association signal at these genes. In addition, we also compare our results with one of the latest GWAS published in *Nature Genetics* [53]. In the Table S2 of this paper, showing association *p-*values <1.0E-3, 32 genes were highlighted by our weighting procedure. In their table there were 219 genes having registry in our LOAD GWAS dataset which includes 15300 genes in total. Our weighting procedure highlighted 555 genes in total. The probability of highlighting the 32 genes and more by chance is very small, 1.64E-11 (cumulative hyper-geometric distribution).

It should be noted that we did not use any well-known candidate genes of LOAD as the initial seed genes to train the weighting framework. Otherwise, it may be subjected to the criticism of circular logic because these important candidate genes tend to be studied more by previous candidate-gene studies and are more likely to be selected in our literature survey. However, the important candidate genes could be used to introduce other novel but functionally related genes based-on this general foundation. Statistically, the important candidate genes are regarded as prior information. Taking into account the prior information, our method will re-evaluate the association in the local dataset, which is a Bayes-like idea. Therefore, in real analyses of GWAS, we suggest using some important candidate genes (if available) as initial seed genes to generate hypothesis for replication. If the important candidate genes have suggestively significant SNP p-values, it may well be highlighted by our weighting procedure and need to be replicated using new data. However, our weighting procedure may also spotlight other interesting genes which have functional correlation with the important candidate genes and suggestively significant association p-values.

Our integrated dataset has obvious partiality for SNPs within genes at present. Because most available biological resources are biased toward genes, SNPs pertaining to known genes could have much more relevant prior information. Consequently, the resulting weights may be more effective for associated SNPs belonging or close to known genes. Actually, there is a trend of gene-centricity among available GWAS findings. According to a recent survey of 118 GWAS articles, 68% of reported SNPs with disease association lie within 60 Kb of a RefSeq gene [54]. This gene-centricity trend may imply that the susceptibility loci within and around genes are really dominant although not all. Therefore, methods that focus more on gene regions could still be productive regardless of their intrinsic bias. Moreover, for complex diseases, functionally validating association hits far from gene region remains to be an intractable challenge up to now. Setting out from the relatively easier points is a feasible strategy. In any case, we have begun to partly address this issue by considering three kinds of non-gene information, conservation score, selection score, and miRNA binding. More information will be added in the future. In fact, knowledge bias is a common and intrinsic limitation of all knowledge-based analysis methods e.g., [12], [20], [21], [22], [55], [56]. As the limitation seemed not to prohibit the success of these methods in applications, it is unlikely that it will significantly confine the application of our framework.

## Supporting Information

Figure S1 (0.04 MB DOC)

Figure S2 (0.09 MB DOC)

Figure S3 (0.05 MB DOC)

Figure S4 (0.03 MB DOC)

Figure S5 (0.27 MB DOC)

Figure S6 (0.09 MB DOC)

Supporting Information S1 (0.27 MB DOC)

Table S1 (0.04 MB DOC)

Table S3 (0.11 MB DOC)

Table S2 (0.11 MB DOC)

Table S4 (0.11 MB DOC)

Table S5 (0.24 MB DOC)

Table S6 (0.75 MB DOC)
