# Supplementary material for: A Knowledge-Based Weighting Framework to Boost the Power of Genome-Wide Association Studies
Source: PLoS One. 2010 Dec 31;5(12):e14480. doi: 10.1371/journal.pone.0014480 (PMC3013112; doi:10.1371/journal.pone.0014480)

Figure S1: Q-Q plot of the original p-values.

The green line is a reference which assumes that all observed p-values are under the null hypotheses. The red open triangle denotes the observed p-value which is far from the expected.


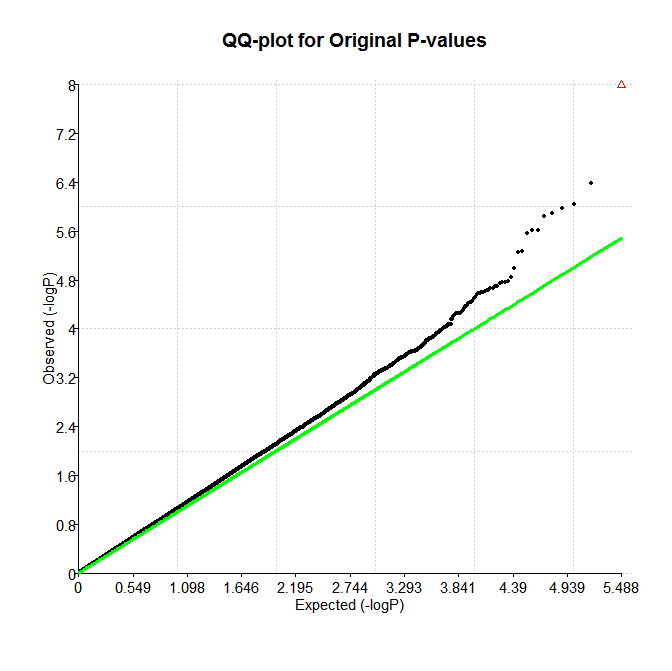

Supplement: Figure S1 — (0.04 MB DOC) [file pone.0014480.s001.doc]
