# Supplementary material for: A Knowledge-Based Weighting Framework to Boost the Power of Genome-Wide Association Studies
Source: PLoS One. 2010 Dec 31;5(12):e14480. doi: 10.1371/journal.pone.0014480 (PMC3013112; doi:10.1371/journal.pone.0014480)
Supplement: Figure S2 — (0.09 MB DOC) [file pone.0014480.s002.doc]

Figure S2: Alzheimer’s disease pathway in the KEGG database enriched by the tested genes and important candidate genes.

The five tested genes, *COX7B2*, *GRIN2A,* *CACNA1C,* *SDHA,* and *PPP3CA*, are included in CxIV, NMDAR, VDCC, CxII and Cn respectively. The eight important candidate genes are indicated by red box on the plot. Our tool KGG can automatically generate codes to marker these genes.
