# Supplementary material for: A Knowledge-Based Weighting Framework to Boost the Power of Genome-Wide Association Studies
Source: PLoS One. 2010 Dec 31;5(12):e14480. doi: 10.1371/journal.pone.0014480 (PMC3013112; doi:10.1371/journal.pone.0014480)
Supplement: Figure S3 — (0.05 MB DOC) [file pone.0014480.s003.doc]

Figure S3: Comparison of power between the weighted and non-weighted basic allelic association tests in the simulated dataset when genetic risk varies.

Plots a), b) and c) show the power identifying rs11882238, rs12625444 and rs4351 under the dominant model respectively. Meanwhile, Plots d), e) and f) represent the power under multiplicative mode for the three SNPs. All of the three SNPs are assumed to be in the strong-clue set. The genetic risk of the heterozygous genotype of each SNP increases from 1.1 to 1.65 by 0.05. The curves are smoothed by the natural cubic spline method. The maximal power difference between these curves is labeled by a dashed vertical line on each plot.
