# Supplementary material for: A Knowledge-Based Weighting Framework to Boost the Power of Genome-Wide Association Studies
Source: PLoS One. 2010 Dec 31;5(12):e14480. doi: 10.1371/journal.pone.0014480 (PMC3013112; doi:10.1371/journal.pone.0014480)
Supplement: Figure S4 — (0.03 MB DOC) [file pone.0014480.s004.doc]

Figure S4: Histogram of the coverage percentage of the candidate gene extension for human diseases.

There are totally 73 diseases selected from OMIM and GAD, whose coverage *p-*values are less than 0.05.
