# Supplementary material for: A Knowledge-Based Weighting Framework to Boost the Power of Genome-Wide Association Studies
Source: PLoS One. 2010 Dec 31;5(12):e14480. doi: 10.1371/journal.pone.0014480 (PMC3013112; doi:10.1371/journal.pone.0014480)
Supplement: Figure S5 — (0.27 MB DOC) [file pone.0014480.s005.doc]

Figure 5S: Important candidate genes and tested genes with highlighted SNPs in AD pathway and Calcium signaling pathway of KEGG database.


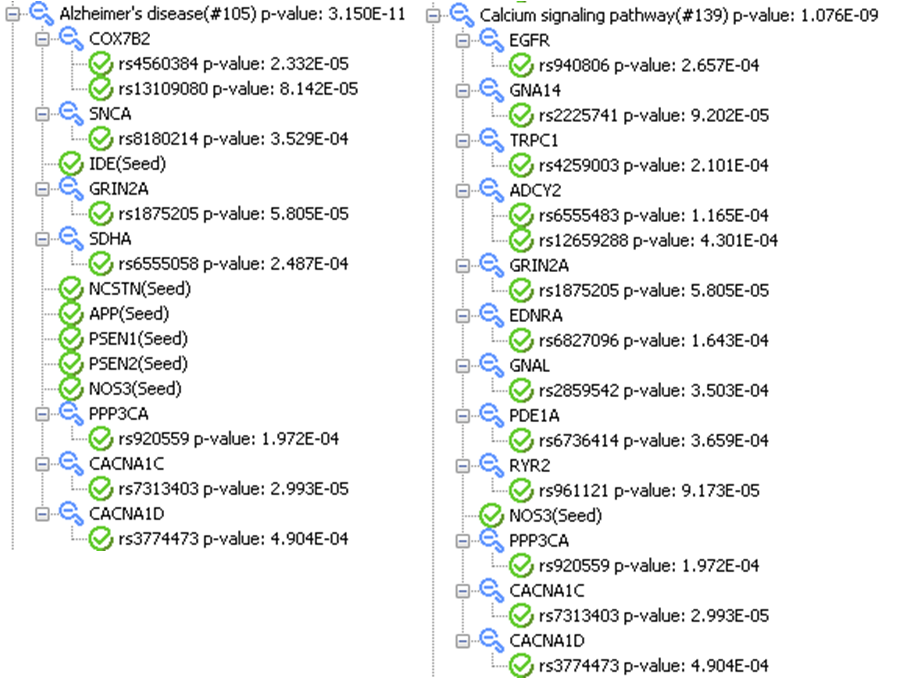


This tree is draw by KGG. The *p-*values following the pathway name indicate the enrichment significances.
