# Supplementary material for: A Knowledge-Based Weighting Framework to Boost the Power of Genome-Wide Association Studies
Source: PLoS One. 2010 Dec 31;5(12):e14480. doi: 10.1371/journal.pone.0014480 (PMC3013112; doi:10.1371/journal.pone.0014480)
Supplement: Figure S6 — (0.09 MB DOC) [file pone.0014480.s006.doc]

Figure 6S: Theoretical power gain and loss of an alternative hypothesis in the strong- and weak-clue sets.


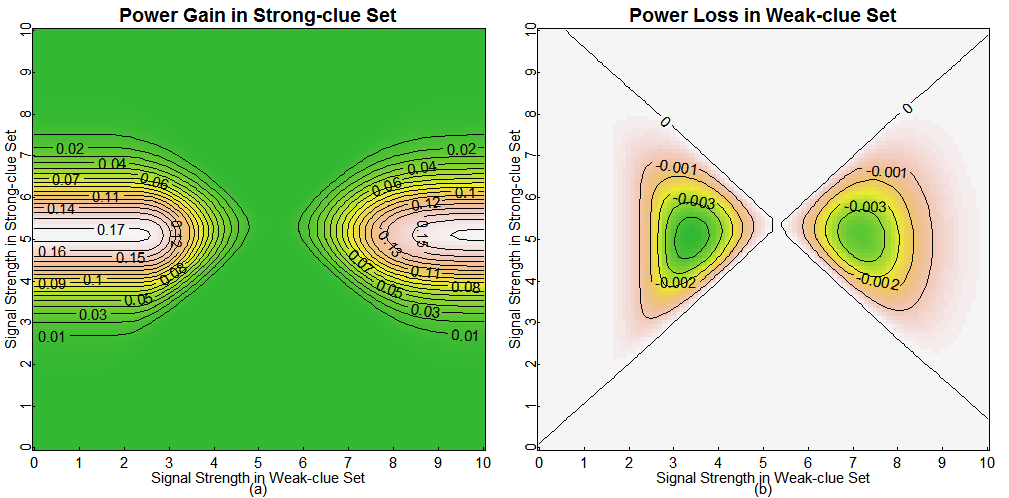


The signal strength indicates the noncentrality parameter *δ* of the non-central chi-square distribution with 1 degree of freedom. The assumed numbers of alternative hypotheses in the strong- and weak-clue sets are 10 and 90. The cutoff *p-*value to reject a null hypothesis is 1.0e-7.
