# Supplementary material for: A Knowledge-Based Weighting Framework to Boost the Power of Genome-Wide Association Studies
Source: PLoS One. 2010 Dec 31;5(12):e14480. doi: 10.1371/journal.pone.0014480 (PMC3013112; doi:10.1371/journal.pone.0014480)
Supplement: Supporting Information S1 — (0.27 MB DOC) [file pone.0014480.s007.doc]

**Supporting Information S1 of**

A knowledge-based weighting framework to boost the power of genome-wide association studies

Miao-Xin Li^1,2,3^, Pak C. Sham^2,3,4^, Stacey S. Cherny^2,4^, You-Qiang Song^1,3,*^

^1^Department of Biochemistry, ^2^Department of Psychiatry, ^3^The Centre for Reproduction, Development and Growth, ^4^The State Key Laboratory of Brain and Cognitive Sciences, The University of Hong Kong, Pokfulam, Hong Kong SAR, China

**Content**

[**Methods** 2](#__RefHeading___Toc275420436)

[**1. Statistical exploration of optimal weights in the strong- and weak-clue sets** 2](#__RefHeading___Toc275420437)

[***1.1. Estimate number of alternative hypotheses*** 2](#__RefHeading___Toc275420438)

[***1.2. Estimate signal strength (NCP) of alternative hypotheses*** 2](#__RefHeading___Toc275420439)

[***1.3. Produce optimal weights*** 3](#__RefHeading___Toc275420440)

[**2. Theoretical calculation of power gain and power loss** 4](#__RefHeading___Toc275420441)

[**3. Computer simulation** 5](#__RefHeading___Toc275420442)

[***3.1 Genotype simulation*** 5](#__RefHeading___Toc275420443)

[***3.2 Phenotype simulation*** 5](#__RefHeading___Toc275420444)

[***3.3 Simulation procedure*** 5](#__RefHeading___Toc275420445)

[**Further discussion** 6](#__RefHeading___Toc275420446)

[**References:** 9](#__RefHeading___Toc275420461)

**Methods**

**1. Statistical exploration of optimal weights in the strong- and weak-clue sets**

Consider *m_S_ and m_W_* SNPs in the strong- and weak-clue sets. Their test *p*-values in a genome-wide association study are $(p_{1},\ldots,p_{m_{S}})$ and $(p_{1},\ldots,p_{m_{W}})$ respectively. These *p*-values correspond to standardized test statistics $(T_{1},\ldots,T_{m_{S}})$ and$(T_{1},\ldots,T_{m_{W}})$. In the strong-clue set, there are *m_0,S_* and *m_1,S_* SNPs following the null and alternative hypotheses respectively. The proportion of null hypotheses is $\pi_{0,S}=\frac{m_{0,S}}{m_{0,S}+m_{1,S}}$.The test statistics of null hypotheses are approximately under $\chi^{2}$ distribution with 1 degree of freedom (d.f.). The test statistics of alternative hypotheses are approximately$\chi^{2}(\delta_{S})$ distributed with 1 d.f. and a noncentrality parameter (NCP) *δ_S_.* Here we simply assume that all alternative hypotheses in the strong-clue set are independent and under the identical$\chi^{2}(\delta_{S})$distribution. In the present study, the NCP is also called signal strength. Similarly, in the weak-clue set, we can have *m_0,W_* null and *m_1,W_* alternative hypotheses. The proportion of null hypotheses is$\pi_{0,W}=\frac{m_{0,W}}{m_{0,W}+m_{1,W}}$. The test statistics of alternative hypotheses are independent and approximately$\chi^{2}(\delta_{W})$ distributed with 1 d.f. and NCP *δ_W_*.

***1.1. Estimate number of alternative hypotheses***

We slightly modified the method of Storey and Tibshirani (2003) (Storey and Tibshirani, 2003) to estimate the proportion of the true null hypotheses in both SNP sets. In the strong-clue set, we have the following procedure.

1. For a range of *λ*, say *λ*=0, 0.01, 0.02, . . . , 0.95, for *m_S_* p-values in a list, calculate

.

1. The estimate of$\pi_{0,S}$, , is equal to be median of.

The estimated number of alternative hypotheses in the strong-clue set is, where[x] indicates the largest integer equal to or less than x. The same procedure can be applied to the weak-clue set to estimate its number of alternative hypotheses, $\hat{m}_{1,W}$.

***1.2. Estimate signal strength (NCP) of alternative hypotheses***

We used the moment estimate for truncated non-central chi-squared distribution to infer the NCPs in the two different SNP sets. In the strong-clue set, given a cutoff *t* (*t*>0), the truncated expectation of a non-central chi-squared distribution is$E_{\delta}(T|T\geq t)=\int_{t}^{\infty} uf(u;\delta_{S})du/\int_{t}^{\infty} f(u;\delta_{S})du$, where$f(u;\delta_{S})$ is the probability density function of$\chi^{2}(\delta_{S})$ with 1 d.f. The probability density function has the form$f(u;\delta_{S})=e^{-(\delta_{S}+u)/2}\sum_{i=0}^{\infty} \frac{(\delta_{S}/2)^{i}u^{(1/2)+i-1}}{i!2^{(1/2)+i}\Gamma((1/2)+i)}\begin{matrix} , & (u>0) \end{matrix}$, where$\Gamma(x)=\int_{0}^{\infty} r^{x-1}e^{-r}dr$ is the gamma function. When *t*=0, it is a non-truncated expectation. For a chi-squared distribution (null hypothesis), the truncated expectation is$E(T|T\geq t)=\int_{t}^{\infty} uf(u)du/\int_{t}^{\infty} f(u)du$, where $f(u)=\frac{1}{2^{1/2}\Gamma(1/2)}u^{-1/2}e^{-u/2}\begin{matrix} , & (u>0) \end{matrix}$ is the probability density function of$\chi^{2}$with 1d.f.

In the strong-clue set, there are both alternative and null hypotheses with the ratio, (1*-π_0,S_*) to *π_0,S_*. The truncated expectation for a cutoff *t* in the mixture distribution is

$$\begin{matrix} \frac{(1-\pi_{0,S})\int_{t}^{\infty} f(u;\delta_{S})du}{(1-\pi_{0,S})\int_{t}^{\infty} f(u;\delta_{S})du+\pi_{0,S}\int_{t}^{\infty} f(u)du}E_{\delta_{S}}(T|T\geq t) \\ +\frac{\pi_{0,S}\int_{t}^{\infty} f(u)du}{(1-\pi_{0,S})\int_{t}^{\infty} f(u;\delta_{S})du+\pi_{0,S}\int_{t}^{\infty} f(u)du}E(T|T\geq t) \end{matrix}$$

Set the truncated expectation to be equal to the observed truncated mean *E_O_*(*t*) , we can construct an equation (according to the moment estimate).

A simplified form of the equation after algebraic transformation is

$E_{\delta_{S}}(T|T\geq t)=E_{O}(t)+\frac{\pi_{0,S}\int_{t}^{\infty} f(u)du}{(1-\pi_{0,S})\int_{t}^{\infty} f(u;\delta_{S})du}[E_{O}(t)-E(T|T\geq t)]$ (0)

The estimated NCP$\hat{\delta}_{S}$ can be obtained by solving the equation (0). In the equation, we set$\pi_{0,S}=\hat{\pi}_{0,S}$, $t=T_{(m_{1,S})}$, and $E(t)=\sum_{j=1}^{m_{1,S}} T_{(j)}/m_{1,S}$, where *T_(j)_* is the *jth* ordered statistics in the strong-clue set, $T_{(1)}\geq T_{(2)}\geq\cdots\geq T_{(m_{S})}$. According to Li and Yu (2008) , $E_{\delta_{S}}(T|T\geq t)$ is strictly increasing for *δ_S_* (*δ_S_* ≥0) (Li and Yu, 2008). We can use a bi-selection algorithm to find *δ_S_* very quickly. Li and Yu (2008) has also demonstrated that the moment estimate for a truncated non-central chi-square distribution (*t* >0) has smaller bias and root mean squared error than that for a non-truncated non-central chi-square distribution (*t* =0). The same deduction can be applied to the weak-clue set to get estimated NCP$\hat{\delta}_{W}$.

***1.3. Produce optimal weights***

Once the number and NCP of alternative hypotheses in both SNPs sets are obtained, we can start to explore the optimal weights. Denote the weights in the strong- and weak-clue sets by *w_S_* and *w_W_,* respectively. The weighted p-values are equal to *p_j_/w_S_* and *p_j_/w_W_* in the two sets respectively. According to Roeder et al. (2007), the family-wise error can be controlled if we constrain

$m_{1,S} w_{S} + m_{1,W}w_{W}=m_{1,S} +m_{1,W}$ (1)

We transform the non-central chi-square distribution into the normal distribution to calculate statistical power. Given a p-value rejection threshold *α,* the power of a single weighted test in the strong-clue set is

, where$\bar{\Phi}(x)=1-\Phi(x)$ is the complement of the standard normal cumulative distribution function (Roeder, et al., 2007). Correspondingly, the power of a single weighted test in the weak-clue set is

.

As we have *m*_1_ (=*m*_1_*_,S_* + *m*_1_*_,W_*) alternatively hypotheses in total, the average power of the tests on the whole genome is

$\bar{\eta}=\frac{1}{m_{1}}[m_{1,S}\eta(\delta_{S},w_{S})+m_{1,W}\eta(\delta_{W},w_{W})]\text{ }$ (2)

According to the prior information, we need favorably weight SNPs in the strong-clue set. Therefore we constrain $w_{S}\geq w_{W}$throughout the optimization process. In an equation, we can have

$w_{S}=w_{W}+D, \text{ (}D \geq0\text{) }$ (3)

The question now is to explore *w_S_* and *w_W_*, which can maximize the average power in equation 2, favorably weight SNPs in the strong-clue set by equation 3 and control the family-wise error by equation 1.

After adding the constraints as Lagrangian calculus into (2), we have

Set the derivative to zero and solve the following equation,

(4), where $\varphi(x)=\frac{\exp(-x^{2}/2)}{\sqrt{2\pi}}$is the density function of the standard normal distribution.

Reduce (4) to a simpler form,

$\left\{\begin{matrix} \exp(A\sqrt{\delta_{W}}-\delta_{W}/2)+\exp(-A\sqrt{\delta_{W}}-\delta_{W}/2)=\exp(B\sqrt{\delta_{S}}-\delta_{S}/2)+\exp(-B\sqrt{\delta_{S}}-\delta_{S}/2)\begin{matrix} & (5) \end{matrix} \\ w_{W}=1-\frac{m_{1,S}D}{m_{1}}\begin{matrix} & & (6) \end{matrix} \end{matrix}\right.$, where $A=\bar{\Phi}^{-1}(\frac{\alpha w_{W}}{2})$ and$B=\bar{\Phi}^{-1}(\frac{\alpha(w_{W}+D)}{2})$.

We need solve the equations (5) and (6) with $\delta_{S}=\hat{\delta}_{S}$ and $\delta_{W}=\hat{\delta}_{W}$ to find *w_S_* and *w_W_*.

As$w_{W}=1-\frac{m_{1,S}D}{m_{1}}\geq0$, we have $D\leq m_{1}/m_{1,S}$. Therefore, the range of *D* is [0, *m_1_/m_1,S_*]. The solution can also be obtained by a bi-selection algorithm very quickly.

**2. Theoretical calculation of power gain and power loss**

We first investigated the theoretical performance of this framework. The favorable weights (≥1.0) for SNPs in the strong-clue set will increase the power to identify a true associated SNP (namely, an alternative hypothesis). The increased power is called power gain and can be calculated theoretically. Similarly, the weights for SNPs in the weak-clue set are always ≤1.0 and will lead to power loss.

The power gain of one individual test is calculated by

. The power loss of one individual test can be calculated by

. Note that the$w_{S}$ is $\geq1$and $w_{W}$ is $\leq1$. In fact, once the signal strengths *δ_S_* and *δ_W_* are equal to 0, the power gain and power loss become the increased and decreased type I errors respectively.

**3. Computer simulation**

***3.1 Genotype simulation***

We modified the C++ source code of GWAsimulator (Li and Li, 2008) to generate genotypes of SNP for our simulation. This simulator adopted a moving-window algorithm (Durrant, et al., 2004) to produce multiple genotypes of SNPs. It required phased genotype data as a reference to preserve the linkage disequilibrium pattern. We used the HapMap CEU phased data on chromosomes 17, 19 and 20 as the reference. These HapMap SNPs not included by Affymetrix Genome-Wide Human SNP Array 6.0 were excluded, resulting in 28370 SNPs ultimately used in the simulation. The Mersenne Twister algorithm (http://www.math.sci.hiroshima-u.ac.jp/~m-mat/MT/emt.html) was used to generate random numbers throughout the simulation, in which a computer’s local clock was set as seed.

***3.2 Phenotype simulation***

The phenotypes of subjects were simulated under an *m*-locus disease model. Let *g_i_* = 0, 1, 2 denote the number of copies of the risk allele at SNP *i* (*i* = 1, …, *m*). The joint penetrance for the *m* genotypes $\{\text{g}_{1}\text{, }\ldots\text{, g}_{\text{m}}\}$, Pr(affected | *g*_1_, …, *g_m_*), can be calculated under a logistic model given allele frequencies, individual penetrances of genotypes, and the prevalence, *K,* . as described in the manual of GWAsimulator (http://biostat.mc.vanderbilt.edu/twiki/pub/Main/GWAsimulator/GWAsimulator_v2.0.pdf). The phenotype of an individual was determined by sampling without replacement under the uniform distribution *U*(0, 1). Given the genotypes of an individual at the *m* disease loci, if a sampled datum is less than the joint penetrance of these genotypes, the individual is coded affected; otherwise, it is unaffected. We assumed three genes (*GAPDHS*, *PRNP* and *ACE*), which were related to LOAD in a published Meta-analysis (Bertram, et al., 2007), as susceptibility genes of the simulated disease. Three SNPs (rs11882238 and rs12625444 and rs4351) with different minor allele frequencies (0.0750, 0.2167 and 0.4167) were selected as the disease predisposing loci from the three genes, respectively. Their minor alleles were defined as risk alleles, with allele frequencies ranging from relatively rare to very common. The prevalence of the disease was set to be 0.1.

***3.3 Simulation procedure***

First, genotypes and phenotypes of 12 000 cases and 12 000 controls were generated for each set of parameters. Subsequently, 200 samples with a given sample size (detailed below) were randomly drawn with replacement for basic allelic association tests. SNPs with minor allele frequency less than 0.01 were excluded before the association tests. In the downstream knowledge-based weighting analysis, SNPs were classified into the strong- and weak-clue sets. In the classification, an extended candidate gene set was used, which were derived by our candidate gene extension protocol based on a set of seed candidate genes. The seed candidate gene set was made up of 11 AD related genes with strong evidence [*AD5, BLMH, APBB2, PLAU, SORL1, PSEN2, PSEN1, MPO, APP, APOE* and *NOS3*] in the OMIM database and 12 genes (*ACE, CHRNB2, CST3, ESR1, GAPDHS, IDE, MTHFR, NCSTN, PRNP, TF, TFAM* and *TNF*) from a systematic meta-analysis of genetic association studies for LOAD (Bertram, et al., 2007). Finally, the optimal weights were explored to adjust the association *p-*values. The Benjamini and Hochberg (1995) method with the alpha level 0.05 was employed for multiple testing correction of the weighted *p-*values (Benjamini and Hochberg, 1995). The power to detect each SNP was defined as the proportion of its successful identification among the 200 samples. For each parameter setting, the procedure was repeated 100 times to standard error of the power estimate.

Two series of simulation were conducted. 1) The genetic risk of the susceptible heterozygote was increased from 1.1 to 1.65 (by 0.05 increment) under dominant and multiplicative genetic models (Risch and Merikangas, 1996) while the sample size was fixed to be 1200 cases and 1200 controls. 2) The sample size was increased from 2000 to 3400 (equal number of cases and controls) by a 400 increment unit under dominant and multiplicative genetic models while the genetic risk of the susceptible heterozygous was fixed to be 1.4. The genetic relative risk of a SNP’s susceptible heterozygous is equal to Pr(affected | heterozygote) / Pr(affected | non-risk homozygote).

**Further discussion**

The two-set classification seems too simple. There are at least two reasons why we only considered two different risk sets of SNPs. First, it is true that the prior information’s contribution to a true association is actually uncertain to some degree. For example, it is very hard to say that SNPs with a risk score of six are more likely to be a DSL than those with a risk score of seven given the incompleteness of knowledge. Nevertheless, we are more confident that they both have higher likelihood of being susceptible to the disease than SNPs with the risk score zero. The simplified two-set classification is a strategy to reduce the uncertainty involved. In contrast, more “artificial” SNP sets may inflate the uncertainty and negatively affect the performance of the method. Second, there will be fewer SNPs within each individual set for parameter estimating if more sets are considered. The reduced SNPs number will result in larger standard errors of the parameter estimates (i.e., the alternative hypothesis proportion and signal strength in each set). The inflated standard errors could also harm the overall performance of the statistical optimization even if the SNPs are correctly classified into multiple clue sets. As we have shown in the computer simulation and the real application to LOAD, the framework based on the two-set classification does have a great potential to achieve a satisfactory performance.

The basic assumptions of the weighting framework are (i) that the disease being tested has multiple susceptibility genetic factors, i.e. being multigenic, and (ii) that these genetic factors either share common features or are related to each other in terms of biological relevance. The first assumption is consistent with the definition of complex diseases (Reich and Lander, 2001). The second assumption has been widely adopted by many disease-gene prediction methods (Adie, et al., 2006; Aerts, et al., 2006; Kohler, et al., 2008; Wu, et al., 2008) and is supported by their successful applications in turn. In this study, we proposed a candidate-gene extension protocol to functionally connect potential susceptibility genes of a disease. The specific assumption is that genes sharing pathways and/or having PPIs with the seed-candidate genes are more likely to be the responsible genes of the same disease. Recently Li and Agarwal (2009) attempted to link diseases together based on shared biological pathways, in which disease genes are enriched. Upon collecting 4,195 disease-associated genes for 1,028 human diseases through literature mining, they found that averagely over 50% of the associated genes of each disease can be significantly mapped onto the pathways, implying that disease genes are related to each other in the form of pathways (Li and Agarwal, 2009). Similarly, genes of the same heterogeneous disease tend to have more PPIs (Oti and Brunner, 2007; Oti, et al., 2006). Oti et al. used 72,940 PPIs to prioritize candidate disease genes and found that their method could lead to a 10-fold enrichment compared with the original candidate gene set in their benchmark tests (Oti, et al., 2006). Our testing results for the candidate-gene extension protocol in the OMIM and GAD once again demonstrated that disease susceptibility genes do not function alone and that most of disease genes could be connected to another through biological pathways and PPIs. Although the noises or false positive signals in the GAD might weaken the persuasion of the results, it should also be noted that the pathways and PPI information are also far from being complete. The availability of more pathways and PPIs in the future may lead to more associated genes in the GAD to be introduced through the extension protocol. If the two aspects could offset each other, the conclusion might still be persuasive to some degree. The large and significant coverage, along with these coincident studies, support the second assumption of the present study.

The weighting framework can also be used for diseases without important candidate genes (defined as seed candidate genes in this study). In this situation, the framework will automatically select a number of top genes as the seed genes according to the SNP p-values to proceed. However, we still believe that preparation of a set of seed gene consisting of promising candidate genes is worthwhile, if available, because they may introduce more disease information into the analysis. An alternative way for a disease of limited candidate genes is to “borrow” the seed candidate genes from its phenotypically similar diseases. The rationale is based on a recent finding that phenotypically similar diseases may well have functionally related causative genes (Lage, et al., 2007; Oti and Brunner, 2007; Wood, et al., 2007; Wu, et al., 2008). The related genes can still be highlighted by our candidate gene extension strategy if they share the same biological pathways or have PPIs. This is the reason why we used genes of early-onset AD as part of the seed candidate genes of LOAD in the application.

The framework does not allow for varying size of genes and LD structure between neighborhood SNPs. Large genes tend to have more SNPs and thus are more likely to present a significant association by chance at one of their SNPs than small genes, particularly when imputed genotypes are used. Moreover, the dependence of SNPs complicates this problem further. Some available disease-gene prediction methods attempted to address this issue by assigning a single permuted or simulated association *p-*value to each gene (Holmans, et al., 2009; Wang, et al., 2007). Nevertheless, we cannot simply follow them because our weighting framework allows for the specific SNP features, such as the gene features and conservation of SNPs. For instance, our method treats SNPs in the exon region and intron region differently. So a method adjusting *p-*value by not only the gene size but also the SNPs’ prior information may be more reasonable. However, this idea needs to be carried out and further evaluated. Generally, the LD might inflate standard error of the statistic parameter estimation of the weighting procedure. The variable LD between SNPs could not be fully considered without resorting to simulation-based methods (for which the full genotype data are required). Although the dependence between SNPs was not considered in our statistical model, in the simulation we used dependent genotypes to investigate the performance of the framework. The broadly consistent results between the theoretical calculation, which assumed that SNPs were independent, and the simulation (which used depended SNPs) indicated that the LD might not substantially harm the performance of our weighting framework in practice. Anyhow, our weighting framework is only an initial stage in highlighting interesting SNPs and genes of complex diseases. The gene-size and LD issues will be specifically studied by more efforts in the feature.

This framework does not model the population structure. Theoretically, the population structure (if available) may inflate the significance level for both the strong-clue and weak-clue sets. The inflated significance may exaggerate the estimate of the number of alternative hypothesis and signal strength for both sets. As there is no bias to either of the sets, the exaggerated estimates may partly counteract each other in the process of optimal weight exploration. So it should not substantially affect the performance of our method. Anyhow, there are a number of methods (like the EIGENSTRAT (Price, et al., 2006)and Genomic Control (Devlin and Roeder, 1999)) available using genotypes to adjust population structure for GWAS. Uses can conveniently adjust the p-values by these methods/tools before the knowledge-based analysis by our tool. However, it should be also noted that how to perfectly get rid of the effect of population structure is still an open question (Kimmel, et al., 2007). It may be worth trying to prioritize the SNPs by weighting both the original and adjusted p-values through our framework. In our case study we used the original p-values because the genomic inflation factor for the 307448 *p-*values is small, 1.07125. According to the sources of sample, these are unlikely systematic ancestry differences in the sample. We believe that the slight inflation of moderate significances is partly attributable to potential susceptibility loci under the multigenic model of complex diseases as it is proposed for Schizophrenia and bipolar disorder (Purcell, et al., 2009).

In summary, we developed a novel knowledge-based integration framework to systematically highlight SNPs, particularly those with moderate association significances in GWAS for complex diseases. This framework was build upon both diverse and abundant biological resources, and solid statistic foundation. It had a user-friendly implementation by Java. Theoretically, it could largely increase the power of original GWAS to identify a susceptibility locus that can only present modest *p-*value but have sufficient biological implications. In a case study for LOAD, it highlighted some genes that were reported to be associated with LOAD in one or more published independent studies and two promising LOAD related pathways. Taken together, our integration framework would potentially improve the power of current GWAS for complex diseases.

**References:**

Adie, E.A., Adams, R.R., Evans, K.L., Porteous, D.J. and Pickard, B.S. (2006) SUSPECTS: enabling fast and effective prioritization of positional candidates, *Bioinformatics*, **22**, 773-774.

Aerts, S., Lambrechts, D., Maity, S., Van Loo, P., Coessens, B., De Smet, F., Tranchevent, L.C., De Moor, B., Marynen, P., Hassan, B., Carmeliet, P. and Moreau, Y. (2006) Gene prioritization through genomic data fusion, *Nat Biotechnol*, **24**, 537-544.

Benjamini, Y. and Hochberg, Y. (1995) Controlling the False Discovery Rate - a Practical and Powerful Approach to Multiple Testing, *J Roy Stat Soc B Met*, **57**, 289-300.

Bertram, L., McQueen, M.B., Mullin, K., Blacker, D. and Tanzi, R.E. (2007) Systematic meta-analyses of Alzheimer disease genetic association studies: the AlzGene database, *Nat Genet*, **39**, 17-23.

Devlin, B. and Roeder, K. (1999) Genomic control for association studies, *Biometrics*, **55**, 997-1004.

Durrant, C., Zondervan, K.T., Cardon, L.R., Hunt, S., Deloukas, P. and Morris, A.P. (2004) Linkage disequilibrium mapping via cladistic analysis of single-nucleotide polymorphism haplotypes, *Am J Hum Genet*, **75**, 35-43.

Holmans, P., Green, E.K., Pahwa, J.S., Ferreira, M.A., Purcell, S.M., Sklar, P., Owen, M.J., O'Donovan, M.C. and Craddock, N. (2009) Gene ontology analysis of GWA study data sets provides insights into the biology of bipolar disorder, *Am J Hum Genet*, **85**, 13-24.

Kimmel, G., Jordan, M.I., Halperin, E., Shamir, R. and Karp, R.M. (2007) A randomization test for controlling population stratification in whole-genome association studies, *Am J Hum Genet*, **81**, 895-905.

Kohler, S., Bauer, S., Horn, D. and Robinson, P.N. (2008) Walking the interactome for prioritization of candidate disease genes, *Am J Hum Genet*, **82**, 949-958.

Lage, K., Karlberg, E.O., Storling, Z.M., Olason, P.I., Pedersen, A.G., Rigina, O., Hinsby, A.M., Tumer, Z., Pociot, F., Tommerup, N., Moreau, Y. and Brunak, S. (2007) A human phenome-interactome network of protein complexes implicated in genetic disorders, *Nat Biotechnol*, **25**, 309-316.

Li, C. and Li, M. (2008) GWAsimulator: a rapid whole-genome simulation program, *Bioinformatics*, **24**, 140-142.

Li, Q.Z. and Yu, K. (2008) Inference of non-centrality parameter of a truncated non-central chi-squared distribution, *Journal of Statistical Planning and Inference*, **in Press**.

Li, Y. and Agarwal, P. (2009) A pathway-based view of human diseases and disease relationships, *PLoS ONE*, **4**, e4346.

Oti, M. and Brunner, H.G. (2007) The modular nature of genetic diseases, *Clin Genet*, **71**, 1-11.

Oti, M., Snel, B., Huynen, M.A. and Brunner, H.G. (2006) Predicting disease genes using protein-protein interactions, *J Med Genet*, **43**, 691-698.

Price, A.L., Patterson, N.J., Plenge, R.M., Weinblatt, M.E., Shadick, N.A. and Reich, D. (2006) Principal components analysis corrects for stratification in genome-wide association studies, *Nat Genet*, **38**, 904-909.

Purcell, S.M., Wray, N.R., Stone, J.L., Visscher, P.M., O'Donovan, M.C., Sullivan, P.F. and Sklar, P. (2009) Common polygenic variation contributes to risk of schizophrenia and bipolar disorder, *Nature*, **460**, 748-752.

Reich, D.E. and Lander, E.S. (2001) On the allelic spectrum of human disease, *Trends Genet*, **17**, 502-510.

Risch, N. and Merikangas, K. (1996) The future of genetic studies of complex human diseases, *Science*, **273**, 1516-1517.

Roeder, K., Devlin, B. and Wasserman, L. (2007) Improving power in genome-wide association studies: weights tip the scale, *Genet Epidemiol*, **31**, 741-747.

Storey, J.D. and Tibshirani, R. (2003) Statistical significance for genomewide studies, *Proc Natl Acad Sci U S A*, **100**, 9440-9445.

Wang, K., Li, M. and Bucan, M. (2007) Pathway-Based Approaches for Analysis of Genomewide Association Studies, *Am J Hum Genet*, **81**.

Wood, L.D., Parsons, D.W., Jones, S., Lin, J., Sjoblom, T., Leary, R.J., Shen, D., Boca, S.M., Barber, T., Ptak, J., Silliman, N., Szabo, S., Dezso, Z., Ustyanksky, V., Nikolskaya, T., Nikolsky, Y., Karchin, R., Wilson, P.A., Kaminker, J.S., Zhang, Z., Croshaw, R., Willis, J., Dawson, D., Shipitsin, M., Willson, J.K., Sukumar, S., Polyak, K., Park, B.H., Pethiyagoda, C.L., Pant, P.V., Ballinger, D.G., Sparks, A.B., Hartigan, J., Smith, D.R., Suh, E., Papadopoulos, N., Buckhaults, P., Markowitz, S.D., Parmigiani, G., Kinzler, K.W., Velculescu, V.E. and Vogelstein, B. (2007) The genomic landscapes of human breast and colorectal cancers, *Science*, **318**, 1108-1113.

Wu, X., Jiang, R., Zhang, M.Q. and Li, S. (2008) Network-based global inference of human disease genes, *Mol Syst Biol*, **4**, 189.
