# Supplementary material for: A Knowledge-Based Weighting Framework to Boost the Power of Genome-Wide Association Studies
Source: PLoS One. 2010 Dec 31;5(12):e14480. doi: 10.1371/journal.pone.0014480 (PMC3013112; doi:10.1371/journal.pone.0014480)
Supplement: Table S1 — (0.04 MB DOC) [file pone.0014480.s008.doc]

Table S1 Assignment of Risk scores for SNPs

| I: According to gene features where SNPs are located |
| --- |
| | ***Feature*** | ***Description*** | ***Risk Scores*** | | --- | --- | --- | | intergene/- | Beyond 2 Kb 5′ or 500 bp 3′ of a gene (on either strand) | 0 | | intron | In the intron of a gene but not in the first two or last two bases of the intron | 1 | | close-to-gene | Within 2 Kb 5′ or 500 bp 3′ of a gene (on either strand), but the variation is not in the transcript for the gene | 2 | | mrna-utr | In the transcript of a gene but not in the coding region of the transcript | 2 | | coding- synonymous | Within the coding region of a gene but not change amino acid sequences of a gene | 3 | | splice-site | In the first two or last two bases of the intron | 4 | | coding-non synonymous | Within the coding region of a gene and change amino acid sequences of a gene | 4 | |
|  |
| **II: According to non-gene properties** |
| - If [UCSC Conservation Score >= X (0.8 as default)], then [RiskScore = RiskScore+2] - If [Selection Score >= Y(2.0 as default)], then [RiskScore = RiskScore+2] - If [miRNA binding site], then [RiskScore = RiskScore+2] |
|  |
| **III: According to candidate-gene set** |
| - If [Within a candidate gene] and [RiskScore =RiskScore+3] |
