# Supplementary material for: A Knowledge-Based Weighting Framework to Boost the Power of Genome-Wide Association Studies
Source: PLoS One. 2010 Dec 31;5(12):e14480. doi: 10.1371/journal.pone.0014480 (PMC3013112; doi:10.1371/journal.pone.0014480)
Supplement: Table S3 — (0.11 MB DOC) [file pone.0014480.s009.doc]

Table S3: Simulation results under dominant model

| **^a^R.R.** |  | **^d^No Weight** | **^e^Exude Three** | **^f^ACE** | **^g^ACE+GAPDHS** | **^h^Include Three** |
| --- | --- | --- | --- | --- | --- | --- |
| **1.10** | **^b^rs4351** | 0.000755±6.26e-06 | 0.000755±6.26e-06 | 0.00164±1.52e-05 | 0.00164±1.52e-05 | 0.00164±1.52e-05 |
|  | **^b^rs11882238** | 0.000126±8.39e-07 | 0.000126±8.39e-07 | 0.000126±8.39e-07 | 0.000629±5.58e-06 | 0.000503±4.87e-06 |
|  | **^b^rs12625444** | 0.000503±3.16e-06 | 0.000503±3.16e-06 | 0.000503±3.16e-06 | 0.000503±3.16e-06 | 0.00201±1.47e-05 |
|  | **^c^#Av. False** | 0.179±0.00427 | 0.315±0.00926 | 0.311±0.0092 | 0.312±0.00975 | 0.309±0.00939 |
| **1.15** | **rs4351** | 0.00151±1.14e-05 | 0.00151±1.14e-05 | 0.0039±4.01e-05 | 0.00352±3.52e-05 | 0.00403±4.16e-05 |
|  | **rs11882238** | 0.000252±1.64e-06 | 0.000252±1.64e-06 | 0.000252±1.64e-06 | 0.00126±8.64e-06 | 0.00126±8.64e-06 |
|  | **rs12625444** | 0.00302±5.4e-05 | 0.00302±5.4e-05 | 0.00302±5.4e-05 | 0.00302±5.4e-05 | 0.00881±0.000164 |
|  | **# Av. False** | 0.183±0.00495 | 0.303±0.01 | 0.305±0.0103 | 0.303±0.0101 | 0.311±0.0108 |
| **1.20** | **rs4351** | 0.00385±4.24e-05 | 0.00385±4.24e-05 | 0.0142±0.00018 | 0.0142±0.000155 | 0.0146±0.000173 |
|  | **rs11882238** | 0.000769±4.63e-06 | 0.000769±4.63e-06 | 0.000769±4.63e-06 | 0.00603±6.67e-05 | 0.00641±6.96e-05 |
|  | **rs12625444** | 0.00872±0.000132 | 0.00872±0.000132 | 0.00872±0.000132 | 0.00872±0.000132 | 0.0247±0.000558 |
|  | **# Av. False** | 0.202±0.0048 | 0.325±0.00803 | 0.328±0.00855 | 0.327±0.00767 | 0.337±0.00853 |
| **1.25** | **rs4351** | 0.0178±0.000224 | 0.0179±0.000221 | 0.0494±0.000854 | 0.0512±0.00096 | 0.0537±0.00107 |
|  | **rs11882238** | 0.00346±1.83e-05 | 0.00346±1.83e-05 | 0.00346±1.83e-05 | 0.0124±0.000136 | 0.0128±0.000144 |
|  | **rs12625444** | 0.0283±0.000387 | 0.0283±0.000387 | 0.0285±0.000387 | 0.0286±0.000386 | 0.0668±0.00149 |
|  | **# Av. False** | 0.215±0.00597 | 0.351±0.00926 | 0.361±0.0108 | 0.363±0.0105 | 0.378±0.011 |
| **1.30** | **rs4351** | 0.0555±0.00135 | 0.0555±0.00135 | 0.104±0.00325 | 0.108±0.00327 | 0.116±0.00365 |
|  | **rs11882238** | 0.0137±0.00018 | 0.0138±0.000178 | 0.0137±0.00018 | 0.0453±0.00112 | 0.0487±0.00122 |
|  | **rs12625444** | 0.0717±0.00267 | 0.0717±0.00267 | 0.0719±0.00267 | 0.0719±0.00268 | 0.155±0.00738 |
|  | **# Av. False** | 0.258±0.00624 | 0.414±0.0105 | 0.437±0.013 | 0.455±0.0153 | 0.484±0.0164 |
| **1.35** | **rs4351** | 0.127±0.00332 | 0.127±0.00334 | 0.219±0.00558 | 0.227±0.00563 | 0.248±0.00626 |
|  | **rs11882238** | 0.04±0.000945 | 0.04±0.000945 | 0.0403±0.000945 | 0.0912±0.00251 | 0.101±0.00299 |
|  | **rs12625444** | 0.17±0.00488 | 0.17±0.00492 | 0.17±0.00488 | 0.17±0.00491 | 0.314±0.00888 |
|  | **# Av. False** | 0.336±0.0119 | 0.542±0.0214 | 0.583±0.0225 | 0.607±0.0235 | 0.655±0.0271 |
| **1.40** | **rs4351** | 0.249±0.011 | 0.25±0.011 | 0.369±0.0142 | 0.382±0.0145 | 0.406±0.0153 |
|  | **rs11882238** | 0.0878±0.00249 | 0.0878±0.00249 | 0.0878±0.00249 | 0.178±0.00594 | 0.195±0.00627 |
|  | **rs12625444** | 0.378±0.0143 | 0.378±0.0143 | 0.378±0.0143 | 0.378±0.0143 | 0.558±0.0126 |
|  | **# Av. False** | 0.457±0.02 | 0.749±0.0373 | 0.805±0.0421 | 0.831±0.0445 | 0.887±0.0469 |
| **1.45** | **rs4351** | 0.388±0.0167 | 0.388±0.0167 | 0.517±0.0171 | 0.537±0.0165 | 0.559±0.0167 |
|  | **rs11882238** | 0.182±0.00579 | 0.182±0.00579 | 0.182±0.00582 | 0.322±0.00922 | 0.339±0.00948 |
|  | **rs12625444** | 0.513±0.0162 | 0.513±0.0162 | 0.514±0.0162 | 0.514±0.0162 | 0.692±0.0117 |
|  | **# Av. False** | 0.658±0.037 | 1.01±0.0824 | 1.07±0.0806 | 1.12±0.0849 | 1.19±0.086 |
| **1.50** | **rs4351** | 0.537±0.0142 | 0.537±0.0142 | 0.653±0.0108 | 0.678±0.01 | 0.695±0.00997 |
|  | **rs11882238** | 0.312±0.0115 | 0.312±0.0115 | 0.313±0.0117 | 0.465±0.0122 | 0.478±0.0122 |
|  | **rs12625444** | 0.719±0.0128 | 0.719±0.0127 | 0.72±0.0128 | 0.72±0.0127 | 0.844±0.00635 |
|  | **# Av. False** | 0.858±0.0481 | 1.35±0.0912 | 1.4±0.0939 | 1.47±0.099 | 1.52±0.102 |
| **1.55** | **rs4351** | 0.693±0.0109 | 0.693±0.0109 | 0.787±0.00749 | 0.804±0.00679 | 0.81±0.00656 |
|  | **rs11882238** | 0.428±0.017 | 0.428±0.0171 | 0.428±0.0171 | 0.592±0.0156 | 0.599±0.0157 |
|  | **rs12625444** | 0.86±0.00346 | 0.86±0.00346 | 0.861±0.00344 | 0.861±0.00342 | 0.932±0.00152 |
|  | **# Av. False** | 1.14±0.0877 | 1.76±0.174 | 1.82±0.175 | 1.9±0.179 | 1.92±0.18 |
| **1.60** | **rs4351** | 0.809±0.00795 | 0.809±0.00796 | 0.875±0.00422 | 0.886±0.00366 | 0.89±0.00348 |
|  | **rs11882238** | 0.592±0.0145 | 0.592±0.0145 | 0.592±0.0145 | 0.736±0.0105 | 0.74±0.0102 |
|  | **rs12625444** | 0.933±0.00174 | 0.933±0.00174 | 0.934±0.00171 | 0.934±0.00172 | 0.971±0.000551 |
|  | **# Av. False** | 1.44±0.112 | 2.18±0.184 | 2.24±0.181 | 2.31±0.186 | 2.32±0.193 |
| **1.65** | **rs4351** | 0.891±0.00348 | 0.891±0.00348 | 0.932±0.00176 | 0.938±0.00159 | 0.938±0.0016 |
|  | **rs11882238** | 0.722±0.0145 | 0.722±0.0145 | 0.722±0.0145 | 0.83±0.00886 | 0.831±0.00883 |
|  | **rs12625444** | 0.972±0.000548 | 0.972±0.000548 | 0.972±0.000548 | 0.972±0.000548 | 0.987±0.000158 |
|  | **# Av. False** | 1.63±0.12 | 2.38±0.175 | 2.43±0.176 | 2.48±0.171 | 2.49±0.17 |

^a^: Genetic Relative Risk (See definition in Supplementary Methods 4.2); ^b^: Monte Carlo mean of the estimated power (±Monte Carlo Standard Deviation of the estimated power) to detect the corresponding SNPs; ^c^: Averaged number of false positive discoveries (±Standard Deviation) to among 28370 tests (SNPs); ^d^: results of basic allelic association test without any weight; ^e^: results of weighted basic allelic association test while none of the three SNPs are not in the strong-clue set; ^f^: results of weighted basic allelic association test while only one SNP **rs4351** is in the strong-clue set; ^g^: power of weighted basic allelic association test while two SNPs **rs4351** and **rs11882238** are in the strong-clue set; ^h^: power of weighted basic allelic association test while all the three SNPs are in the strong-clue set. We assume a SNP will be classified into the strong-clue set once its gene is included in the candidate gene set.
