# Supplementary material for: A Knowledge-Based Weighting Framework to Boost the Power of Genome-Wide Association Studies
Source: PLoS One. 2010 Dec 31;5(12):e14480. doi: 10.1371/journal.pone.0014480 (PMC3013112; doi:10.1371/journal.pone.0014480)
Supplement: Table S2 — (0.11 MB DOC) [file pone.0014480.s010.doc]

Table S2: 14 genes suggested contributing to LOAD by at least one previous independent study

| **Gene**^a^ | **Population** | **Sample Size** | **Minimal *p-*value**^b^ | **Reference** |
| --- | --- | --- | --- | --- |
| GAB2(rs7115850: 7.22E-4 -> 9.30E-5) | German | 491 cases and 479 controls | 0.033(Haplotype) | (Feulner, et al., 2009) |
|  | Netherlands | A population-based cohort study (n = 5507; age > 55) with 443 incident AD cases | 3.0E-3 **(among APOEε4 carrier)** | (Ikram, et al., 2009) |
|  | Italian | 241 cases and 338 controls | 0.005 | (Nacmias, et al., 2009) |
|  | Belgium | 528 cases and 601 controls | 0.02 (corrected by simulation) | (Sleegers, et al., 2009) |
| ATF7(rs11170573: 7.48E-4 ->9.63E-5) | USA | 567 families | 0.0026 | (Lin, et al., 2006) |
| ATXN1(rs7750263: 1.66E-3 ->2.14E-4) | USA (NIMH) | 435 families | 0.008 | (Bertram, et al., 2008) |
|  | Belgium | 1078 cases and controls | 0.01 | (Bettens, et al., 2009) |
| COL11A1(rs1763351: 4.10E-4-> 5.27E-05) | Japn | 376 AD patients and 376 control | 0.03 | (Taguchi, et al., 2005) |
| NRG1(rs1487155:9.20E-4->1.18E-4) | USA (NIMH) | 65 families | 0.008 | (Go, et al., 2005) |
| MAGI2(rs1978326:1.01E-3->1.30E-4) | USA (ADNI) | 172 AD patients and 209 control | 2.85E-6 | (Potkin, et al., 2009) |
| PRSS7(rs9979147: 1.10E-3->1.41E-4) | Japn | 374 AD patients and 375 control | 0.0059 | (Kimura, et al., 2007) |
| IL1RN(rs315920:1.62E-3->2.09E-4) | Italy | 318 AD patients and 355 control |  | (Grimaldi, et al., 2000) |
|  | Italy | 237 AD patients and 147 control |  | (Seripa, et al., 2005) |
|  | USA | 124 AD patients and 97 control |  | (Seripa, et al., 2005) |
|  | USA | 179 AD patients and 517 control |  | (Yucesoy, et al., 2006) |
| SNCA(rs8180214: 2.74E-3->3.53E-4) | USA | 223 AD patients and 149 control |  | (Xia, et al., 1996) |
| LRP2(rs2239594:2.97E-3->3.83E-4) | Japan | 183 AD patients and 210 control |  | (Matsubara, et al., 2001) |
|  | Spain | 1158 AD patients and 1025 control | 0.03 | (Vargas, et al., 2010) |
| KCNJ6(rs6517434:3.17E-3->4.081E-4) | Japan | 374 AD patients and 375 control | 0.024 | (Kimura, et al., 2007) |
| EFNA5 (rs152562: 3.23 E-3->4.16E-4) | USA (ADNI) | 172 AD patients and 209 control | 2.15E-7 | (Potkin, et al., 2009) |
| ADAM12(rs3847470:3.71E-3 ->4.78E-4) | UK | 1051 AD patients and 1269 control |  | (Harold, et al., 2007) |
| F13A1(rs3024346: 3.88E-3 ->4.99E-4) | Italy | 90 AD patients and 139 control | <0.01 | (Gerardino, et al., 2006) |

a: Official gene symbol (highlight SNP: original *p-*value->weighted *p-*value); b: the most association *p-*values of SNP belonging to this gene; n.a.: not available; NIMH: Institute of Mental Health Genetics Initiative Study; CAG: Consortium on Alzheimer's Genetics; CAP: Collaborative Alzheimer Project.
