# Supplementary material for: A Knowledge-Based Weighting Framework to Boost the Power of Genome-Wide Association Studies
Source: PLoS One. 2010 Dec 31;5(12):e14480. doi: 10.1371/journal.pone.0014480 (PMC3013112; doi:10.1371/journal.pone.0014480)
Supplement: Table S4 — (0.11 MB DOC) [file pone.0014480.s011.doc]

Table S4: Simulation results under multiplicative model

| **^a^R.R.** |  | **^d^Weight** | **^e^Exude Three** | **^f^ACE** | **^g^ACE+GAPDHS** | **^h^Include Three** |
| --- | --- | --- | --- | --- | --- | --- |
| **1.10** | **^b^rs4351** | 0.0012±1.02e-05 | 0.0012±1.02e-05 | 0.0064±6.55e-05 | 0.00633±6.14e-05 | 0.00653±6.64e-05 |
|  | **^b^rs11882238** | 0.000133±8.8e-07 | 0.000133±8.8e-07 | 0.000133±8.8e-07 | 0.000867±9.57e-06 | 8e-04±9.23e-06 |
|  | **^b^rs12625444** | 0.000667±4.04e-06 | 0.000667±4.04e-06 | 0.000667±4.04e-06 | 0.000667±4.04e-06 | 0.00347±3.28e-05 |
|  | **^c^#Av. False** | 0.188±0.00369 | 0.312±0.00654 | 0.309±0.00677 | 0.309±0.00664 | 0.309±0.0068 |
| **1.15** | **rs4351** | 0.0149±0.000244 | 0.0149±0.000244 | 0.0397±0.00108 | 0.0399±0.00103 | 0.0402±0.00106 |
|  | **rs11882238** | 0.000667±6.73e-06 | 0.000667±6.73e-06 | 0.000667±6.73e-06 | 0.00273±2.97e-05 | 0.00273±3.06e-05 |
|  | **rs12625444** | 0.00547±8.65e-05 | 0.00547±8.65e-05 | 0.00547±8.65e-05 | 0.00547±8.65e-05 | 0.019±0.000465 |
|  | **# Av. False** | 0.196±0.00546 | 0.325±0.00882 | 0.337±0.00938 | 0.339±0.0095 | 0.341±0.00959 |
| **1.20** | **rs4351** | 0.0829±0.00222 | 0.0831±0.00221 | 0.158±0.00553 | 0.159±0.00541 | 0.166±0.00591 |
|  | **rs11882238** | 0.002±1.48e-05 | 0.002±1.48e-05 | 0.002±1.48e-05 | 0.00847±7.62e-05 | 0.0088±8.34e-05 |
|  | **rs12625444** | 0.027±0.000736 | 0.027±0.000736 | 0.027±0.000736 | 0.027±0.000736 | 0.0716±0.00224 |
|  | **# Av. False** | 0.289±0.00979 | 0.459±0.018 | 0.499±0.0214 | 0.499±0.0213 | 0.516±0.0208 |
| **1.25** | **rs4351** | 0.265±0.0121 | 0.265±0.0121 | 0.39±0.0148 | 0.394±0.0148 | 0.413±0.015 |
|  | **rs11882238** | 0.00913±0.000109 | 0.0092±0.00011 | 0.00913±0.000109 | 0.0336±0.000665 | 0.0369±0.000755 |
|  | **rs12625444** | 0.131±0.00391 | 0.131±0.00391 | 0.131±0.0039 | 0.131±0.00389 | 0.257±0.00759 |
|  | **# Av. False** | 0.544±0.0454 | 0.859±0.0994 | 0.92±0.105 | 0.93±0.106 | 0.98±0.113 |
| **1.30** | **rs4351** | 0.527±0.0154 | 0.527±0.0153 | 0.648±0.0137 | 0.656±0.0133 | 0.681±0.0127 |
|  | **rs11882238** | 0.0361±0.001 | 0.0362±0.00101 | 0.0363±0.00101 | 0.0959±0.00385 | 0.104±0.00414 |
|  | **rs12625444** | 0.335±0.0132 | 0.335±0.0132 | 0.336±0.0132 | 0.336±0.0132 | 0.511±0.0141 |
|  | **# Av. False** | 0.948±0.0643 | 1.43±0.113 | 1.5±0.113 | 1.53±0.115 | 1.61±0.114 |
| **1.35** | **rs4351** | 0.777±0.00996 | 0.777±0.00995 | 0.852±0.0053 | 0.861±0.00481 | 0.874±0.0043 |
|  | **rs11882238** | 0.0924±0.00277 | 0.0925±0.00276 | 0.0926±0.00278 | 0.193±0.00609 | 0.201±0.00626 |
|  | **rs12625444** | 0.588±0.0141 | 0.588±0.0141 | 0.588±0.0141 | 0.588±0.0141 | 0.745±0.0103 |
|  | **# Av. False** | 1.51±0.118 | 2.18±0.16 | 2.26±0.167 | 2.31±0.168 | 2.38±0.172 |
| **1.40** | **rs4351** | 0.928±0.00233 | 0.928±0.00232 | 0.958±0.00105 | 0.962±0.000901 | 0.965±0.000773 |
|  | **rs11882238** | 0.205±0.00874 | 0.205±0.00874 | 0.205±0.00875 | 0.351±0.0125 | 0.358±0.0128 |
|  | **rs12625444** | 0.794±0.0107 | 0.794±0.0107 | 0.794±0.0107 | 0.794±0.0107 | 0.888±0.00515 |
|  | **# Av. False** | 2.21±0.176 | 3.01±0.223 | 3.11±0.234 | 3.19±0.237 | 3.23±0.239 |
| **1.45** | **rs4351** | 0.984±0.000176 | 0.984±0.000175 | 0.991±8e-05 | 0.992±7.81e-05 | 0.993±7.12e-05 |
|  | **rs11882238** | 0.3±0.0108 | 0.3±0.0109 | 0.3±0.0108 | 0.462±0.0131 | 0.465±0.0131 |
|  | **rs12625444** | 0.922±0.00283 | 0.922±0.00283 | 0.922±0.00282 | 0.922±0.00281 | 0.964±0.000836 |
|  | **# Av. False** | 2.88±0.156 | 3.75±0.189 | 3.87±0.211 | 3.96±0.213 | 3.98±0.214 |
| **1.50** | **rs4351** | 0.997±3.1e-05 | 0.997±3.1e-05 | 0.998±1.43e-05 | 0.998±1.28e-05 | 0.998±1.28e-05 |
|  | **rs11882238** | 0.472±0.0139 | 0.472±0.0139 | 0.472±0.0139 | 0.636±0.0119 | 0.638±0.0118 |
|  | **rs12625444** | 0.982±0.000267 | 0.982±0.000267 | 0.982±0.000267 | 0.982±0.000267 | 0.993±6.69e-05 |
|  | **# Av. False** | 3.4±0.205 | 4.28±0.234 | 4.44±0.245 | 4.52±0.252 | 4.52±0.251 |
| **1.55** | **rs4351** | 1±2.13e-06 | 1±2.13e-06 | 1±1.72e-06 | 1±8.8e-07 | 1±8.8e-07 |
|  | **rs11882238** | 0.595±0.0144 | 0.595±0.0144 | 0.595±0.0144 | 0.743±0.00875 | 0.743±0.00874 |
|  | **rs12625444** | 0.996±4.58e-05 | 0.996±4.58e-05 | 0.996±4.58e-05 | 0.996±4.58e-05 | 0.998±9.98e-06 |
|  | **# Av. False** | 3.88±0.219 | 4.8±0.246 | 4.97±0.265 | 5.05±0.255 | 5.05±0.255 |
| **1.60** | **rs4351** | 1±8.8e-07 | 1±8.8e-07 | 1±0 | 1±0 | 1±0 |
|  | **rs11882238** | 0.741±0.011 | 0.741±0.011 | 0.741±0.011 | 0.845±0.00596 | 0.845±0.00596 |
|  | **rs12625444** | 0.999±7.23e-06 | 0.999±7.23e-06 | 0.999±7.23e-06 | 0.999±7.23e-06 | 1±2.13e-06 |
|  | **# Av. False** | 4.37±0.187 | 5.31±0.26 | 5.49±0.271 | 5.53±0.265 | 5.53±0.265 |
| **1.65** | **rs4351** | 1±0 | 1±0 | 1±0 | 1±0 | 1±0 |
|  | **rs11882238** | 0.836±0.00674 | 0.836±0.00674 | 0.836±0.00674 | 0.913±0.0027 | 0.913±0.0027 |
|  | **rs12625444** | 1±4.44e-07 | 1±4.44e-07 | 1±4.44e-07 | 1±4.44e-07 | 1±0 |
|  | **# Av. False** | 4.77±0.202 | 5.76±0.245 | 5.95±0.246 | 5.98±0.251 | 5.98±0.251 |

^a^: ^b^: ^c^: ^d^: ^e^: ^f^: ^g^: ^h^: have the same mining as those in Table S3.
