# Supplementary material for: A Knowledge-Based Weighting Framework to Boost the Power of Genome-Wide Association Studies
Source: PLoS One. 2010 Dec 31;5(12):e14480. doi: 10.1371/journal.pone.0014480 (PMC3013112; doi:10.1371/journal.pone.0014480)
Supplement: Table S5 — (0.24 MB DOC) [file pone.0014480.s012.doc]

Table S5: Coverage percentages of positively associated genes in the GAD by the extension of seed candidate genes in OMIM database

| **MeSH Disease Name** | **OMIM Seed Candidate Genes (**^a^**Symbols)** | **Positively Associated Genes (**^a^**Symbols) in GAD** | **Number of Positively Associated Genes** | **Number of Positively Associated Genes in Extended Gene Set** | **Overage Percentage** | ^b^**p-value** |
| --- | --- | --- | --- | --- | --- | --- |
| Diabetes Mellitus | ABCC8, AKT2, CAPN10, CCR5, CDKAL1, CTLA4, ENPP1, FOXC2, FOXP3, GCGR, GCK, GLIS3, GPD2, HNF1A, HNF1B, HNF4A, IGF2BP2, INSR, IPF1, IRS1, IRS2, KCNJ11, LIPC, MAPK8IP1, MBL2, NEUROD1, OAS1, PAX4, PTF1A, PTPN22, RETN, SH2B3, SLC2A2, SLC2A4, SLC30A8, SUMO4, TCF7L2, WFS1, ZFP57 | ABCA1, ABCC8, ACE, ACP1, ACSM3, ADH1C, ADIPOQ, ADIPOR1, ADIPOR2, ADRB2, ADRB3, AGER, AGRP, AGT, AHSG, ALDOB, APOA1, APOA4, APOA5, APOC3, APOE, APOM, ARHGEF12, ATF6, ATP1A1, BAT2, BCHE, BTC, CAPN10, CASP7, CASQ1, CBLB, CCL2, CCR2, CCR5, CD14, CD36, CD3E, CD4, CDC2L2, CDKAL1, CDKN2A, CDKN2B, CEL, CETP, CHRM3, CLPS, CRP, CTLA4, CXCL12, CYP19A1, CYP2C9, CYP2D6, CYP2R1, DIO2, DRD2, DUSP12, ENDOGL1, ENPP1, EPHX2, F3, FABP1, FABP2, FAS, FASLG, FOXC2, FOXP3, FTO, FXN, GCGR, GCK, GHRL, GNB3, GPD2, GYS1, HHEX, HLA, HLA-A, HLA-C, HLA-DPB1, HLA-DQA1, HLA-DQB1, HLA-DRA, HLA-DRB1, HNF4A, HSD11B1, HSD17B1, HYOU1, IAPP, ICAM1, IDDM2, IDE, IFNG, IGF1, IGF2, IGF2BP2, IL10, IL12B, IL13, IL18, IL1R1, IL2RA, IL4, IL4R, IL6, IL6R, INS, INSR, IPF1, IRS1, IRS2, KCNJ11, KIR2DS2, KLF1, KLF7, LDLR, LEP, LEPR, LIPE, LMNA, LPL, LTA, MC3R, MET, MICA, MT-ND2, MT-TS2, MTTP, NAT2, NEUROD1, NEUROG3, NFKBIL1, NHE1, NOS2A, NOS3, NPPB, NPY, NPY2R, NR0B2, NR3C1, NR5A1, NRF1, OAS1, PARD6A, PARP1, PAX4, PBEF1, PCK1, PCSK2, PDCD1, PKLR, PLIN, PNPLA2, PON1, PPARA, PPARD, PPARG, PPARGC1A, PPARGC1B, PPP1R3, PPP1R3A, PRKAA2, PRKCZ, PSMA6, PSMB8, PSMB9, PTEN, PTGES2, PTGS2, PTPN1, PTPN22, PTPRF, PYY, REG1A, RETN, RXRG, SERPINE1, SLC11A1, SLC2A2, SLC30A8, SLCO1B1, SOCS2, SOD2, SOD3, SORBS1, SREBF1, STX1A, SUMO4, TAF5L, TAP2, TCF1, TCF7, TCF7L2, TFAP2B, TH, THBS2, TLR2, TNF, TNFRSF1B, UBL5, UCP1, UCP2, UCP3, USF1, UTS2, VDR, VLDLR, WFS1 | 208 | 170 | 81.73% | 1.12E-09 |
| Carcinoma | AXIN1, BRAF, CASP8, CDH1, CRTC1, CTNNB1, EGFR, ERBB2, FLCN, GOLGA5, HNF1A, HNF1B, HRAS, HRPT2, IGF2R, ING1, KRAS2, LZTS1, MAML2, MET, MINPP1, MSH3, NCOA4, NDUFA13, NRAS, NTRK1, OGG1, PCM1, PIK3CA, PRCC, PRKAR1A, PRKN, PTCH1, PTCH2, PTEN, RAD54B, RAD54L, RASA1, RBBP8, RET, RNF139, RNF6, RRAS2, RSPO1, SDHD, SMOH, TFE3, TNFRSF10B, TNFRSF6, TP53, TRIM24, TRIM33, TSHR, VHL, WWOX | ABCB1, ABCC2, ABCG2, ADH1C, AFP, AGER, ALDH2, APEX1, AR, AURKA, BARD1, BRAF, BRCA1, CCND1, CDH1, CDKN1A, CDKN1B, CDKN2A, CHEK2, COMT, CTLA4, CTNNB1, CYP19A1, CYP1A1, CYP1A2, CYP1B1, CYP2A6, CYP2E1, CYP3A4, CYP3A5, E2F1, ECG2, EGFR, EPHX1, ERCC1, ERCC2, ERCC5, ESR1, F7, FAS, FASLG, FGFR4, FHIT, GAS1, GNAS, GPX1, GSTA1, GSTM1, HFE, HIF1A, HLA-A, HLA-B, HLA-DQA1, HLA-DQB1, HLA-DRB1, HLA-E, HMGCR, HMOX1, HP, HSPA1B, Hras, IGF2R, IL1B, IL1RN, IL4R, IL8, ITGB3, KRAS, LEP, LIG1, LIPC, MBD4, MC1R, MDM2, MGMT, MLH1, MLH3, MMP1, MMP12, MMP2, MMP3, MMP9, MPO, MSH3, MTHFR, MYC, MYCL1, NAT1, NAT2, NME1, NOS2A, NOS3, NQO1, NRAS, NUDT1, OGG1, PAK1, PIP, PLAU, PMS1, POLG2, POLK, POLR2B, PPP1R13L, PTCH, PTEN, PTGS2, RAD51, RAGE, RET, RPA1, RPA3, SAI1, SERPINE1, SFTPB, SLC11A1, SOD2, STAT3, SULT1A1, TCF7L2, TFRC, TGFBR1, TIMP2, TNF, TP53, TP73, TP73L, TYMS, UGT1A1, UGT1A10, UGT1A7, UGT1A9, UQCRFS1, VDR, VEGFA, VHL, XPA, XRCC1, XRCC3 | 139 | 118 | 84.89% | 9.25E-10 |
| Hypertension | ADD1, AGT, AGTR1, BMPR2, CPS1, CYP11B2, CYP3A5, ECE1, GNB3, HSD11B2, KCNMB1, NOS3, NR3C2, PTGIS, RETN | ABCB1, ACADSB, ACE, ACE2, ACSM1, ACSM3, ADD1, ADD2, ADD3, ADIPOQ, ADM, ADRA1A, ADRA2B, ADRB1, ADRB2, AGT, AGTR1, AGTR2, AHR, ALOX12, APOB, APOC3, ATP1A1, BDKRB1, BDKRB2, BMPR2, CACNA1C, CALCA, CAPN10, CAPN5, CART, CAST, CAT, CAV1, CCR2, CES1, CFH, CFTR, CHGA, CLCNKB, CLU, CNR2, COL1A2, COMT, CORIN, CPS1, CTNS, CYBA, CYP11B2, CYP1A1, CYP2C8, CYP2C9, CYP2J2, CYP3A5, CYP4A11, CYP4F2, DRD1, DRD2, EDN1, EDN2, EDNRA, ENG, ENPP1, ESR2, F11, F7, FYN, GBA, GCGR, GCK, GHR, GHRL, GJA5, GNAI2, GNAS, GNB3, GPX1, GRK4, GYS1, HGF, HLA-A, HLA-B, HLA-DPB1, HLA-DQB1, HLA-DRB1, HMOX1, HSD11B1, HSD11B2, HTR2A, Hb, IKBKAP, INSR, ITGA2, KCNJ11, LDLR, LEP, LEPR, LIPC, LIPE, LPL, LRP5, MBOAT5, MTR, MTTP, NLRP3, NOS2A, NOS3, NPPA, NPPB, NPPC, NPR1, NPR2, NPR3, NPY, P2RY2, PLIN, PNMT, PON2, PPARG, PPARGC1A, PTGIS, QPCT, REN, RENBP, RETN, SA, SAA1, SAH, SCN7A, SCNN1A, SCNN1B, SCNN1G, SELE, SERPINE1, SGK, SLC12A1, SLC12A3, SLC14A2, SLC4A1, SLC6A2, SLC6A4, SLC7A1, SLC8A1, TGFB1, TH, TNF, TRH, TRHR, UCP2, UTS2, UTS2R, VDR, VWF, WNK1, WNK4 | 155 | 99 | 63.87% | 3.47E-10 |
| Alzheimer Disease | A2M, ACE, AD9, APBB2, APOE, APP, BLMH, HFE, MPO, NOS3, PACIP1, PLAU, PSEN1, PSEN2, SORL1 | A2M, ABCA1, ABCA12, ABCA2, ABCB1, ACE, AHSG, APBB1, APBB3, APOA1, APOC1, APOC3, APOD, APOE, APP, BACE1, BCHE, BDNF, CDC2, CHAT, CNTF, COMT, CST3, CTNNA3, CTSD, CYP46, CYP46A1, DAPK1, DLD, DLST, DNMBP, DRD1, DRD3, ESR1, F13A1, FGF1, FYN, GAPDH, GAPDHS, GSK3B, HHEX, HLA-A, HLA-DRB1, HSPA1B, HTR2A, HTR6, IDE, IL18, IL1A, IL1B, IL1RN, IREB2, LDLR, LPA, LPL, LRP1, LRPAP1, LTA, M6PR, MAOA, MAOB, MAPT, MME, MMP3, MPO, MTHFR, MTR, NCSTN, NOS3, NOTCH4, NP, NPY, NQO1, NTF3, OLR1, PARP1, PIN1, PLAU, PNMT, PON1, PON2, POU2F1, PPARA, PRNP, PSEN1, PSEN2, PSENEN, SERPINA3, SLC6A4, SNCA, SOAT1, SOD2, SORL1, STH, TCN1, TCN2, TF, TFAM, TFCP2, TNF, TPH1, UBQLN1, UCHL1, USF1, USF2, VEGFA, VLDLR, WT1 | 108 | 86 | 79.63% | 1.49E-09 |
| Myocardial Infarction | ACE, APOE, ESR1, F7, GCLC, GCLM, LGALS2, LRP8, LTA, MIAT, OLR1, THBD, TNFSF4 | ABCA1, ABCC9, ABO, ACE, ADIPOQ, ADRB1, AGT, AGTR1, AKAP10, ALDH2, ALOX5, ALOX5AP, APOA, APOA1, APOA4, APOA5, APOB, APOE, ARG1, BAT1, CCL11, CCL2, CCR5, CD14, CDKN1B, CDKN1C, CETP, CFH, CRP, CYP11B2, CYP1A2, CYP2C8, CYP2C9, CYP2J2, CYP4A11, ENPP1, ESR1, F12, F13A1, F2, F5, F7, F8, FABP2, FGA, FGB, FGG, GCLM, GJA4, GNB3, GP1BA, HNRPUL1, IGF1, INSR, ITGA2, ITGA2B, ITGB3, ITIH3, LGALS2, LIPG, LPA, LPL, LRPAP1, LTA, MEF2A, MMP3, MT-ND2, MTHFR, NFKBIL1, NOS3, NPR1, OLR1, OR13G1, P2RY11, PAFAH1B1, PAI1, PCSK9, PDX1, PECAM1, PLA2G7, PON1, PON2, PPARG, PTGIS, ROS1, SELE, SELP, SERPINE1, SLC6A4, TAS2R50, TF, TGFB1, THBD, THBS1, THBS4, THPO, TLR4, TNFRSF4, UTS2, VAMP8, VEGFA, WRN | 102 | 75 | 73.53% | 3.40E-10 |
| Asthma | ADRB2, ALOX5, CCL11, CHI3L1, GPR154, HNMT, IL12B, IL13, IRAK3, MUC7, PHF11, PLA2G7, PTGDR, PTGER2, SCGB3A2, TBX21, TNF, UGB | ABO, ADAM33, ADRB2, AICDA, ALOX5, ALOX5AP, AOAH, BAT1, BDKRB2, C3, C5orf20, CCL11, CCL2, CCL5, CCR3, CCR5, CD14, CD40, CHRM1, CHRM3, CLCA1, CMA1, CRHR1, CSF2, CTLA4, CX3CL1, CXCR3, CYP1A1, CYP2J2, CYSLTR1, CYSLTR2, DAP3, DEFB1, DQ1B, EDN1, EGFR, FCER2, FCGR1A, FCGR1B, FCGR2A, FLG, GATA3, GC, GPR1, GPR44, GSTM1, GSTM3, GSTP1, GSTT1, HLA, HLA-DPB1, HLA-DQA1, HLA-DQB1, HLA-DRB1, HNMT, IFNG, IFNGR1, IKBKAP, IL10, IL12B, IL13, IL13RA1, IL15, IL16, IL17F, IL18, IL1A, IL1B, IL1RA, IL1RN, IL3, IL4, IL4R, IL5, IL8, IL8RA, IL8RB, IL9, IRF1, ITGB3, KCNS3, KDR, LELP1, LTA, LTC4S, MIF, MMP9, MS4A1, MS4A2, MT-CO2, MUC7, MYLK, NAT1, NAT2, NOS1, NOS2A, NOS3, PAFAH, PDGFRA, PGDS, PHF11, PTGDR, PTGER2, PTGER3, PTGER4, PTGIR, RNASE3, RUNX1, SCGB1A1, SCGB3A2, SELP, SERPINA3, SERPINE1, SPINK5, SPP1, STAT3, STAT4, STAT6, TAP1, TBX21, TBXA2R, TGFB1, TIMELESS, TLR10, TLR2, TLR4, TNF | 127 | 73 | 57.48% | 1.31E-09 |
| Obesity | ADRB2, ADRB3, AGRP, AKR1C2, CART, ENPP1, FTO, GHRL, LEP, LEPR, MC3R, MC4R, NR0B2, NTRK2, PCSK1, POMC, PPARG, PPARGC1B, SDC3, SIM1, SLC6A14, UCP1, UCP2, UCP3 | A2M, ABCG8, ADIPOQ, ADRA2A, ADRA2B, ADRB1, ADRB2, ADRB3, AHSG, APOA4, APOA5, APOB, APOC3, AR, CAPN10, CART, CCK, CIDEA, CNR1, CPT1A, CPT1B, DBP, DRD4, ENPP1, FAAH, FABP2, FAS, FTO, GABRA6, GAD2, GHRL, GPR10, GPR50, HSPA1B, HTR2A, HTR2C, IGF2, IL6, IL6R, INS, INSIG2, LDLR, LEP, LEPR, LIPC, LIPE, LPL, LRP5, MAOA, MAOB, MC3R, MC4R, MCHR1, MIF, NMU, NPY, NPY2R, NPY5R, NR3C1, PCSK1, PLA2G7, PLIN, PLTP, PMCH, POMC, PPARG, PPARGC1A, PRLHR, PTPN1, PYY, RETN, SCG3, SDC3, SELE, SERPINA6, SERPINE1, SH2B1, SLC6A14, SLC6A4, STCH, TH, TNF, TNFRSF1A, UCP1, UCP2, UCP3, USF1 | 87 | 68 | 78.16% | 1.47E-10 |
| Arthritis | ASPN, CD244, FRZB, HLA-DR1B, IL10, IL6, LTA, MATN3, MHC2TA, MIF, NFKBIL1, NOD2, PADI4, PSTPIP1, PTPN22, RUNX1, SLC22A4, STAT4 | A2M, ABCA7, ABCB1, AMPD1, ATIC, BAT2, BTLA, CARD15, CCL26, CCR5, CD19, CD22, CIITA, CTLA4, CYP11B2, CYP17A1, CYP1A1, ESR1, EXOC4, FAS, FCGR2A, FCGR2B, FCGR3A, FCRL3, HCR, HLA, HLA-B, HLA-C, HLA-DMA, HLA-DQA1, HLA-DQB1, HLA-DRB1, HMHA1, ICAM1, IFNG, IGLV8, IGLV@, IL10, IL12B, IL13, IL18, IL1A, IL1B, IL1RN, IL2, IL23A, IL3, IL4, IL4R, IL6, IRF5, ITPA, KIR2DL1, KIR2DS2, KLRC1, KLRC2, KLRD1, LTA, MBL2, MEFV, MICA, MICB, MIF, MMP1, MMP12, MMP13, MMP2, MMP3, MMP7, MTHFR, MTR, MYO9B, NAT2, NFKB1, NLRP3, NOS2A, PADI4, PARP1, PDCD1, PLAU, PRKCH, PSORS1C1, PTPN22, RAGE, SAA1, SERPINA3, SH2D2A, SLC11A1, SLC19A1, SLC22A4, SOD2, SPP1, TAP1, TAP2, TIMELESS, TNF, TNFRSF1A, TNFRSF1B, TNFSF13B, TP53, TRAF5, TRB@, VEGFA | 103 | 59 | 57.28% | 1.31E-09 |
| Coronary Artery Disease | ABCA1, CCL2, CX3CR1, IRS1, KL, LRP6, MEF2A, PON1, PON2 | ABCA1, ABCC6, ACE, ADA, ADH1B, ADIPOQ, ADIPOR1, AGT, AGTR1, ALOX15, APOA1, APOA2, APOA4, APOA5, APOB, APOC3, APOE, CCL5, CCR2, CD14, CD36, CD40, CDKN2A, CDKN2B, CETP, CMA1, CRP, CX3CR1, CYBA, CYP3A4, CYP7A1, ECE1, EDN1, EPHX2, F12, F5, F7, FBN1, FCGR3A, GATA2, GCLM, GJA4, GNB3, GPX1, HLA-DQB1, HLA-DRB1, HMOX1, ICAM1, IL1RN, IL6, IRAK1, ITGA2, ITGB3, LCAT, LIPC, LPA, LPL, LTA, MBL2, MC4R, MEF2A, MMP3, MMP7, MMP9, MPO, MTHFR, NOS3, NR3C1, OLR1, PECAM1, PLA2G7, PON1, PON2, PPARA, RAGE, SELE, SELP, SELPLG, THBS1, THBS2, TLR4, TNFAIP3, VEGFA, WRN | 84 | 58 | 69.05% | 4.29E-10 |
| Schizophrenia | CHI3L1, CLINT1, COMT, DISC1, DRD3, HTR2A, MTHFR, PRODH, RGS4, RTN4R, SYN2, TAAR6 | ABCB1, AKT1, ATXN8OS, BDNF, CCK, CCKAR, CHGB, CHL1, CHRM1, CHRNA7, CLOCK, CNR1, CNTF, COMT, DBH, DRD2, DRD3, DRD4, DRD5, DRP2, DTNBP1, EGF, FRK, GABBR1, GJA8, GRIA4, GRIN2A, GRIN2B, GRM3, GRM5, HLA-A, HLA-DQB1, HLA-DRB1, HMBS, HTR2A, HTR2C, HTR4, HTR5A, IL10, IL10RA, IL1B, JARID2, KCNN3, L1CAM, MAOA, MED12, MTHFR, NDUFV2, NOS1, NOTCH4, NQO2, NR4A2, NRG1, NTF3, OPRS1, PDE4B, PDLIM5, PIP5K2A, PLA2G1B, PON1, PPP3CC, RGS2, SLC6A3, SLC6A4, SRR, SYN3, TH, TNF, TPH1, YWHAH, ZDHHC8 | 71 | 47 | 66.20% | 7.24E-10 |
| Leukemia | ABL1, ACSL6, AF10, AF1Q, ARHGAP26, ARHGEF12, ARNT, ATM, BAX, BCL2, BCR, BRCA2, CALM, CBFB, CEBPA, CHIC2, ETV6, FLT3, GATA1, GMPS, HOXD4, IKZF1, IRF1, JAK2, KIT, KRAS2, LPP, MKL1, MYB, NBS1, NF1, NPM1, NQO1, NUMA1, NUP214, PDGFRB, PML, PTPN11, RAP1GDS1, RUNX1, SETBP1, STAT5B, TAL1, TAL2, TCRA, WHSC1L1, ZBTB16, ZNF198 | ABCB1, ABL1, BAX, BCL10, BCL2, CDKN2A, CTLA4, CXCL12, CYP1A1, CYP3A4, DCK, DHFR, FAS, FLT3, GATA1, GNAS, GSTM1, GSTP1, GSTT1, HFE, HLA-A, HLA-B, HLA-DPB1, HPSE, HY, IL10, KRAS, MLH1, MTHFR, MTRR, NOTCH1, NPM1, NQO1, NR3C1, NRAS, PDCD5, PIGA, PTPN11, RUNX1, SHMT1, SLC19A1, SOD2, TNF, TP53, TP73L, TPMT, TYMS, UGT1A1, VDR | 49 | 38 | 77.55% | 4.25E-07 |
| Adenocarcinoma | BRAF, EGFR, ERBB2, PRKN, RAD54B, RAD54L | APC, ATM, BRAF, CCKAR, CCND1, CDH1, CYP11A1, CYP17A1, CYP1A1, CYP1A2, CYP1B1, CYP2A13, CYP2A6, EGFR, ELAC2, EPHX1, ERBB2, ERCC2, FANCC, GSTM1, GSTP1, GSTT1, HLA-DQB1, HLA-DRB1, HMOX1, HRAS, IL1B, IL1RN, KLK2, KRAS, LAPTM4B, LIG4, LRMP, MC1R, MDM2, MMP1, MMP2, MMP3, MMP9, MTHFR, MTRR, MUC1, NAT1, NQO1, OGG1, PDCD5, PGR, RASSF1, SERPINE1, SFN, SHMT1, SOD2, SRD5A2, STK11, SULT1A1, TLR4, TP53, TP73, UGT1A7, XPA, XPC, XRCC1 | 62 | 38 | 61.29% | 9.55E-06 |
| Parkinson Disease | ADH1C, ATP13A2, DBH, DJ1, DRD4, FGF20, GIGYF2, HTRA2, LRRK2, MAPT, NDUFV2, NR4A2, PINK1, PRKN, SNCA, SNCAIP, TBP, UCHL1 | A2M, ACE, ADH1C, ADH4, APOE, BDNF, CCK, CCKBR, CD14, COMT, CTSD, CYP1A1, CYP27A1, CYP2D6, DRD2, ESR2, FGF20, FRAXA, FRAXE, GBA, GSTM1, GSTO1, GSTO2, GSTP1, HTR6, IL1B, IL6, LRRK2, MAOA, MAOB, MAPT, MT-ND1, MT-ND3, NOS1, NOS2A, NQO1, NQO2, NR4A2, OPRM1, PARK2, PARP1, PINK1, PON1, SLC6A3, SLC6A4, SNCA, SOD2, TFAM, UCHL1 | 49 | 38 | 77.55% | 8.53E-10 |
| Hepatitis | CCR5, CRFB4, IFNAR2, IFNG | ABCC2, ADA, C4A, CCL2, CCL5, CCR2, CCR5, CD81, CPT1A, CR1, CTLA4, CYP1A1, CYP1A2, CYP2C8, CYP2D6, CYP2E1, CYP3A4, DDX5, EDN1, EIF2AK2, ERCC2, FAS, GSTM1, HAVCR1, HBB, HFE, HLA, HLA-A, HLA-B, HLA-DQA1, HLA-DQB1, HLA-DRB1, HP, HTR1A, IFNA2, IFNAR1, IFNG, IGF2, IL10, IL10RA, IL12B, IL16, IL18, IL1B, IL1RN, IL6, IRF1, KIR3DL1, LDLR, LEC, LTA, MDM2, MMP1, MPO, MTP, MX1, NAT1, NS5A, OAS1, POLG2, PON1, PSMB8, PTK2, SOD2, TAP2, TBX21, TGFB1, TLR7, TNF, TP53, UGT1A7, UGT2B7, VDR | 73 | 35 | 47.95% | 1.77E-09 |
| Insulin Resistance | PPARG, PPP1R3A, PTPN1, RETN | ABCG5, ABCG8, ACE, ADIPOQ, ADIPOR2, ADRA2A, ADRB3, AMPD1, APOA5, CAPN10, CCL2, CD36, CYP1A2, DIO2, ENPP1, FABP2, FABP4, FFAR1, FOXC2, GC, GCGR, GFPT1, HSD11B1, ICAM1, IGF1R, IL6R, INS, INSR, IRS1, LEP, LEPR, LIPC, LPL, MC4R, NOS3, PLIN, PPARD, PPARG, PPARGC1A, RETN, SCARB1, TCF1, TNF, TSHR, UCP1, USF1 | 46 | 30 | 65.22% | 1.84E-09 |
| Osteoporosis | CALCA, CALCR, COL1A1, COL1A2, LRP5, RIL, SLC34A1, SLC9A3R1 | AHSG, ALDH2, ALOX12, ALPL, AR, BGLAP, CALCA, CALCR, CASR, CAT, CCL2, CD38, CNR1, COL1A1, CYP17A1, CYP19A1, CYP3A7, DBP, ESR1, ESR2, FLT3, FOXC2, GC, GH1, GNRH1, IGF1, IL10, IL1B, IL1R, IL1R1, IL1RN, IL6, KIT, KL, LEPR, LRP5, MTHFR, NOS3, NPPB, PLIN, PLXNA2, PON1, PON2, PTHR1, RIL, TGFB1, TNF, TNFR2, TNFRSF11B, TNFRSF1B, VDR, WRN | 52 | 26 | 50.00% | 2.92E-05 |
| Adenoma | AIP, APC, GNAI2, GNAS, HMGA2, HNF1A, HRPT2, LHCGR, MEN1, MUTYH, ODC1, PLAG1, THRA, TSHR | ALOX12, ALOX5, APC, AR, CASR, CCND1, CYP1A2, CYP1B1, CYP2C9, EPHX1, KRAS, MMP1, MMP3, MTHFR, MUTYH, MYH1, NAT2, NPPA, NQO1, ODC1, OGG1, PDE11A, PPARD, PTGS1, PTPRJ, RAS, REN, RET, SLC10A2, TCF1, UGT1A6, XPC | 32 | 22 | 68.75% | 8.70E-05 |
| Psoriasis | HLA-C, IL23R, PSORS10, PSORS6 | ATIC, CDSN, HCR, HLA, HLA-A, HLA-C, HLA-DMB, HLA-DQA1, HLA-DQB1, HLA-DRB1, IL10, IL12B, IL15, IL19, IL1RN, IL20, IL23R, IL24, IRF2, KIR2DL2, KIR2DS1, LTA, MICB, MIF, MMP2, PSMB8, PSMB9, PSORS1C3, RAGE, SERPINA1, SLC19A1, SLC9A3R1, TAP1, TAP2, TNF, TYMS, VDR, VEGFA | 38 | 21 | 55.26% | 1.48E-09 |
| Melanoma | BRAF, CDK4, CDKN2A, MC1R, STK11, XRCC3 | APEX1, ASIP, BRAF, CCR2, CD86, CDKN2A, EGF, FAS, FASLG, FGFR4, HMOX1, IL24, MC1R, NFKB1, NFKBIA, NRAS, RAS, TGFA, TP53, VDR, VEGFA, XPC, XRCC1, XRCC3 | 24 | 21 | 87.50% | 5.31E-09 |
| Multiple Sclerosis | CD24, HLA-DQB1, HLA-DR1B, IL7R, MHC2TA, PTPRC | ADAMTS14, ADRB2, APOC2, APOE, BTNL2, CCR2, CCR5, CD24, CNTF, CRYAB, CTLA4, FAS, FASLG, FCRL3, GABRA3, HLA-DPB1, HLA-DQA1, HLA-DQB1, HLA-DRB1, IFNG, IL1B, IL1RN, IL2, IL4, IL4R, IL6, JAG1, LILRA3, MBP, MEFV, MPO, OAS1, PDCD1, PNMT, POU2AF1, PRKCA, PTAFR, PTPRC, PVRL2, SH2D2A, TAC1, TNF, UCP2, VDR | 44 | 20 | 45.45% | 1.72E-09 |
| Malaria | CD36, CR1, FCGR2B, FY, GYPA, GYPC, ICAM1, NCR3, NOS2A, SLC4A1, TIRAP, TNF | CD36, CR1, FCGR2A, FCGR3A, FCGR3B, G6PD, HLA-A, HLA-DRB5, HMOX1, Hb, ICAM1, IFNA1, IFNAR1, IFNGR1, IL13, IL1A, IL1RN, IL4, MBL2, NOS2, NOS2A, PECAM1, PSMB9, TNF, TNFAIP1 | 25 | 19 | 76.00% | 1.27E-07 |
| Epilepsy | ALDH7A1, ARHGEF9, ARX, ATP6AP2, CACNA1H, CACNB4, CHRNA2, CHRNA4, CHRNB2, CLCN2, CLN8, CNTNAP2, CSTB, EFHC1, EPM2A, GABRA1, GABRB3, GABRG2, JRK, KCNMA1, KCNQ2, KCNQ3, KCTD7, LGI1, LYK5, ME2, NHLRC1, OPRM1, PCDH19, POLG, PRICKLE1, SCN1A, SCN1B, SIAT9, SLC25A22, SRPX2, SYN1 | ABCB1, APOE, ATP1A2, CACNA1A, CACNA1H, CACNG3, CHRNA4, CLCN2, CYP2C19, CYP2C9, FABP2, GABRB1, GABRG2, GRIK1, HP, KCNJ10, KCNJ3, LGI4, OPRM1, PDYN, PRNP, SCN1A, SCN1B, SLC4A3 | 24 | 18 | 75.00% | 1.12E-05 |
| Tuberculosis | CCL2, CD209, IFNG, IFNGR1, NRAMP1, SP110, TIRAP | CCL2, CYP2E1, GSTM1, GSTT1, HLA, HLA-DQA1, HLA-DQB1, HLA-DRB1, HLA-DRB3, HLA-DRB5, IFNG, IFNGR1, IL10, IL12B, IL12RB1, IL4, IL8, IL8RA, IL8RB, NOS2A, P2RX7, SFTPA2, SLC11A1, TIRAP, TLR2, TNF, TNFRSF1A, VDR | 28 | 17 | 60.71% | 3.52E-05 |
| Dementia | CHMP2B, DJ1, ITM2B, MAPT, PSEN1, SNCA, SNCB, TNF, TRPM7, VCP | AGT, APBB1, APOE, AVP, CDC2, HSPA1A, ICAM1, IGF1R, IL1B, MAPT, MMP1, MMP3, MMP9, PON2, PRNP, PSEN1, S100B, SERPINI1, TNF, VLDLR | 20 | 17 | 85.00% | 4.57E-07 |
| Hypercholesterolemia | ABCA1, APOA2, APOB, EPHX2, GSBS, ITIH4, LDLR, LDLRAP1, PCSK9 | ABCA1, ABCB1, ABCG5, ABCG8, APOA1, APOB, APOC1, APOE, CYP2D6, CYP7A1, ITIH4, LDLR, LPL, NPC1L1, PCSK9, PON1, SREBF2, UCP1 | 18 | 16 | 88.89% | 3.62E-08 |
| Lymphoma | ATM, BCL10, BCL2, BCL7A, BCL8, CASP10, MAD1L1, MALT1, MYC, PRF1, RAD54B, RAD54L, TCRA, ZNF198 | ABCG2, ATM, BCL1, BCL2, BCL6, CTLA4, CXCL12, CYP17A1, CYP1A1, CYP2B6, EDN1, FCGR3A, GHRL, GSTM1, GSTT1, HLA-DQB1, HLA-DRB1, IL1B, MSH2, MTHFR, PON1, PRF1, TNFRSF1A, TP53 | 24 | 16 | 66.67% | 0.004995278 |
| Anemia | ABCB7, AK1, ALAS2, AMN, BAAT, BPGM, BRCA2, BRIP1, CDAN1, CUBN, EPHX1, FAAP95, FANCA, FANCC, FANCD2, FANCE, FANCF, FANCI, FANCM, G6PD, GATA1, GCLC, GLRX5, GPI, GSS, HBA1, HBA2, HBB, HK1, IFNG, IRF1, MTRR, NRAMP2, NT5C3, PALB2, PHF9, PUS1, RHAG, RPL11, RPL35A, RPL5, RPS17, RPS19, RPS24, RPS7, SLC19A2, SLC2A1, SLC4A1, SPTB, TERC, TERT, TF, TJP2, TMPRSS6, TPI1, XRCC9 | AK1, CYP1A1, CYP2E1, Cdc25A, EPB42, G6PD, GATA1, HLA, IFNG, NOS3, PKLR, TERC, TERF1, TERF2, TERT, TP53, UGT1A1 | 17 | 15 | 88.24% | 2.17E-05 |
| Macular Degeneration | ABCA4, APOE, C2, C3, CFB, CNGB3, CST3, CX3CR1, ERCC6, FBLN5, HF1, HMCN1, HTRA1, LOC387715, RAXL1, RPGR, TLR3, TLR4 | ABCA1, ABCA4, APOE, ARMS2, C2, C7, CFB, CFH, CFHR1, CFHR3, CST3, CYP1A1, ERCC6, HTR1A, HTRA1, MBL2, MMP9, PON1, RDS, VEGFA | 20 | 15 | 75.00% | 2.57E-05 |
| Crohn Disease | DLG5, IL23R, IL6, NOD2, SLC22A4 | ABCB1, ATG16L1, CARD15, CCL2, CCR2, CD14, CRP, DLG5, FCGR3A, HLA, HLA-DRA, HLA-DRB1, HSPA2, IBD5, IL16, IL23R, IL4, IL4R, MBL2, MIF, NKX2-3, NOD2, NRAMP, PTPN2, SERPINE1, SLC22A4, SLC22A5, TNF | 28 | 14 | 50.00% | 5.65E-04 |
| Celiac Disease | CTLA4, HLA-DQA1, HLA-DQB1, MYO9B, SH2B3 | CTLA4, DM, FAS, HLA, HLA-B, HLA-D, HLA-DPB1, HLA-DQA1, HLA-DQB1, HLA-DRB1, ICAM1, ICOS, IFNG, IL2, IL21, INS, KIR2DL5B, MBL2, MICA, MMP3, TIPARP, TNF | 22 | 13 | 59.09% | 2.17E-07 |
| Pancreatitis | CFTR, CTRC, PRSS1, PRSS2, SPINK1 | ALDH2, CAT, CCL2, CEL, CFTR, CTSB, GSTT1, HLA, HLA-A, HLA-DQB1, HLA-DRB1, HSPA1B, IL1RN, IL8, KRT8, MIF, PRSS1, SPINK1, TGFB1, TNF | 20 | 11 | 55.00% | 1.13E-05 |
| Malaria, Cerebral | CD36, ICAM1, TNF | CD36, CR1, FCGR2A, FCGR3A, FCGR3B, HLA-DRB5, HMOX1, IFNA1, IFNAR1, IFNGR1, IL4, NOS2, NOS2A, PECAM1, TNF, TNFAIP1 | 16 | 11 | 68.75% | 1.09E-05 |
| Glaucoma | CYP1B1, FOXC1, LMX1B, MYOC, OPA1, OPTN, WDR36 | APOE, CYP1B1, EDNRA, GSTM1, IGF2, IL1B, IL1RN, MMP9, MYOC, OPA1, OPTC, OPTN, PON1, TAP1 | 14 | 10 | 71.43% | 5.82E-05 |
| Cardiomyopathy, Dilated | ABCC9, ACTC1, ACTN2, CSRP3, DES, DMD, EYA4, FKTN, LDB3, LMNA, MYBPC3, MYH7, PLN, PSEN1, PSEN2, SCN5A, SGCD, TAZ, TCAP, TMPO, TNNC1, TNNI3, TNNT2, TPM1, TTN, VCL | ACE, ADRA2C, ADRB1, ADRB2, CYP11B2, DMD, HLA-DPA1, HLA-DPB1, HLA-DQA1, HLA-DRB1, LMNA, MYBPC3, NEBL, PLN, TNNT2 | 15 | 9 | 60.00% | 0.03128686 |
| Retinitis Pigmentosa | ABCA4, AIPL1, CA4, CERKL, CNGA1, CNGB1, CRB1, CRX, EYS, FSCN2, HPRP3, IDH3B, IMPDH1, MERTK, MFRP, NR2E3, NRL, PDE6A, PDE6B, PRCD, PROM1, PRPF31, PRPF8, RDS, RGR, RHO, RLBP1, ROM1, RP1, RP2, RP9, RPE65, RPGR, SEMA4A, TOPORS, TULP1, USH2A | CRX, FSCN2, NRL, PDE6B, RHO, ROM1, RP1, RPGR, RPGRIP1 | 9 | 9 | 100.00% | 3.09E-05 |
| Hypertriglyceridemia | APOA5, LIPI, RP1 | APOA2, APOA4, APOA5, APOC2, APOC3, APOE, CYP7A1, LPL, PPARA, RP1 | 10 | 8 | 80.00% | 3.07E-07 |
| Deafness | ACTG1, ATP2B2, ATP6B1, CCDC50, CD151, CDH23, CLDN14, COCH, COL11A2, COL2A1, CRYM, DFNA5, DIAPH1, DSPP, ESPN, ESRRB, EYA4, FGF3, GATA3, GJB2, GJB3, GJB6, IGF1, JAG1, KCNQ4, KIAA1199, LHFPL5, LRTOMT, MARVELD2, MYH14, MYH9, MYO15A, MYO1A, MYO3A, MYO6, MYO7A, OPA1, OTOA, OTOF, PAX3, PCDH15, PJVK, POU3F4, POU4F3, PRES, RDX, RPGR, SDHD, SIX1, SLC17A8, SLC4A11, STRC, TECTA, TFCP2L3, TIMM8A, TMC1, TMIE, TMPRSS3, TRIOBP, TRMU, USH1C, WHRN | GJA1, GJB2, MYO15, MYO7A, NDP, POU3F4, SLC26A4, TECTA, TMPRSS3 | 9 | 7 | 77.78% | 0.018259674 |
| Thrombosis | CBS, FGA, SERPINA10 | F2, F5, ITGA2B, PROC, PROS1, PTGER2, SELPLG, SERPINC1, VKORC1 | 9 | 7 | 77.78% | 3.36E-04 |
| Amyotrophic Lateral Sclerosis | ALS2, ANG, CHMP2B, DCTN1, DJ1, NEFH, PRPH, SETX, SOD1, TARDBP, TRPM7, VAPB | MT-ND5, MT2A, MT3, OGG1, PON1, PON2, PON3, PRPH, SMN1, SOD1, VEGFA | 11 | 6 | 54.55% | 0.087513613 |
| Infection | CCR2, CCR5, CX3CR1, FCGR3A, FUT2, IFNGR1, IFNGR2, IL12RB1, IL1B, IL1RN, MBL2, MYD88, NRAMP1, OAS1, RAG1, RPGR, STAT1, STAT3, TCBA1 | FCGR2A, HLA, IL18, IL6, MBL2, NOD2, TGFB1 | 7 | 6 | 85.71% | 0.005321612 |
| Mental Retardation | ACSL4, AGTR2, AP1S2, ARHGEF6, ARX, ATP6AP2, ATRX, BRWD3, CASK, CC2D1A, CDH15, CRBN, CUL4B, DIP2B, DLG3, DOCK8, FGD1, FMR2, FTSJ1, GDI1, GNAS, GRIA3, GRIK2, HSD17B10, HUWE1, IGBP1, IGF1, IL1RAPL1, KCNK9, KIRREL3, MAGT1, MBD5, MCPH1, MECP2, NLGN4, OPHN1, PAK3, PCDH19, PHF8, POMT1, PRSS12, RPS6KA3, SATB2, SHROOM4, SLC9A6, SMCX, SMS, SOX3, SRPX2, SYNGAP, TM4SF2, TUSC3, UBE2A, UPF3B, VLDLR, ZBTB16, ZDHHC15, ZDHHC9, ZNF41, ZNF674, ZNF81 | DIO2, FMR1, HRAS, MECP2, POU1F1, RPS6KA3, THRB | 7 | 6 | 85.71% | 0.014492311 |
| Cardiomyopathy, Hypertrophic | COX15, MYL2, MYLK2, PRKAG2 | AGT, CYP11B2, HLA-B, MYBPC3, MYH7, MYL2, MYOZ2, NOS3, PLA2G7, PLN, SCO2, TNNT2 | 12 | 6 | 50.00% | 0.015321372 |
| Charcot-Marie-Tooth Disease | DNM2, EGR2, FGD4, FIG4, GARS, GDAP1, HOXD10, HSPB1, HSPB8, KIF1B, LITAF, LMNA, MFN2, MPZ, MTMR2, NDRG1, NEFL, PMP22, PRPS1, RAB7, SBF2, SH3TC2, YARS | EGR2, GJB1, MPZ, MTMR2, PMP22 | 5 | 5 | 100.00% | 0.005294172 |
| Neural Tube Defects | MTHFR, MTR, MTRR, T, VANGL1 | BHMT, MTHFD1, MTHFR, MTR, MTRR, PDGFRA, SLC19A1, ZIC2 | 8 | 5 | 62.50% | 0.001610913 |
| Leprosy | LTA, PRKN, TLR1, TLR2 | HLA, HLA-A, IL12B, LAMA2, PACRG, PARK2, TLR2, TNF, VDR | 9 | 5 | 55.56% | 1.68E-04 |
| Sarcoidosis | BTNL2, HLA-DR1B, NOD2 | ACE, BTNL2, CD14, CR1, HLA-DQB1, HLA-DRB1, HLA-DRB3, HLA-G, HSPA1L, IFNA17, MIF, MUC1, NFKB1, NOD2, SELE, TLR2, TLR4, VEGFA | 18 | 5 | 27.78% | 0.025411043 |
| Cerebellar Ataxia | ATXN1, ATXN10, ATXN2, ATXN7, C10orf2, CABC1, CACNA1A, CP, FGF14, ITPR1, KCNC3, PLEKHG4, POLG, PPP2R2B, PRKCG, SCA25, SCA8, SPTBN2, SYNE1, TBP, TDP1, TTBK2 | CACNA1A, CEP290, CP, NPHP1 | 4 | 4 | 100.00% | 0.017623831 |
| Long QT Syndrome | AKAP9, ALG10, ANK2, CAV3, KCNE1, KCNE2, KCNH2, KCNJ2, KCNQ1, SCN4B, SCN5A | CYP1A2, CYP2C9, HERG, KCNE1, KCNH2, KCNQ1, SCN5A | 7 | 4 | 57.14% | 0.043882104 |
| Lymphoma, Non-Hodgkin | PRF1, RAD54B, RAD54L | CTLA4, CYP17A1, CYP1A1, GHRL, GSTM1, GSTT1, MSH2, MTHFR, PON1, TP53 | 10 | 4 | 40.00% | 0.078570854 |
| Osteoarthritis | ASPN, FRZB, MATN3 | AGC1, AR, ASPN, CALM1, COL1A1, COL2A1, COL9A1, COMP, ENPP1, ESR1, FRZB, GDF5, HAPLN1, HLA-DQA1, IL1A, IL4R, KL, MATN1, MBOAT5, MMP2, VDR | 21 | 4 | 19.05% | 5.67E-11 |
| Hemochromatosis | BMP2, HAMP, HFE, HJV, SLC40A1, TFR2 | CYBRD1, HAMP, HFE, HFE2, HLA, SLC40A1, TFR2 | 7 | 4 | 57.14% | 2.84E-05 |
| Glioblastoma | BRCA2, DMBT1, ERBB2, LGI1, PPARG | EGFR, MGMT, MMP1, MTR, PTEN | 5 | 4 | 80.00% | 0.015121965 |
| Hypospadias | AR, MAMLD1, SRD5A2 | AR, ESR2, INSL3, SRD5A2, WT1 | 5 | 4 | 80.00% | 0.014572726 |
| Xeroderma Pigmentosum | DDB2, ERCC2, ERCC3, ERCC4, ERCC5, POLH, XPA, XPC | ERCC2, ERCC3, ERCC6, XPA | 4 | 4 | 100.00% | 0.006989059 |
| Epilepsy, Generalized | CACNB4, GABRG2, SCN1A | CACNA1A, CHRNA4, CLCN2, GABRG2, HP, KCNJ10, KCNJ3, OPRM1, SCN1B, SLC4A3 | 10 | 4 | 40.00% | 0.002062655 |
| Pheochromocytoma | GDNF, KIF1B, RET, SDHB, SDHD, VHL | GRIN2B, RET, SDHB, VHL | 4 | 4 | 100.00% | 0.007766857 |
| Cleft Palate | CDH1, HOXA2, L1CAM, SATB2, TBX22 | BCL3, COMT, CYP1A1, GAD1, GSTM1, SLC19A1, TGFA, TGFB3 | 8 | 4 | 50.00% | 0.019293958 |
| Huntington Disease | HD, JPH3, PRNP, TBP | CYP2D6, CYP2E1, ERDA1, GRIK1, GRIK2, HD, NAT2, NQO1, PRNP, UCHL1 | 10 | 4 | 40.00% | 0.226841999 |
| Dystonia | ACTB, ATP1A3, DRD2, DRD5, DYT1, FA2H, GCH1, PRKRA, SCP2, SGCE, SLC2A1, TAF1 | DRD2, DRD5, GCH1, TOR1A | 4 | 3 | 75.00% | 0.225182188 |
| Bernard-Soulier Syndrome | GP1BA, GP1BB, GP9 | GP1BA, GP1BB, GP9 | 3 | 3 | 100.00% | 1.27E-04 |
| Sarcoma | ASPSCR1, CHEK2, CSMF, DDIT3, EWSR1, EXT1, FOXO1A, IL6, MXI1, PAX3, PAX7, PDGFB, SDHB, SDHC, SDHD, SLC22A1L, SSX1, SSX2, TP53 | FGFR4, IL13, IL8RB, TP53 | 4 | 3 | 75.00% | 0.138099019 |
| Hypothyroidism | CTLA4, DUOX2, GLIS3, NKX2-1, NKX2E, PAX8, SLC5A5, TG, TSHR | CTLA4, CYP2D6, MED12, TPO | 4 | 3 | 75.00% | 0.02079301 |
| Atrophy | AR, ATN1, CRB1, FHL1, HEXB, OAT, OPA1, OPA3, PLEKHG5, SMN1, TEAD1, TLR3, UBE1, VAPB | BDNF, PCM1, PRNP | 3 | 3 | 100.00% | 0.049898081 |
| Cholestasis | ABCB11, ABCB4, ATP8B1, HSD3B7 | ABCB11, ABCB4, ACE, ATP8B1, HLA-DPA1 | 5 | 3 | 60.00% | 0.01020875 |
| Neuroblastoma | ALK, KIF1B, NME1, PMX2B | HRAS, MAPK8IP1, MYCN, PHOX2B, SSTR2 | 5 | 3 | 60.00% | 0.084269558 |
| Atrial Fibrillation | GJA5, KCNA5, KCNE2, KCNQ1, NPPA | ACE, CETP, F13A1, GJA5, KCNE1, MINK1, NOS3, SCN5A | 8 | 3 | 37.50% | 0.103695985 |
| Thrombocytopenia | CYCS, FLJ14813, GATA1, HOXA11, MPL, MYH9, WAS | FAS, ITGB3, WAS | 3 | 3 | 100.00% | 0.019567607 |
| Cataract | BEST1, BFSP1, BFSP2, CHMP4B, CRYAA, CRYAB, CRYBA1, CRYBA4, CRYBB1, CRYBB2, CRYBB3, CRYGC, CRYGD, CRYGS, CTDP1, EYA1, FTL, GALK1, GCNT2, GJA3, GJA8, HSF4, LIM2, MAF, MIP, OPA3, PAX6, PITX3, SIX6, SLC16A12 | CRYGC, GCNT2, IFNGR1 | 3 | 3 | 100.00% | 0.021034811 |
| Glioma | LRP5, PTEN, SDHB, SDHC, SDHD | CDKN2A, CYP2E1, EGF, TP53 | 4 | 3 | 75.00% | 0.051702231 |
| Obsessive-Compulsive Disorder | BDNF, HTR2A, SLC6A4 | BDNF, COMT, DRD4, HTR1B, HTR2A, NRCAM, SLC6A4 | 7 | 3 | 42.86% | 0.075851181 |
| Thrombophilia | F5, FGB, FGG, HCF2, HRG, PROC, PROS1, THBD | AGT, F2, F5 | 3 | 3 | 100.00% | 5.49E-05 |
| Neurofibroma | NF1, NF2, SPRED1 | CDKN2A, NF1, NF2 | 3 | 3 | 100.00% | 0.011038989 |
| Dwarfism | GHR, GHRHR, PCNT2 | GHR, IGF1 | 2 | 2 | 100.00% | 0.004583984 |
| Alopecia | CLDN1, FOXN1, HR, RBM28 | AR, HLA-A, IL1RN, MIF, PTPN22 | 5 | 2 | 40.00% | 0.120861013 |
| Ehlers-danlos Syndrome | ADAMTS2, B4GALT7, COL1A1, COL1A2, COL3A1, COL5A1, COL5A2, PLOD, SLC39A13, TNXB | COL3A1, PLOD1, TNXB | 3 | 2 | 66.67% | 0.030847302 |
| Osteogenesis Imperfecta | COL1A1, COL1A2, CRTAP, LEPRE1 | COL1A1, COL1A2 | 2 | 2 | 100.00% | 0.030537742 |
| Hyperparathyroidism | CASR, HRPT2, MEN1 | CASR, MEN1, PTH | 3 | 2 | 66.67% | 0.11810455 |
| Pseudohermaphroditism | CYP19A1, HSD17B3, LHCGR | AR, CYP19A1, WT1 | 3 | 2 | 66.67% | 0.002705237 |
| Seizures | CLCN2, GABRG2, SCN1A, SCN1B, SCN2A1 | CHRNA4, CSNK1G2, GABRG2, HCN2, KCNJ10, SCN2A, SLC6A3 | 7 | 2 | 28.57% | 0.113865362 |
| Cystinuria | C2orf25, CBS, LMBRD1, MMACHC, MTHFR, MTRR, SLC3A1, SLC7A9 | SLC3A1, SLC7A9 | 2 | 2 | 100.00% | 0.036833594 |
| Leiomyoma | COL4A6, FH, HMGA2, TSC1, TSC2 | ANGPT2, CYP17A1, CYP19A1, CYP1A1, CYP2A13, ESR1, IL12RB1, NR3C1 | 8 | 2 | 25.00% | 0.748068827 |
| Carcinoma, Papillary | GOLGA5, MET, NCOA4, PCM1, PRCC, PRKAR1A, TFE3, TRIM24, TRIM33 | BRAF, RET | 2 | 2 | 100.00% | 0.11807806 |
| Hirschsprung Disease | ECE1, EDN3, EDNRB, GDNF, L1CAM, PMX2B, RET | EDNRB, PHOX2B, RET | 3 | 2 | 66.67% | 0.02911582 |
| Paraganglioma | SDHB, SDHC, SDHD | SDHB, SDHD | 2 | 2 | 100.00% | 0.047547852 |
| Wilms Tumor | BRCA2, GPC3, POU6F2, WT1 | POU6F2, WT1 | 2 | 2 | 100.00% | 0.036027599 |
| Pseudoxanthoma Elasticum | ABCC6, GGCX, XYLT1, XYLT2 | ABCC6, SPP1 | 2 | 2 | 100.00% | 0.010291722 |
| Bardet-Biedl Syndrome | ARL6, BBS1, BBS12, BBS2, BBS4, BBS5, BBS7, CEP290, MKKS, MKS1, PTHB1, TMEM67, TRIM32, TTC8 | BBS2, BBS4, NMB | 3 | 2 | 66.67% | 0.140010073 |
| Leiomyomatosis | COL4A6, FH, TSC1, TSC2 | CYP17A1, CYP19A1, CYP1A1, CYP2A13, ESR1 | 5 | 1 | 20.00% | 0.82581653 |
| Noonan Syndrome | KRAS2, NF1, PTPN11, RAF1, SOS1 | PTPN11 | 1 | 1 | 100.00% | 0.319217592 |
| Eclampsia | AGT, EPHX1, STOX1 | MTHFR | 1 | 1 | 100.00% | 0.117719704 |
| Hemangioma, Capillary | ANTXR1, FLT4, KDR | VHL | 1 | 1 | 100.00% | 0.072583858 |
| Homocystinuria | C2orf25, CBS, LMBRD1, MMACHC, MTHFR, MTRR | CBS | 1 | 1 | 100.00% | 0.185363716 |
| Gout | HPRT1, PRPS1, RNASEH2A, RNASEH2B, RNASEH2C, TREX1 | APOE, SLC22A12 | 2 | 1 | 50.00% | 0.462763533 |
| Rickets | CLCN5, CYP27B1, CYP2R1, DMP1, FGF23, SLC34A3, VDR | VDR | 1 | 1 | 100.00% | 0.241614214 |
| Blindness | CABP4, CACNA1F, CSNB1, GNAT1, GRM6, OPN1LW, OPN1MW, OPN1SW, PDE6B, RHO | AIPL1, F2, NDP | 3 | 1 | 33.33% | 0.087323627 |
| Mucopolysaccharidosis I | GALNS, IDS, IDUA | IDUA | 1 | 1 | 100.00% | 0.040395188 |
| Encephalitis | TLR3, TREX1, UNC93B1 | ICAM1, IL1B | 2 | 1 | 50.00% | 0.115044615 |
| Medulloblastoma | BRCA2, PTCH2, SUFU | BRCA2 | 1 | 1 | 100.00% | 0.202135288 |
| Keratosis | ATP2A2, DKC1, DSG1, DSP, KRT1, KRT10, NOLA3, PIK3CA, RSPO1, SART3, SAT1, TERC, TERT, TINF2 | ATP2A2, GSTM1 | 2 | 1 | 50.00% | 0.57415166 |
| Ectodermal Dysplasia | CDH3, ED1, EDAR, EDARADD, GJB6, HVEC, IKBKG, KRT85, NFKBIA, PKP1, TP63 | NFKBIA | 1 | 1 | 100.00% | 0.271173612 |
| Coloboma | CHX10, IGBP1, PAX2, PAX6, SHH | PAX2 | 1 | 1 | 100.00% | 0.186917377 |
| Angioedema | C1NH, F12, XPNPEP2 | XPNPEP2 | 1 | 1 | 100.00% | 0.116604255 |
| Cleft Lip | CDH1, HVEC, MSX1, MTHFR, MTR, TP63 | MSX1 | 1 | 1 | 100.00% | 0.286192335 |
| Pseudohypoaldosteronism | NR3C2, SCNN1A, SCNN1B, SCNN1G, WNK1, WNK4 | WNK4 | 1 | 1 | 100.00% | 0.103776592 |
| Diabetes Insipidus | AQP2, AVP, AVPR2 | AVP, CANX | 2 | 1 | 50.00% | 0.056776504 |
| Adrenal Hyperplasia, Congenital | CYP11B1, CYP21A2, POR | CYP21, CYP21A1, CYP21A2 | 3 | 1 | 33.33% | 0.342118283 |
| Amyloidosis | APOA1, APP, FGA, GSN, LYZ, OSMR, TTR | TTR | 1 | 1 | 100.00% | 0.304995618 |
| Hypogonadism | CHD7, DAX1, FGFR1, GNRHR, GPR54, LEP, LEPR, LHB, LHCGR, NELF, PROK2, SLC29A3 | GNRHR | 1 | 1 | 100.00% | 0.175165325 |

^a^: The gene symbols are the official ones nominated by HUGO Gene Nomenclature Committee (http://www.genenames.org/); ^b^: The coverage p-values are calculated according to hyper geometric distribution.
