# Supplementary material for: A Knowledge-Based Weighting Framework to Boost the Power of Genome-Wide Association Studies
Source: PLoS One. 2010 Dec 31;5(12):e14480. doi: 10.1371/journal.pone.0014480 (PMC3013112; doi:10.1371/journal.pone.0014480)
Supplement: Table S6 — (0.75 MB DOC) [file pone.0014480.s013.doc]

Table S6: SNPs with weighted p-values ≤ 5.0E-4

| **dbSNP RSID** | **Original p-Value** | **Weighted p-Value** | **Chromosome** | **Position** | **Gene Symbol** | **Entrez Gene ID** | **Gene Feature** | ^a^**Conservation Score** | ^b^**miRNA Binding Site** | ^c^**Selection Score CEU** |
| --- | --- | --- | --- | --- | --- | --- | --- | --- | --- | --- |
| rs10824310 | 0.000000419 | 5.39455E-08 | 10 | 53698470 | PRKG1 | 5592 | intron | 0 | N | - |
| rs17330779 | 8.974E-07 | 1.15539E-07 | 7 | 107670935 | NRCAM | 4897 | intron | 0 | N | - |
| rs6784615 | 0.000001269 | 1.63382E-07 | 3 | 52481466 | NISCH | 11188 | intron | 0 | N | - |
| rs7077757 | 0.000001455 | 1.87329E-07 | 10 | 112534645 | RBM20 | 282996 | intron | 0.769 | N | 0.94 |
| rs2517509 | 0.000002405 | 3.0964E-07 | 6 | 31138203 | HCG22 | 285834 | - | 0 | N | - |
| rs10747758 | 0.000002686 | 3.45818E-07 | 12 | 54287594 | OR6U2P | 390329 | - | 0.009 | N | - |
| rs17505622 | 0.000005549 | 7.14424E-07 | 13 | 101772616 | FGF14 | 2259 | intron | 0.002 | N | - |
| rs475093 | 0.00001457 | 1.87586E-06 | 1 | 43388924 | hCG_23177 | 440585 | intron | 0 | N | 1.353 |
| rs7079348 | 0.00001706 | 2.19645E-06 | 10 | 77742395 | C10orf11 | 83938 | intron | 0.008 | N | - |
| rs11748700 | 0.0000175 | 2.25309E-06 | 5 | 15776346 | FBXL7 | 23194 | intron | 0.041 | N | - |
| rs4759173 | 0.00001783 | 2.29558E-06 | 12 | 54287453 | OR6U2P | 390329 | - | 0 | N | 1.645 |
| rs950922 | 0.00001998 | 2.57239E-06 | 1 | 21752655 | ALPL | 249 | intron | 0 | N | - |
| rs9934599 | 0.0000203 | 2.61359E-06 | 16 | 69234035 | C16orf77 | 146433 | - | 0 | N | - |
| rs4394475 | 0.00002201 | 2.83375E-06 | 9 | 90499677 | LOC100128660 | 100128660 | - | 0 | N | - |
| rs17126808 | 0.00002555 | 3.28952E-06 | 8 | 18458340 | PSD3 | 23362 | intron | 0 | N | - |
| rs7817227 | 0.00002581 | 3.32299E-06 | 8 | 27973976 | C8orf80 | 389643 | missense | 0.083 | N | - |
| rs7585710 | 0.00003873 | 4.98642E-06 | 2 | 10822758 | ATP6V1C2 | 245973 | intron | 0 | N | - |
| rs17586545 | 0.00004175 | 5.37524E-06 | 14 | 51104768 | LOC100132436 | 100132436 | intron | 0 | N | - |
| rs9855470 | 0.00004253 | 5.47566E-06 | 3 | 52468315 | NISCH | 11188 | intron | 0 | N | - |
| rs12044355 | 0.00004349 | 5.59926E-06 | 1 | 229910970 | DISC1 | 27185 | intron | 0.001 | N | 0.49 |
| rs10495074 | 0.00005424 | 6.98331E-06 | 1 | 215998288 | SPATA17 | 128153 | intron | 0 | N | - |
| rs6094514 | 0.00005524 | 7.11205E-06 | 20 | 44997127 | EYA2 | 2139 | intron | 0 | N | 0.511 |
| rs7662187 | 0.0001059 | 1.36344E-05 | 4 | 158006222 | PDGFC | 56034 | intron | 0.003 | N | 0.112 |
| rs2779556 | 0.0001343 | 1.72909E-05 | 9 | 100305111 | GABBR2 | 9568 | intron | 0 | N | - |
| rs12136698 | 0.0001358 | 1.7484E-05 | 1 | 215985793 | SPATA17 | 128153 | intron | 0 | N | 1.982 |
| rs6508182 | 0.0001408 | 1.81278E-05 | 18 | 48763478 | DCC | 1630 | intron | 0 | N | 0.306 |
| rs4491988 | 0.0001429 | 1.83981E-05 | 4 | 94191325 | GRID2 | 2895 | intron | 0 | N | 0.185 |
| rs2739771 | 0.0001434 | 1.84625E-05 | 15 | 22997449 | SNRPN | 6638 | - | 0.001 | N | - |
| rs2739771 | 0.0001434 | 1.84625E-05 | 15 | 22997449 | SNRPN | 6638 | - | 0.001 | N | - |
| rs2739771 | 0.0001434 | 1.84625E-05 | 15 | 22997449 | SNRPN | 6638 | - | 0.001 | N | - |
| rs2739771 | 0.0001434 | 1.84625E-05 | 15 | 22997449 | SNRPN | 6638 | - | 0.001 | N | - |
| rs2739771 | 0.0001434 | 1.84625E-05 | 15 | 22997449 | SNRPN | 6638 | - | 0.001 | N | - |
| rs2739771 | 0.0001434 | 1.84625E-05 | 15 | 22997449 | SNRPN | 6638 | - | 0.001 | N | - |
| rs2739771 | 0.0001434 | 1.84625E-05 | 15 | 22997449 | SNRPN | 6638 | - | 0.001 | N | - |
| rs17124810 | 0.0001619 | 2.08443E-05 | 20 | 31647667 | CBFA2T2 | 9139 | intron | 0.006 | N | - |
| rs4560384 | 0.0001811 | 2.33163E-05 | 4 | 46584635 | COX7B2 | 170712 | intron | 0.003 | N | - |
| rs3767145 | 0.0001855 | 2.38828E-05 | 1 | 21768389 | ALPL | 249 | intron | 0 | N | - |
| rs4693305 | 0.0002023 | 2.60458E-05 | 4 | 94248512 | GRID2 | 2895 | intron | 0 | N | 0.131 |
| rs6018425 | 0.0002135 | 2.74878E-05 | 20 | 45423137 | ZMYND8 | 23613 | - | 0 | N | - |
| rs267526 | 0.0002236 | 2.87881E-05 | 3 | 37491140 | ITGA9 | 3680 | intron | 0 | N | 1.447 |
| rs11218016 | 0.0002255 | 2.90327E-05 | 11 | 120199964 | GRIK4 | 2900 | intron | 0.705 | N | 0.011 |
| rs16892852 | 0.0002279 | 2.93417E-05 | 8 | 120682427 | ENPP2 | 5168 | intron | 0 | N | - |
| rs17035367 | 0.0002305 | 2.96765E-05 | 4 | 158005675 | PDGFC | 56034 | intron | 0 | N | 0.558 |
| rs7313403 | 0.0002325 | 2.9934E-05 | 12 | 2418535 | CACNA1C | 775 | intron | 0 | N | - |
| rs9962633 | 0.0002361 | 3.03975E-05 | 18 | 48765464 | DCC | 1630 | intron | 0 | N | 0.172 |
| rs11583715 | 0.0002375 | 3.05777E-05 | 1 | 229895662 | DISC1 | 27185 | intron | 0 | N | 0.697 |
| rs10493007 | 0.000238 | 3.06421E-05 | 1 | 21729835 | ALPL | 249 | intron | 0 | N | - |
| rs10865972 | 0.0002461 | 3.16849E-05 | 3 | 52466487 | NISCH | 11188 | intron | 0.001 | N | - |
| rs4289558 | 0.0002485 | 3.19939E-05 | 5 | 15788014 | FBXL7 | 23194 | intron | 0.006 | N | - |
| rs7691439 | 0.000256 | 3.29596E-05 | 4 | 143663559 | INPP4B | 8821 | intron | 0.006 | N | - |
| rs3176778 | 0.0002605 | 3.35389E-05 | 12 | 9806156 | CD69 | 969 | - | 0.002 | N | 0.033 |
| rs10955311 | 0.0002609 | 3.35904E-05 | 8 | 104247753 | BAALC | 79870 | intron | 0 | N | - |
| rs864461 | 0.0002689 | 3.46204E-05 | 11 | 34688270 | LOC100132286 | 100132286 | - | 0.931 | N | - |
| rs2124622 | 0.0002768 | 3.56375E-05 | 4 | 94053969 | GRID2 | 2895 | intron | 0 | N | - |
| rs11895074 | 0.0002829 | 3.64229E-05 | 2 | 214740825 | SPAG16 | 79582 | intron | 0.002 | N | - |
| rs1845431 | 0.000285 | 3.66933E-05 | 8 | 104238024 | BAALC | 79870 | intron | 0.002 | N | - |
| rs7048496 | 0.0002925 | 3.76589E-05 | 9 | 2115231 | SMARCA2 | 6595 | intron | 0 | N | - |
| rs11145861 | 0.0002944 | 3.79035E-05 | 9 | 138408197 | SNAPC4 | 6621 | intron | 0 | N | - |
| rs910171 | 0.0002959 | 3.80966E-05 | 6 | 39126852 | GLP1R | 2740 | intron | 0.004 | N | - |
| rs4687619 | 0.0002966 | 3.81867E-05 | 3 | 52493826 | NISCH | 11188 | intron | 0 | N | - |
| rs17098236 | 0.0002975 | 3.83026E-05 | 11 | 101892901 | MMP7 | 4316 | - | 0.002 | N | - |
| rs12581163 | 0.0003015 | 3.88176E-05 | 12 | 18466821 | PIK3C2G | 5288 | intron | 0.003 | N | - |
| rs6445486 | 0.0003017 | 3.88433E-05 | 3 | 52481531 | NISCH | 11188 | intron | 0 | N | - |
| rs1477599 | 0.0003227 | 4.15471E-05 | 15 | 22881395 | SNRPN | 6638 | - | 0 | N | - |
| rs1477599 | 0.0003227 | 4.15471E-05 | 15 | 22881395 | SNRPN | 6638 | - | 0 | N | - |
| rs1477599 | 0.0003227 | 4.15471E-05 | 15 | 22881395 | SNRPN | 6638 | - | 0 | N | - |
| rs1477599 | 0.0003227 | 4.15471E-05 | 15 | 22881395 | SNRPN | 6638 | - | 0 | N | - |
| rs1477599 | 0.0003227 | 4.15471E-05 | 15 | 22881395 | SNRPN | 6638 | - | 0 | N | - |
| rs1477599 | 0.0003227 | 4.15471E-05 | 15 | 22881395 | SNRPN | 6638 | - | 0 | N | - |
| rs1477599 | 0.0003227 | 4.15471E-05 | 15 | 22881395 | SNRPN | 6638 | - | 0 | N | - |
| rs1477599 | 0.0003227 | 4.15471E-05 | 15 | 22881395 | SNRPN | 6638 | - | 0 | N | - |
| rs1477599 | 0.0003227 | 4.15471E-05 | 15 | 22881395 | SNRPN | 6638 | - | 0 | N | - |
| rs1477599 | 0.0003227 | 4.15471E-05 | 15 | 22881395 | SNRPN | 6638 | - | 0 | N | - |
| rs1477599 | 0.0003227 | 4.15471E-05 | 15 | 22881395 | SNRPN | 6638 | - | 0 | N | - |
| rs1477599 | 0.0003227 | 4.15471E-05 | 15 | 22881395 | SNRPN | 6638 | - | 0 | N | - |
| rs1523355 | 0.0003296 | 4.24354E-05 | 2 | 50181566 | NRXN1 | 9378 | intron | 0 | N | 1.507 |
| rs12643131 | 0.0003305 | 4.25513E-05 | 4 | 169804392 | PALLD | 23022 | intron | 0.038 | N | - |
| rs6091465 | 0.0003468 | 4.46499E-05 | 20 | 50221424 | ZFP64 | 55734 | intron | 0.002 | N | - |
| rs13109660 | 0.0003523 | 4.5358E-05 | 4 | 55665437 | KDR | 3791 | intron | 0.001 | N | - |
| rs4729049 | 0.0003796 | 4.88728E-05 | 7 | 92215119 | CDK6 | 1021 | intron | 0.405 | N | 1.655 |
| rs2456223 | 0.0003873 | 4.98642E-05 | 5 | 52213103 | ITGA1 | 3672 | intron | 0.006 | N | 0.223 |
| rs3829073 | 0.0003913 | 5.03792E-05 | 9 | 2117883 | SMARCA2 | 6595 | intron | 0.102 | N | 3.717 |
| rs1763351 | 0.0004095 | 5.27224E-05 | 1 | 103146026 | COL11A1 | 1301 | intron | 0 | N | - |
| rs17425467 | 0.0004112 | 5.29413E-05 | 1 | 160484013 | NOS1AP | 9722 | intron | 0 | N | - |
| rs3845973 | 0.0004312 | 5.55162E-05 | 3 | 60002946 | FHIT | 2272 | intron | 0 | N | - |
| rs6669690 | 0.0004355 | 5.60699E-05 | 1 | 216076119 | SPATA17 | 128153 | intron | 0 | N | - |
| rs6738544 | 0.0004437 | 5.71256E-05 | 2 | 191697601 | STAT4 | 6775 | intron | 0 | N | 2.107 |
| rs3793499 | 0.0004461 | 5.74346E-05 | 9 | 2121990 | SMARCA2 | 6595 | intron | 0.006 | N | 3.463 |
| rs1583802 | 0.00045 | 5.79367E-05 | 14 | 32856807 | NPAS3 | 64067 | intron | 0 | N | - |
| rs1875205 | 0.0004509 | 5.80526E-05 | 16 | 9967609 | GRIN2A | 2903 | intron | - | N | - |
| rs17526457 | 0.0004545 | 5.85161E-05 | 3 | 25574204 | RARB | 5915 | intron | 0 | N | - |
| rs7504750 | 0.0004691 | 6.03958E-05 | 18 | 48801638 | DCC | 1630 | intron | 0.31 | N | 0.745 |
| rs1538974 | 0.0004839 | 6.23013E-05 | 1 | 229895219 | DISC1 | 27185 | intron | 0.002 | N | 0.213 |
| rs897005 | 0.0004851 | 6.24558E-05 | 11 | 44240298 | ALX4 | 60529 | - | 0 | N | - |
| rs6018172 | 0.0004895 | 6.30223E-05 | 20 | 44981150 | EYA2 | 2139 | intron | 0 | N | - |
| rs6810027 | 0.0004924 | 6.33956E-05 | 3 | 52499614 | NISCH | 11188 | intron | 0 | N | - |
| rs123598 | 0.0004963 | 6.38978E-05 | 3 | 52410900 | BAP1 | 8314 | utr-3 | 0 | Y | - |
| rs13228455 | 0.0005048 | 6.49921E-05 | 7 | 39717683 | RALA | 5898 | - | 0.091 | N | - |
| rs17171612 | 0.0005302 | 6.82623E-05 | 7 | 39690989 | RALA | 5898 | intron | 0.234 | N | - |
| rs2144834 | 0.0005334 | 6.86743E-05 | 14 | 93843387 | SERPINA6 | 866 | intron | 0.001 | N | - |
| rs2779563 | 0.0005354 | 6.89318E-05 | 9 | 100317637 | GABBR2 | 9568 | intron | 0 | N | - |
| rs2506135 | 0.0005372 | 6.91636E-05 | 10 | 33505043 | NRP1 | 8829 | near-gene-3 | 0 | N | - |
| rs8177113 | 0.0005442 | 7.00648E-05 | 7 | 142264967 | EPHB6 | 2051 | intron | 0 | N | 1.84 |
| rs2779593 | 0.0005621 | 7.23694E-05 | 9 | 100335323 | GABBR2 | 9568 | intron | 0 | N | - |
| rs7869436 | 0.0005663 | 7.29101E-05 | 9 | 2117315 | SMARCA2 | 6595 | intron | 0 | N | - |
| rs7297136 | 0.0005763 | 7.41976E-05 | 12 | 53264138 | PPP1R1A | 5502 | intron | 0.001 | N | 0.009 |
| rs17169622 | 0.0005884 | 7.57555E-05 | 7 | 34015763 | BMPER | 168667 | intron | 0 | N | - |
| rs4435862 | 0.0005908 | 7.60645E-05 | 5 | 15772222 | FBXL7 | 23194 | intron | 0 | N | - |
| rs6593919 | 0.0006052 | 7.79184E-05 | 1 | 203193306 | NFASC | 23114 | intron | 0 | N | - |
| rs1307494 | 0.0006234 | 8.02617E-05 | 1 | 10432290 | APITD1 | 378708 | intron | 0 | N | 0.452 |
| rs13109080 | 0.0006324 | 8.14204E-05 | 4 | 46581333 | COX7B2 | 170712 | intron | 0 | N | - |
| rs11865044 | 0.0006392 | 8.22959E-05 | 16 | 82002886 | CDH13 | 1012 | intron | 0.002 | N | - |
| rs6787801 | 0.0006404 | 8.24504E-05 | 3 | 152582431 | P2RY12 | 64805 | intron | 0 | N | 1.33 |
| rs3760114 | 0.0007097 | 9.13726E-05 | 16 | 11260025 | SOCS1 | 8651 | - | 0 | N | - |
| rs961121 | 0.0007125 | 9.17331E-05 | 1 | 235489676 | RYR2 | 6262 | intron | 0.047 | N | - |
| rs7772264 | 0.0007137 | 9.18876E-05 | 6 | 136251441 | PDE7B | 27115 | intron | 0.202 | N | - |
| rs2225741 | 0.0007147 | 9.20164E-05 | 9 | 79413428 | GNA14 | 9630 | intron | 0 | N | - |
| rs7115850 | 0.0007221 | 9.29691E-05 | 11 | 77722719 | GAB2 | 9846 | intron | 0 | N | - |
| rs10792305 | 0.0007266 | 9.35485E-05 | 11 | 60667194 | VPS37C | 55048 | intron | 0 | N | - |
| rs4515644 | 0.000728 | 9.37287E-05 | 9 | 36912924 | PAX5 | 5079 | intron | 0 | N | 0.731 |
| rs7802100 | 0.0007364 | 9.48102E-05 | 7 | 83425851 | SEMA3A | 10371 | utr-3 | 1 | N | 1.835 |
| rs11170573 | 0.0007476 | 9.62522E-05 | 12 | 52202103 | ATF7 | 11016 | intron | 0.945 | N | - |
| rs7079141 | 0.0007659 | 9.86083E-05 | 10 | 67893739 | CTNNA3 | 29119 | intron | 0 | N | - |
| rs298063 | 0.0007712 | 9.92907E-05 | 5 | 59006375 | PDE4D | 5144 | intron | 0 | N | - |
| rs17225222 | 0.0007727 | 9.94838E-05 | 4 | 160444639 | RAPGEF2 | 9693 | intron | 0.008 | N | 0.265 |
| rs2779550 | 0.0008129 | 0.000104659 | 9 | 100291497 | GABBR2 | 9568 | intron | 0 | N | - |
| rs12711551 | 0.0008249 | 0.000106204 | 2 | 121879476 | CLASP1 | 23332 | intron | 0 | N | 2.057 |
| rs2272202 | 0.0008342 | 0.000107402 | 2 | 227622869 | COL4A4 | 1286 | intron | 0 | N | 0.107 |
| rs10081642 | 0.0008541 | 0.000109964 | 9 | 100428597 | GABBR2 | 9568 | intron | 0.006 | N | 0.024 |
| rs6555483 | 0.0009048 | 0.000116491 | 5 | 7734207 | ADCY2 | 108 | intron | 0 | N | 1.063 |
| rs11865596 | 0.0009167 | 0.000118024 | 16 | 46824332 | ABCC11 | 85320 | intron | 0 | N | 0.939 |
| rs1487155 | 0.0009197 | 0.00011841 | 8 | 32314801 | NRG1 | 3084 | intron | 0 | N | 0.736 |
| rs2315321 | 0.0009321 | 0.000120006 | 6 | 161894696 | PARK2 | 5071 | intron | 0 | N | 0.483 |
| rs6844422 | 0.0009464 | 0.000121847 | 4 | 94286276 | GRID2 | 2895 | intron | 0.011 | N | - |
| rs17477995 | 0.0009507 | 0.000122401 | 4 | 15606195 | PROM1 | 8842 | intron | 0.007 | N | - |
| rs6760211 | 0.0009787 | 0.000126006 | 2 | 122016925 | CLASP1 | 23332 | intron | 0.001 | N | - |
| rs16979358 | 0.000982 | 0.000126431 | 21 | 46791405 | DIP2A | 23181 | utr-3 | 0 | Y | - |
| rs212050 | 0.0009832 | 0.000126585 | 3 | 60010110 | FHIT | 2272 | intron | 0.011 | N | 1.731 |
| rs1978326 | 0.00101 | 0.000130036 | 7 | 78013532 | MAGI2 | 9863 | intron | 0 | N | - |
| rs17054514 | 0.001031 | 0.000132739 | 4 | 169909687 | PALLD | 23022 | intron | 0.544 | N | - |
| rs7972652 | 0.001047 | 0.000134799 | 12 | 118104423 | HSPB8 | 26353 | intron | 0 | N | - |
| rs4366301 | 0.001052 | 0.000135443 | 1 | 229872909 | DISC1 | 27185 | intron | 0 | N | - |
| rs11097354 | 0.001054 | 0.000135701 | 4 | 94153589 | GRID2 | 2895 | intron | 0.891 | N | - |
| rs12228816 | 0.001061 | 0.000136602 | 12 | 16056481 | DERA | 51071 | intron | 0.033 | N | 0.569 |
| rs10964908 | 0.001099 | 0.000141494 | 9 | 2119832 | SMARCA2 | 6595 | intron | 0.038 | N | - |
| rs9979147 | 0.001099 | 0.000141494 | 21 | 18562347 | PRSS7 | 5651 | near-gene-3 | 0 | N | 1.123 |
| rs7026505 | 0.001114 | 0.000143426 | 9 | 36988416 | PAX5 | 5079 | intron | 0 | N | 0.245 |
| rs12455617 | 0.001146 | 0.000147546 | 18 | 22684849 | AQP4 | 361 | near-gene-3 | 0 | N | - |
| rs6803618 | 0.001157 | 0.000148962 | 3 | 138106481 | NCK1 | 4690 | intron | 0 | N | 1.859 |
| rs298091 | 0.001189 | 0.000153082 | 5 | 59032360 | PDE4D | 5144 | intron | 0 | N | - |
| rs16867615 | 0.001207 | 0.000155399 | 5 | 15773075 | FBXL7 | 23194 | intron | 0 | N | - |
| rs4685153 | 0.001224 | 0.000157588 | 3 | 14416961 | SLC6A6 | 6533 | - | 0 | N | 1.293 |
| rs6499837 | 0.001236 | 0.000159133 | 16 | 55011482 | AMFR | 267 | intron | 0.001 | N | - |
| rs2267691 | 0.001245 | 0.000160292 | 7 | 139243328 | TBXAS1 | 6916 | intron | 0 | N | - |
| rs12253630 | 0.001258 | 0.000161965 | 10 | 74438930 | P4HA1 | 5033 | intron | 0.001 | N | - |
| rs6827096 | 0.001276 | 0.000164283 | 4 | 148654896 | EDNRA | 1909 | intron | 0 | N | - |
| rs10512203 | 0.001303 | 0.000167759 | 9 | 92698855 | SYK | 6850 | - | 0 | N | 0.79 |
| rs2440467 | 0.001315 | 0.000169304 | 16 | 54974548 | AMFR | 267 | intron | 0.001 | N | - |
| rs11585959 | 0.00133 | 0.000171235 | 1 | 229900341 | DISC1 | 27185 | intron | 0 | N | 0.207 |
| rs17748239 | 0.001343 | 0.000172909 | 1 | 229901524 | DISC1 | 27185 | intron | 0.061 | N | 0.489 |
| rs3850528 | 0.001348 | 0.000173553 | 20 | 34166072 | EPB41L1 | 2036 | intron | 0.004 | N | 1.034 |
| rs1291211 | 0.001357 | 0.000174711 | 20 | 61808066 | ARFRP1 | 10139 | intron | 0 | N | - |
| rs4655414 | 0.001366 | 0.00017587 | 1 | 213927856 | USH2A | 7399 | intron | 0.015 | N | - |
| rs10177679 | 0.001367 | 0.000175999 | 2 | 172528496 | HAT1 | 8520 | intron | 0.001 | N | - |
| rs2736782 | 0.001374 | 0.0001769 | 3 | 60042233 | FHIT | 2272 | intron | 0 | N | - |
| rs9881669 | 0.001389 | 0.000178831 | 3 | 60462054 | FHIT | 2272 | intron | 0.063 | N | - |
| rs17205986 | 0.001407 | 0.000181149 | 5 | 82896136 | VCAN | 1462 | intron | 0 | N | - |
| rs1778331 | 0.001421 | 0.000182951 | 10 | 22942859 | PIP4K2A | 5305 | intron | 0 | N | - |
| rs6135656 | 0.001434 | 0.000184625 | 20 | 15979257 | MACROD2 | 140733 | utr-3 | 0.007 | Y | - |
| rs3780476 | 0.001435 | 0.000184754 | 9 | 32979548 | APTX | 54840 | intron | 0.001 | N | - |
| rs10157512 | 0.001501 | 0.000193251 | 1 | 108996418 | C1orf59 | 113802 | intron | 0.003 | N | - |
| rs7033529 | 0.001512 | 0.000194667 | 9 | 2115101 | SMARCA2 | 6595 | intron | 0 | N | - |
| rs920559 | 0.001532 | 0.000197242 | 4 | 102437738 | PPP3CA | 5530 | intron | 0 | N | 0.842 |
| rs11122324 | 0.001549 | 0.000199431 | 1 | 229925804 | DISC1 | 27185 | intron | 0 | N | - |
| rs4465041 | 0.001559 | 0.000200719 | 9 | 90858998 | SHC3 | 53358 | intron | 0 | N | 0.583 |
| rs11237240 | 0.001577 | 0.000203036 | 11 | 77000453 | AQP11 | 282679 | - | 0.001 | N | - |
| rs11237240 | 0.001577 | 0.000203036 | 11 | 77000453 | AQP11 | 282679 | - | 0.001 | N | - |
| rs3784334 | 0.001585 | 0.000204066 | 15 | 66397438 | ITGA11 | 22801 | intron | 0.007 | N | - |
| rs315920 | 0.001622 | 0.00020883 | 2 | 113589489 | IL1RN | 3557 | - | 0 | N | - |
| rs4259003 | 0.001632 | 0.000210117 | 3 | 144006245 | TRPC1 | 7220 | intron | 0.012 | N | 0.697 |
| rs10982322 | 0.001639 | 0.000211018 | 9 | 116399536 | ATP6V1G1 | 9550 | intron | 0 | N | - |
| rs1551151 | 0.00164 | 0.000211147 | 2 | 122122526 | CLASP1 | 23332 | intron | 0 | N | 1.729 |
| rs7750263 | 0.00166 | 0.000213722 | 6 | 16584907 | ATXN1 | 6310 | intron | 0.011 | N | - |
| rs17140347 | 0.001709 | 0.000220031 | 3 | 121856895 | HGD | 3081 | intron | 0 | N | - |
| rs3768826 | 0.001711 | 0.000220288 | 2 | 210244573 | MAP2 | 4133 | intron | 0.071 | N | 0.793 |
| rs3781418 | 0.001716 | 0.000220932 | 10 | 126701097 | CTBP2 | 1488 | intron | 0 | N | - |
| rs7704590 | 0.001726 | 0.000222219 | 5 | 15762298 | FBXL7 | 23194 | intron | 0 | N | 0.506 |
| rs11122861 | 0.001731 | 0.000222863 | 2 | 122070080 | CLASP1 | 23332 | intron | 0.001 | N | 1.83 |
| rs298592 | 0.001763 | 0.000226983 | 5 | 17317914 | BASP1 | 10409 | intron | 0 | N | - |
| rs16867617 | 0.00178 | 0.000229172 | 5 | 15773106 | FBXL7 | 23194 | intron | 0 | N | 1.835 |
| rs9881736 | 0.001788 | 0.000230202 | 3 | 60462287 | FHIT | 2272 | intron | 0 | N | - |
| rs2779565 | 0.001823 | 0.000234708 | 9 | 100318952 | GABBR2 | 9568 | intron | 0 | N | - |
| rs2847666 | 0.001832 | 0.000235867 | 11 | 59616152 | MS4A2 | 2206 | intron | 0.147 | N | - |
| rs10498760 | 0.001861 | 0.000239601 | 6 | 45471774 | RUNX2 | 860 | intron | 0.527 | N | 0.075 |
| rs6555058 | 0.001932 | 0.000248742 | 5 | 279376 | SDHA | 6389 | intron | 0 | N | 2.095 |
| rs9820417 | 0.001939 | 0.000249643 | 3 | 7079786 | GRM7 | 2917 | intron | 0 | N | - |
| rs3765474 | 0.001947 | 0.000250673 | 6 | 161910473 | PARK2 | 5071 | intron | 0 | N | 0.333 |
| rs11119986 | 0.001973 | 0.00025402 | 1 | 210836578 | ATF3 | 467 | intron | 0.007 | N | - |
| rs10499687 | 0.002001 | 0.000257625 | 7 | 49804234 | VWC2 | 375567 | intron | 0 | N | - |
| rs16868917 | 0.002019 | 0.000259943 | 5 | 89979411 | GPR98 | 84059 | intron | 0 | N | - |
| rs7136648 | 0.002031 | 0.000261488 | 12 | 48911089 | LIMA1 | 51474 | intron | 0.945 | N | - |
| rs16918958 | 0.002048 | 0.000263676 | 9 | 33026928 | DNAJA1 | 3301 | intron | 0 | N | - |
| rs940806 | 0.002064 | 0.000265736 | 7 | 55247239 | EGFR | 1956 | - | 0.213 | N | - |
| rs10792304 | 0.002075 | 0.000267153 | 11 | 60667167 | VPS37C | 55048 | intron | 0 | N | 2.5 |
| rs10931483 | 0.002081 | 0.000267925 | 2 | 191697495 | STAT4 | 6775 | intron | 0 | N | 2.107 |
| rs17136324 | 0.002147 | 0.000276423 | 7 | 6033673 | EIF2AK1 | 27102 | intron | 0.001 | N | - |
| rs11088672 | 0.00215 | 0.000276809 | 21 | 18563001 | PRSS7 | 5651 | near-gene-3 | 0 | N | - |
| rs2071864 | 0.002162 | 0.000278354 | 6 | 36208710 | MAPK13 | 5603 | intron | 0 | N | 0.353 |
| rs17146094 | 0.002192 | 0.000282216 | 7 | 73247251 | EIF4H | 7458 | intron | 0 | N | - |
| rs2140872 | 0.002196 | 0.000282731 | 2 | 141558330 | LRP1B | 53353 | intron | 0.001 | N | 0.017 |
| rs581957 | 0.002203 | 0.000283632 | 5 | 15601576 | FBXL7 | 23194 | intron | 0 | N | 0.272 |
| rs7869667 | 0.00223 | 0.000287109 | 9 | 5732450 | KIAA1432 | 57589 | intron | 0.004 | N | 0.819 |
| rs10515155 | 0.002311 | 0.000297537 | 17 | 53836943 | RNF43 | 54894 | intron | 0 | N | - |
| rs11443 | 0.002326 | 0.000299468 | 16 | 80588949 | HSPC105 | 93517 | utr-3 | 0 | Y | - |
| rs6782856 | 0.002364 | 0.000304361 | 3 | 37833775 | ITGA9 | 3680 | intron | 0 | N | - |
| rs16854779 | 0.002399 | 0.000308867 | 1 | 229893941 | DISC1 | 27185 | intron | 0 | N | 0.697 |
| rs132277 | 0.002406 | 0.000309768 | 22 | 27862951 | KREMEN1 | 83999 | intron | 0.004 | N | - |
| rs6887277 | 0.002412 | 0.000310541 | 5 | 11144039 | CTNND2 | 1501 | intron | 0.006 | N | 1.237 |
| rs17288376 | 0.002436 | 0.000313631 | 6 | 45480196 | RUNX2 | 860 | intron | 0 | N | 0.561 |
| rs4808641 | 0.002439 | 0.000314017 | 19 | 17408292 | LOC100130519 | 100130519 | missense | 0.001 | Y | 0.033 |
| rs3789989 | 0.002532 | 0.000325991 | 12 | 78750118 | PPP1R12A | 4659 | intron | 0.055 | N | - |
| rs12936919 | 0.002574 | 0.000331398 | 17 | 53102875 | MSI2 | 124540 | intron | 0.004 | N | 1.228 |
| rs17690782 | 0.00259 | 0.000333458 | 9 | 76397342 | RORB | 6096 | intron | 0 | N | - |
| rs11935367 | 0.002611 | 0.000336162 | 4 | 169745003 | PALLD | 23022 | intron | 0 | N | 0.354 |
| rs2836524 | 0.002614 | 0.000336548 | 21 | 38867699 | ERG | 2078 | intron | 0.069 | N | 0.011 |
| rs2456206 | 0.002623 | 0.000337707 | 5 | 52214876 | ITGA1 | 3672 | intron | 0.963 | N | 0.032 |
| rs199279 | 0.002643 | 0.000340282 | 3 | 37475662 | ITGA9 | 3680 | intron | 0 | N | 1.345 |
| rs9879807 | 0.002644 | 0.00034041 | 3 | 60022140 | FHIT | 2272 | intron | 0 | N | - |
| rs11122381 | 0.002664 | 0.000342985 | 1 | 230160730 | DISC1 | 27185 | intron | 0 | N | 0.665 |
| rs6694387 | 0.002671 | 0.000343887 | 1 | 170558828 | DNM3 | 26052 | intron | 0.019 | N | - |
| rs1873518 | 0.002676 | 0.00034453 | 4 | 100684196 | C4orf17 | 84103 | - | 0.016 | N | - |
| rs1873518 | 0.002676 | 0.00034453 | 4 | 100684196 | C4orf17 | 84103 | - | 0.016 | N | - |
| rs2796460 | 0.002676 | 0.00034453 | 9 | 83391414 | TLE1 | 7088 | intron | 0.004 | N | - |
| rs835316 | 0.002688 | 0.000346075 | 4 | 69720442 | UGT2B10 | 7365 | intron | 0.001 | N | 0.15 |
| rs886177 | 0.00272 | 0.000350195 | 1 | 110058030 | GSTM5 | 2949 | intron | 0.02 | N | 1.836 |
| rs2859542 | 0.002721 | 0.000350324 | 18 | 11855892 | GNAL | 2774 | intron | 0 | N | 1.38 |
| rs3765475 | 0.002728 | 0.000351225 | 6 | 161910506 | PARK2 | 5071 | intron | 0 | N | 0.038 |
| rs8180214 | 0.002741 | 0.000352899 | 4 | 90863531 | SNCA | 6622 | - | 0.002 | N | 0.279 |
| rs11598597 | 0.002753 | 0.000354444 | 10 | 112525945 | RBM20 | 282996 | intron | 0.001 | N | - |
| rs955450 | 0.002754 | 0.000354573 | 9 | 116872927 | TNC | 3371 | intron | 0.005 | N | - |
| rs1878172 | 0.002788 | 0.00035895 | 3 | 13617871 | FBLN2 | 2199 | intron | 0.006 | N | 0.827 |
| rs4441639 | 0.0028 | 0.000360495 | 3 | 7154682 | GRM7 | 2917 | intron | 0.001 | N | 0.967 |
| rs6848015 | 0.002804 | 0.00036101 | 4 | 169684684 | PALLD | 23022 | intron | 0.001 | N | - |
| rs3804850 | 0.002824 | 0.000363585 | 3 | 7663702 | GRM7 | 2917 | intron | 0 | N | - |
| rs6736414 | 0.002842 | 0.000365903 | 2 | 182918516 | PDE1A | 5136 | intron | 0 | N | 0.667 |
| rs11214606 | 0.002842 | 0.000365903 | 11 | 112815079 | DRD2 | 1813 | intron | 0.001 | N | - |
| rs7547493 | 0.002846 | 0.000366418 | 1 | 77547821 | AK5 | 26289 | intron | 0.378 | N | - |
| rs3888293 | 0.002861 | 0.000368349 | 8 | 6786975 | DEFA4 | 1669 | - | 0.001 | N | 0.72 |
| rs1344 | 0.002864 | 0.000368735 | 1 | 145585897 | ACP6 | 51205 | coding-synonymous | 0.595 | N | - |
| rs10489296 | 0.002887 | 0.000371696 | 1 | 170419890 | DNM3 | 26052 | intron | 0.012 | N | 0.531 |
| rs7030164 | 0.002904 | 0.000373885 | 9 | 116855434 | TNC | 3371 | intron | 0 | N | - |
| rs3915499 | 0.002905 | 0.000374014 | 16 | 15818244 | MYH11 | 4629 | intron | 0.001 | N | - |
| rs7020413 | 0.002921 | 0.000376074 | 9 | 36992115 | PAX5 | 5079 | intron | 0.015 | N | 0.217 |
| rs6791542 | 0.002923 | 0.000376331 | 3 | 48762223 | PRKAR2A | 5576 | near-gene-3 | 0.443 | N | - |
| rs2239594 | 0.002972 | 0.00038264 | 2 | 169798577 | LRP2 | 4036 | intron | 0 | N | - |
| rs2131432 | 0.002978 | 0.000383412 | 17 | 1244222 | YWHAE | 7531 | intron | 0 | N | 1.52 |
| rs2844507 | 0.003003 | 0.000386631 | 6 | 31544560 | HCP5 | 10866 | - | 0 | N | 2.336 |
| rs2844507 | 0.003003 | 0.000386631 | 6 | 31544560 | HCP5 | 10866 | - | 0 | N | 2.336 |
| rs9867823 | 0.003013 | 0.000387918 | 3 | 52488067 | NISCH | 11188 | intron | 0 | N | - |
| rs675625 | 0.003038 | 0.000391137 | 11 | 60671449 | VPS37C | 55048 | intron | 0.003 | N | - |
| rs3738095 | 0.003041 | 0.000391523 | 1 | 21767582 | ALPL | 249 | intron | 0 | N | 1.701 |
| rs2356350 | 0.00305 | 0.000392682 | 2 | 191710783 | STAT4 | 6775 | intron | 0 | N | - |
| rs11052198 | 0.003074 | 0.000395772 | 12 | 32755726 | DNM1L | 10059 | intron | 0.094 | N | 2.145 |
| rs545884 | 0.003083 | 0.000396931 | 11 | 113556243 | ZBTB16 | 7704 | intron | 0 | N | 0.88 |
| rs2304561 | 0.003088 | 0.000397575 | 2 | 121933133 | CLASP1 | 23332 | intron | 0.001 | N | 2.458 |
| rs2863244 | 0.003098 | 0.000398862 | 2 | 113717336 | PAX8 | 7849 | intron | 0.001 | N | - |
| rs197729 | 0.003115 | 0.000401051 | 3 | 37470850 | ITGA9 | 3680 | intron | 0.03 | N | 0.26 |
| rs3123126 | 0.003121 | 0.000401823 | 6 | 159145224 | EZR | 7430 | intron | 0 | N | - |
| rs17565513 | 0.003123 | 0.000402081 | 14 | 32827054 | NPAS3 | 64067 | intron | 0 | N | - |
| rs4807505 | 0.003154 | 0.000406072 | 19 | 3701869 | TJP3 | 27134 | near-gene-3 | 0 | Y | - |
| rs6517434 | 0.00317 | 0.000408132 | 21 | 38109926 | KCNJ6 | 3763 | intron | 0.005 | N | 1.743 |
| rs7963345 | 0.003202 | 0.000412252 | 12 | 23989441 | SOX5 | 6660 | intron | 0.05 | N | 1.45 |
| rs17502818 | 0.003202 | 0.000412252 | 13 | 101390076 | FGF14 | 2259 | intron | 0 | N | - |
| rs2335167 | 0.003226 | 0.000415342 | 7 | 157906709 | PTPRN2 | 5799 | intron | 0.183 | N | 0.729 |
| rs1507705 | 0.003233 | 0.000416243 | 2 | 25568420 | DTNB | 1838 | intron | 0 | N | 0.326 |
| rs152562 | 0.003235 | 0.000416501 | 5 | 106869682 | EFNA5 | 1946 | intron | 0 | N | 2.469 |
| rs11196666 | 0.003257 | 0.000419333 | 10 | 84715598 | NRG3 | 10718 | intron | 0 | N | - |
| rs3923482 | 0.003259 | 0.000419591 | 12 | 7915629 | SLC2A14 | 144195 | intron | 0 | N | - |
| rs12659288 | 0.003341 | 0.000430148 | 5 | 7755167 | ADCY2 | 108 | intron | 0 | N | 0.964 |
| rs6745339 | 0.003345 | 0.000430663 | 2 | 33027056 | LTBP1 | 4052 | intron | 0 | N | - |
| rs1189320 | 0.003359 | 0.000432465 | 10 | 52807142 | PRKG1 | 5592 | intron | 0 | N | - |
| rs10811504 | 0.003377 | 0.000434783 | 9 | 2119744 | SMARCA2 | 6595 | intron | 0 | N | - |
| rs11700361 | 0.00338 | 0.000435169 | 20 | 34285729 | EPB41L1 | 2036 | - | 0.026 | N | 1.02 |
| rs11700361 | 0.00338 | 0.000435169 | 20 | 34285729 | EPB41L1 | 2036 | - | 0.026 | N | 1.02 |
| rs12403430 | 0.003445 | 0.000443538 | 1 | 65620354 | DNAJC6 | 9829 | intron | 0.079 | N | - |
| rs197722 | 0.003449 | 0.000444053 | 3 | 37479869 | ITGA9 | 3680 | intron | 0 | N | - |
| rs11957258 | 0.003463 | 0.000445855 | 5 | 80540038 | RASGRF2 | 5924 | intron | 0 | N | - |
| rs16916338 | 0.003483 | 0.00044843 | 9 | 100276125 | GABBR2 | 9568 | intron | 0.001 | N | - |
| rs1862942 | 0.003491 | 0.00044946 | 3 | 121847700 | HGD | 3081 | intron | 0.579 | N | - |
| rs12884344 | 0.003534 | 0.000454996 | 14 | 32822583 | NPAS3 | 64067 | intron | 0 | N | 1.044 |
| rs3027209 | 0.003538 | 0.000455511 | 17 | 7955141 | ALOXE3 | 59344 | intron | 0 | N | 1.237 |
| rs2394215 | 0.003554 | 0.000457571 | 10 | 67845456 | CTNNA3 | 29119 | intron | 0 | N | 1.667 |
| rs13335504 | 0.00359 | 0.000462206 | 16 | 46810474 | ABCC11 | 85320 | intron | 0.005 | N | 0.979 |
| rs2237702 | 0.003617 | 0.000465682 | 7 | 107409524 | LAMB1 | 3912 | intron | 0.003 | N | 0.109 |
| rs2282995 | 0.003621 | 0.000466197 | 7 | 92249559 | CDK6 | 1021 | intron | 0 | N | 1.617 |
| rs3134614 | 0.003625 | 0.000466712 | 1 | 40135641 | MYCL1 | 4610 | missense | 0.937 | N | 0.128 |
| rs3805818 | 0.00365 | 0.000469931 | 6 | 45503898 | RUNX2 | 860 | intron | 0 | N | - |
| rs2945398 | 0.00367 | 0.000472506 | 17 | 22912829 | KSR1 | 8844 | intron | 0 | N | - |
| rs150908 | 0.003695 | 0.000475725 | 17 | 3431117 | TRPV1 | 7442 | intron | 0.057 | N | 0.394 |
| rs3847470 | 0.003713 | 0.000478042 | 10 | 128020234 | ADAM12 | 8038 | intron | 0 | N | - |
| rs7850825 | 0.003725 | 0.000479587 | 9 | 36987896 | PAX5 | 5079 | intron | 0 | N | 0.761 |
| rs16968477 | 0.00374 | 0.000481518 | 15 | 72449440 | CYP11A1 | 1583 | - | 0 | N | 0.37 |
| rs12622810 | 0.003756 | 0.000483578 | 2 | 121820331 | CLASP1 | 23332 | intron | 0 | N | 2.337 |
| rs3774473 | 0.003809 | 0.000490402 | 3 | 53613891 | CACNA1D | 776 | intron | 0 | N | - |
| rs9653407 | 0.003835 | 0.00049375 | 2 | 121836740 | CLASP1 | 23332 | intron | 0 | N | - |
| rs13209032 | 0.00387 | 0.000498256 | 6 | 129317095 | LAMA2 | 3908 | intron | 0.013 | N | - |
| rs3024346 | 0.003879 | 0.000499414 | 6 | 6248245 | F13A1 | 2162 | intron | 0.01 | N | - |

^a^: The conservation scores are from the UCSC dataset (<http://hgdownload.cse.ucsc.edu/goldenPath/hg18/phastCons17way/>), where were calculated for alignments of 16 vertebrate genomes with Human. ^b^: “N” denotes that a SNP is not within a miRNA binding site and “Y” denotes a SNP is within a miRNA binding site. ^c^: The selection scores are from Voight et al. (2006)(Voight, et al., 2006).
